# Catalytic CO_2_ Reduction with Boron‐ and Aluminum Hydrides

**DOI:** 10.1002/cctc.201901255

**Published:** 2019-09-30

**Authors:** Daniel Franz, Christian Jandl, Claire Stark, Shigeyoshi Inoue

**Affiliations:** ^1^ Department of Chemistry Catalysis Research Center and Institute of Silicon Chemistry Technische Universität München Lichtenbergstr. 4 Garching bei München 85748 Germany

**Keywords:** Aluminum, Boron, Cations, Homogenous Catalysis, Organocatalysis

## Abstract

The previously reported dimeric NHI aluminum dihydrides **1 a**,**b**, as well as the bis(NHI) aluminum dihydride salt **9** ^+^[OTs]^−^, the bis(NHI) boron dihydride salt **10** ^+^[OTs]^−^, and the “free” bis(NHI) ligand **12** were investigated with regard to their activity as a homogenous (pre)catalyst in the hydroboration (i. e. catalytic reduction) of carbon dioxide (CO_2_) in chloroform under mild conditions (i. e. room temperature, 1 atm; NHI=N‐heterocyclic imine, Ts=tosyl). Borane dimethylsulfide complex and catecholborane were used as a hydride source. Surprisingly, the less sterically hindered **1 a** exhibited lower catalytic activity than the bulkier **1 b**. A similarly unexpected discrepancy was found with the lower catalytic activity of **10** ^+^ in comparison to the one of the bis(NHI) **12**. The latter is incorporated as the ligand to the boron center in **10** ^+^. To elucidate possible mechanisms for CO_2_ reduction the compounds were subjected to stoichiometric reactivity studies with the borane or CO_2_. Aluminum carboxylates **4**, **6**, and **7** ^+^ with two, four, and one formate group per two aluminum centers were isolated. Also, the boron formate salt **11** ^+^[OTs]^−^ was characterized. Selected metal formates were subjected to stoichiometric reactions with boranes and/or tested as a catalyst. We conclude that each type of catalyst (**1 a**,**b**, **9** ^+^, **10** ^+^, **12**) follows an individual mechanistic pathway for CO_2_ reduction.

## Graphical abstract

**CO_2_ reduction**: Aluminum‐ and boron hydrides bearing the N‐heterocyclic imino ligand show activity as (pre)catalysts in the hydroboration of carbon dioxide with borane dimethylsulfide complex and catecholborane. A bis(NHI) ligand proved as an organocatalyst with highest activity among the tested compounds. Metal formate complexes of B and Al were isolated.

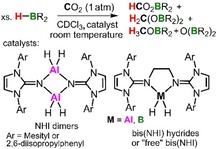

Nowadays, a chemical transformation of outstanding importance to the biosphere is the catalytic reduction of carbon dioxide. The massive amounts produced by combustion of fossil fuels are commonly acknowledged to promote climate change and sea‐water acidification.^1^ Hence, it is paramount to transform the greenhouse gas back to organic feedstock materials. This requires the use of efficient catalysts which should be environmentally benign to prevent additional stress on the ecosystem. In recent times, research in the fields of lighter main group metal(loid) catalysis^2^ and organocatalysis^3^ has produced systems that are less harmful to the environment and also contain less monetary expensive materials than comparable transition metal catalysts.

A wide scope of transition metal‐based catalysts has been established for the (photo/electro)catalytic reduction of CO_2_.^4^ A few years ago, the number of studies on organocatalytic CO_2_ transformations, particularly with regard to hydrogenation/reduction, started to surge.4b, 4f, 5 Here, strong Brønsted/Lewis bases as N‐heterocyclic carbenes (NHC, **A**) or triazabicyclodecenes (**B**, TBD) are typically implemented to promote chemical reduction of CO_2_ (Figure 1). In recent times, electron‐precise complexes of s‐ and p‐block metal(loid)s have made a similar upcoming for catalytic CO_2_ reduction.^6^ Similarly, Frustrated Lewis Pairs (**C**, FLP) have also been used as catalysts for this type of transformation.^7^ A number of aluminum cations (**D**) reported by Wehmschulte and coworkers and a non‐ionic catalytic system based on boron‐ and aluminum Lewis acids are to be pointed out, as well (Figure 1).6a, 6d, 8

**Figure 1:**
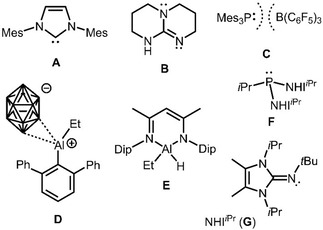
Typical examples for strong Lewis base organocatalysts NHC (**A**) and TBD (**B**). The frustrated Lewis Pair metal‐free catalyst **C**. The cationic aluminum complex **D** and the 1,3‐diketimino aluminum hydride **E**. The potent Lewis bases **F** and **G** for reversible CO_2_ binding that are both based on N‐heterocyclic imine (NHI). Mes=mesityl, Dip=2,6‐di*iso*propylphenyl, icosahedron=CHB_11_Cl_11_.

More recently, our group and others have reported the hydroboration of carbonyl functionalities promoted by aluminum hydride complexes as catalysts (Figure 2).^9^ The successful implementation of this type of compounds for the catalytic reduction of CO_2_ has, however, not been described. In 2018, the group of Aldridge outlined the reactivity of 1,3‐diketimino aluminum hydride (**E**) and selected derivatives with CO_2_, catecholborane and borane dimethylsulfide complex (Figure 1).^10^ The authors did not detail the use of their aluminum hydrides for a catalytically driven CO_2_ reduction. Notably, reactivity studies on a very similar but less sterically congested aluminum hydride with CO_2_ were presented very recently but also no catalytic process was described.^11^ In the context of CO_2_ transformations with aluminum complexes a study of Myers and Berben on catalytic dehydrogenation of formic acid to CO_2_ and H_2_ is particularly noteworthy.^12^

**Figure 2:**
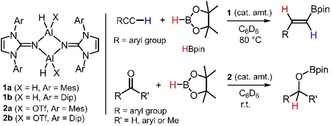
Examples (former work) for catalytic hydroboration of terminal alkynes (top) and carbonyl compounds (bottom) using imino aluminum hydrides (**1**, **2**).

Our ongoing interest in group 13 metal(loid) hydrides bearing an N‐heterocyclic imino (NHI) group^13^ as a ligand has prompted us to examine the utility of such NHI compounds as main group element catalysts for the hydroboration (i. e. reduction) of CO_2_. These compounds have been part of previous studies.9b, 14 Notably, organic superbases containing the NHI group have been described to reversibly bond to CO_2_ (**F**, **G**, Figure 1).^15^

Recently, we described the catalytic hydroboration of terminal alkynes and of carbonyl compounds (e. g. aldehydes and ketones) with pinacolborane using NHI aluminum hydrides as catalysts (Figure 2).9b Accordingly, we exposed a solution of pinacolborane in CDCl_3_ to an atmosphere of CO_2_ (1.0–1.1 bar) in the presence of catalytic amounts (1–5 mol%) of **1** or **2**. No notable conversion of the hydridoborane was recognized. Even when heating a reaction setup containing **1 b** as a (pre)catalyst to 60 °C for several hours

just traces of desired methoxyborane were detected via ^11^B NMR analysis. This agrees with the generally lower susceptibility of CO_2_ towards hydroboration because the second oxygen atom as a highly electronegative entity renders the C=O bond less electron‐rich than the one in aldehydes or ketones. Thus, we switched to borane dimethylsulfide complex as a reductant which is commonly known to be a stronger hydroboration reagent than pinacolborane. In the outcome, major transformation of CO_2_ into methoxyborane equivalents was observed within hours at ambient temperature using **1** as a catalyst (Table 1). Surprisingly, the less congested aluminum hydride (**1 a**) exhibited decreased catalytic activity as compared to the bulkier **1 b** (Table 1, Entries 1 and 2). With regard to mechanistic investigations one must note that the reaction of **1 b** with H_3_B ⋅ SMe_2_ (4 equivalents) had been reported to yield the aluminum borohydride **3** (Scheme 1).14c In CDCl_3_ solution **3** does not convert when exposed to an atmosphere of CO_2_ which indicates that initial reaction between **1 b** and the borane is not a viable pathway for the concerned catalytic reduction. In contrast, a solution of **1 b** in CDCl_3_ quantitatively reacts with CO_2_ (1 atm) within 2 hours to yield the dicarboxylate **4** (Scheme 1). The presence of two O(CO)H groups (i. e. formate) is confirmed by a singlet at 6.98 ppm in the ^1^H NMR spectrum (CDCl_3_) with 2H relative intensity. Also, we obtained a single crystal of **4** that was determined to the diformate by XRD analysis with the formate groups at the four‐membered ring in *trans*‐position relative to each other (Figure 3). After exposure of the less hindered congener **1 a** in CDCl_3_ to CO_2_ for 38 h the tetracarboxylate **6** was isolated (Scheme 1). A proton resonance at 7.32 ppm integrates to 4H indicating the introduction of four O(CO)H groups and this structural formulation was also confirmed by SCXRD study (Figure 4). The formation of the respective dicarboxylate could not be observed by NMR spectroscopy and it is believed to be elusive under these conditions. Notably, bulkier **4** dissolved in CDCl_3_ transforms into a tetracarboxylate species when kept in a CO_2_ atmosphere for an additional period of 8 days (Scheme 1). This reduced susceptibility of the “second” hydride at the aluminum center for CO_2_ insertion is in agreement with the finding that CDCl_3_ solutions of the bistriflates **2 a** and **2 b** do not react with CO_2_ on a 1 to 5 days timescale.

**Table 1:** Results on catalytic hydroboration/reduction of CO_2_ with borane dimethylsulfide complex using NHI‐based catalysts.

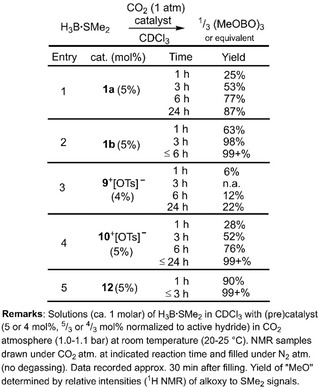

**Scheme 1:**
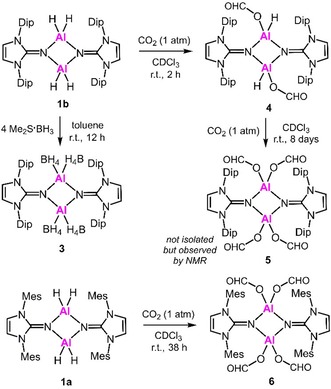
Synthesis of the aluminum borohydride **3** and the aluminum carboxylates **4**–**6** (Dip=2,6‐di*iso*propylphenyl, Mes=mesityl).

**Figure 3:**
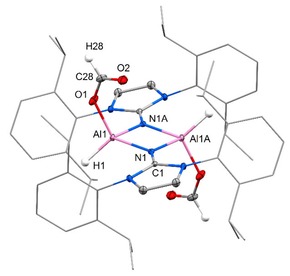
Molecular structure of **4** in the solid state as derived from SCXRD analysis (thermal ellipsoids are depicted at the 30 % level). Dip groups are depicted as wireframe model. Hydrogen atoms omitted except at Al and formate. Selected bond lengths [Å], angles [°], and atom⋅⋅⋅atom distance [Å]: Al1‐O1=1.785(1), Al1‐N1=1.882(1), Al1‐N1A=1.891(1), O1‐C28=1.287(2), O2‐C28=1.201(2), N1‐C1=1.305(2); N1‐Al1‐N1A=86.6(1), Al1‐N1‐Al1A=93.4(1), O1‐C28‐O2=128.8(1); Al⋅⋅⋅Al=2.746(1).

**Figure 4:**
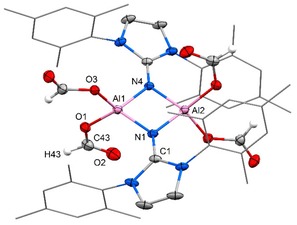
Molecular structure of **6** in the solid state as derived from SCXRD analysis (thermal ellipsoids are depicted at the 30 % level). Mesityl groups are depicted as wireframe model. Hydrogen atoms omitted except at formate. Selected bond lengths [Å], angles [°], and atom⋅⋅⋅atom distance [Å]: Al1‐O1=1.769(2), Al1‐O3=1.777(1), Al1‐N1=1.859(2), Al1‐N4=1.863(2), O1‐C43=1.294(3), O2‐C43=1.209(3), N1‐C1=1.319(2); N1‐Al1‐N4=87.6(1), O1‐Al1‐O3=101.5(1), Al1‐N1‐Al2=92.5(1), O1‐C43‐O2=125.9(2); Al⋅⋅⋅Al=2.689(1).

With the elucidation of mechanistic pathways for CO_2_ reduction and boron‐oxygen bond formation in mind, we investigated the formate group transfer capability of the aluminum carboxylate **4**. The reactions with the strong Lewis acids Ph_3_C^+^ (cationic, used as Ph_3_C^+^[Al(OR^F^)_4_]^−^) and B(C_6_F_5_)_3_ (uncharged) in CDCl_3_ solution were probed in an NMR sample tube (Scheme 2, R^F^=C(CF_3_)_3_). The trityl salt afforded a clean conversion to a new NHI species (^1^H NMR spectroscopic control) upon reaction in a one‐to‐one ratio. In contrast, two equivalents of B(C_6_F_5_)_3_ were required until the signal pattern of the proton resonances produced by the NHI ligand matched the ^1^H NMR spectrum of the trityl salt conversion of **4**. A singlet at 7.69 ppm integrates to 1H and is assigned to a formate group which resonates at significantly lower field as the carboxylate groups in **4** or **6**. Also, the formation of triphenylformylmethane and of the [HCO_2_(B(C_6_F_5_)_3_)_2_]^−^ anion, respectively, is concluded from ^1^H and ^11^B NMR analysis. As the ^1^H NMR spectrum of **7** ^+^ in CDCl_3_ suggests high symmetry for the complex we surmise the single formate group to assume a bridging position between the aluminum centers decorating the four‐membered Al_2_N_2_ ring. Consequently, we postulate the structural formulation **7** ^+^ (Scheme 2). This is confirmed via SCXRD analysis (see the SI, Figure S39). It is of note that the potential to assume an intramolecular carboxylate‐bridge structure motif as in **7** ^+^ should result in markedly different formate group donor strengths of dinuclear aluminum complexes as **4** in comparison to formates derived from mononuclear aluminum compounds of type **E**.

**Scheme 2:**
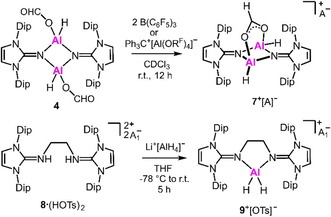
Syntheses of the cationic aluminum complexes **7** ^+^ and **9** ^+^ via formate group abstraction from **4** or dehydrogenative coupling between **8** ⋅ [HOTs]_2_ and Li^+^[AlH_4_]^−^ (A=HCO_2_(B(C_6_F_5_)_3_)_2_ or Al(OR^F^)_4_, A_1_=OTs; Dip=2,6‐di*iso*propylphenyl, Ts=tosyl, R^F^=C(CF_3_)_3_).

In order to further elucidate the mechanism for CO_2_ reduction we brought **4**, as well as **6** into contact with H_3_B ⋅ SMe_2_ (3 and 6 equiv, respectively) in CDCl_3_ in an NMR sample tube. Monitoring the progress of the reaction revealed the formation of an untraceable mixture of NHI ligand species in the ^1^H NMR in both cases (see the SI, Figures S21, S23, S25, S27). Counterintuitive to the expectation from the steric properties the bulkier **4** had decomposed completely within one hour while the less congested **6** was still observed as the major component within the same timeframe though the latter was exposed to a larger excess of the borane (7 h later **6** was found to have quantitatively disintegrated). The decomposition rates of **4** and **6** correlate to the catalytic activities of **1 b** and **1 a** for which bulkier **1 b** was also found to exhibit the higher CO_2_ conversion rate (Table 1, Entries 2 and 1). It is also to be noted that in the case of the conversion of **4** with H_3_B ⋅ SMe_2_ the ^11^B NMR analysis (after 5 h elapsed) showed two broad resonances (−37 ppm, −40 ppm) and a sharp quintet of weaker intensity (−41.5 ppm, *J*=81 Hz; note: residual H_3_B ⋅ SMe_2_ was observed at −20.4 ppm, see the SI, Figures S22, S24). The −37 ppm signal is in agreement with the value reported for the aluminum borohydride **3** while the most upfield shifted resonance can clearly be assigned to [BH_4_]^−^. Such resonances were found in the ^11^B spectrum of the conversion of **6** with excess borane, however, aside from the far slower conversion rate the relative intensity of the [BH_4_]^−^ signal was considerably increased and only traces of the two broader resonances were shown (see the SI, Figures S26, S28). The formation of [BH_4_]^−^ is of particular interest because traces of Na[BH_4_] had been reported to catalyze the reduction of CO_2_ to trimethoxyboroxine with H_3_B ⋅ thf in THF.^16^

The reactivity towards carboxylate group acceptor reagents (i. e. synthesis of **7** ^+^) shows that dimeric aluminum complexes of type **4** may readily act as carboxylate group transfer agents. However, after consideration of the reaction profile of **4** towards excess H_3_B ⋅ SMe_2_ (*vide supra*) we do not propose that the respective complex **7** ^+^ constitutes a relevant intermediate in the reduction of CO_2_ with this borane and **1 b** as a precatalyst. Taking into account the conversions described above we conclude that the catalytic CO_2_ reduction with **1** commences with insertion of CO_2_ into the Al−H bonds rather than initial reaction with the borane because a resulting complex of type **3** would be an ending path. It is conceivable, however, that a mixed Al(H)BH_4_ species promotes CO_2_ reduction but its existence could not be verified albeit the assigned ^11^B NMR signal at −40 ppm from conversions of **4** or **6** with excess borane (*vide supra*) could be produced by such type of complex. Nevertheless, the absence of CO_2_ insertion to occur for **2** and **3** renders this “mixed‐species‐pathway” unlikely. Considering that aluminum carboxylate reactions with exc. H_3_B ⋅ SMe_2_ lead to ill‐defined product mixtures supports speculations that a non‐aluminum‐containing compound promotes CO_2_ reduction and it is likely to include the tetrahydroborate anion as a potent hydride transfer group.

Very recently, we had reported a cationic aluminum dihydride complex bearing a bis(NHI) ligand with mesityl substituents at the imidazoline nitrogen atoms of the ligand.14a In the light of our study of **4** and **7** ^+^ we conceived that the bulkier bis(NHI) aluminum dihydride **9** ^+^ (with Dip instead of mesityl groups, Dip=2,6‐di*iso*propylphenyl) would be a suitable target to provide insight into (i) the difference between complexes with one Al center (**9** ^+^) and two Al centers (**1**), and (ii) the difference between cationic and uncharged aluminum dihydrides with regard to catalytic activity for CO_2_ reduction. Compound **9** ^+^[OTs]^−^ readily forms upon conversion of the bis(iminiumtosylate) **8** ⋅ (HOTs)_2_ with lithium aluminum hydride as concluded from ^1^H and ^13^C{^1^H} NMR spectroscopy and verified by SCXRD study and elemental analysis (Scheme 2, see the SI Figure S40, Ts=tosyl=*p*‐tolylsulfonyl). We tested the suitability of **9** ^+^[OTs]^−^ as a (pre)catalyst for CO_2_ reduction (i. e. hydroboration) with H_3_B ⋅ SMe_2_ and it exhibited substantially lower activity as **1 a** (Table 1, Entries 3 and 1). Presumably, the decreased activity of **9** ^+^ is connected to the lower hydride‐donor character of the cationic system as compared to uncharged **1**

We had previously described the bis(NHI) substituted boron dihydride salt **10** ^+^[OTs]^−^ (Scheme 3).14d It was in order to include this compound in this study due to its obvious structural resemblance to **9** ^+^[OTs]^−^ and because of our ongoing interest in comparing the reactivities of borohydrides and aluminum hydrides. The reaction of **10** ^+^[OTs]^−^ in CDCl_3_ with CO_2_ (1.0–1.1 bar) furnished the borocarboxylate **11** ^+^[OTs]^−^ within 12 h (Scheme 3). Thus, the reactivity of the boron dihydride is reminiscent to the one of the aluminum hydrides **1 a**,**b** and the compound might be of use for catalytic CO_2_ hydroboration, as well (*vide infra*). The introduction of the carboxylate group at boron is indicated by the rise of a singlet at 5.51 ppm in the proton NMR spectrum (CDCl_3_) that integrates to 1H. A signal at −1 ppm (*J*_BH_ not resolved) in the ^11^B NMR analysis is shifted to lower field with regard to the one of the precursor (−9 ppm, CD_3_CN) and suggests that the boron nucleus remains four‐coordinate but has one hydride replaced by a more electron‐withdrawing ligand. Moreover, the structural formulation is established by SCXRD analysis (see the SI, Figure S41). Continued exposition of **11** ^+^[OTs]^−^ in CDCl_3_ to CO_2_ did not result in further transformation (i. e. to the borodicarboxylate) over 24 h which might reflect the generally weaker hydride donor character with respect to the one of aluminum hydrides (**1**).

**Scheme 3:**
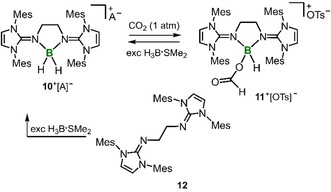
Synthesis of the boron formate **11** ^+^ and its retransformation to the boron dihydride **10** ^+^. The bis(NHI) **12** and its conversion to **10** ^+^ (Mes=mesityl, Ts=tosyl, NHI=N‐heterocyclic imine, A=OTs (top path) or BH_4_ (bottom path)).

Catalytic reduction of CO_2_ with H_3_B ⋅ SMe_2_ and use of the boron dihydride salt **10** ^+^[OTs]^−^ as a catalyst resulted in a conversion rate similar to the one of aluminum hydride **1 a** (Table 1, Entries 1 and 4). In order to gain further insight into the mechanism of CO_2_ reduction using **10** ^+^ we reacted **11** ^+^[OTs]^−^ with an excess of H_3_B ⋅ SMe_2_ (7 equiv) in CDCl_3_. It occurred that in the

^1^H NMR spectrum a clean transformation to **10** ^+^ was indicated. In addition, a singlet at 3.65 ppm appeared which suggests the formation of a methoxy group. The ^11^B NMR spectrum shows a broad resonance at −8 ppm assigned to **10** ^+^ (*J*_BH_ not resolved) and a signal at 19 ppm which hints towards the formation of a trialkoxy boron species. This well‐defined conversion of **11** ^+^ to **10** ^+^ with excess H_3_B ⋅ SMe_2_ is in sharp contrast to the respective reactions of the aluminum formates **4** and **6**. It reveals that **10** ^+^ can be transformed by CO_2_ and replenished by the reductant (i. e. H_3_B ⋅ SMe_2_).

The proposed mechanism for the catalytic CO_2_ reduction with **10** ^+^ is outlined in Scheme 4. The boron dihydride is transformed to the boron formate **11** ^+^ via CO_2_ insertion into the B−H bond. With concomitant release of boron formate the catalyst (**10** ^+^) is reformed via metathesis reaction between hydridoborane (i. e. reducing agent) and **11** ^+^ (Path A). Alternately, the carbonyl group in **11** ^+^ can be hydroborated by the reducing agent to produce **Int^1^** which liberates boronic acetal upon reaction with hydridoborane (Path B).

**Scheme 4:**
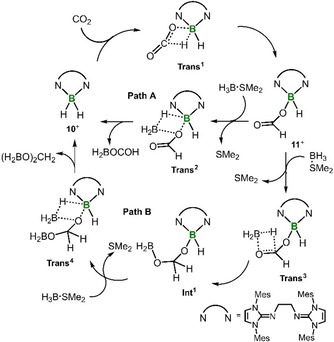
Suggested catalytic cycle for the reduction of CO_2_ with H_3_B ⋅ SMe_2_ using **10** ^+^[OTs]^−^ as a catalyst. Cationic charge omitted for clarity. A reminiscent mechanism might apply to the use of catecholborane as hydride source.

In 2018 we had published a study on the auto‐ionization of the “free” bis(NHI) ligand **12** with H_3_B ⋅ SMe_2_ (2 equiv) to **10** ^+^[BH_4_]^−^ (Scheme 3).14a In fact this transformation resembles the process when solubilized 1,8‐bis(dimethylamino)naphthalene is brought into contact with H_3_B ⋅ SMe_2_ as reported by Fontaine and coworkers.5d The authors verified that this bis(amino) compound, which may be classified as an organic superbase, can be used for catalytic reduction of CO_2_ by H_3_B ⋅ SMe_2_. Thus, it does not come as a surprise that we found the applicability of **12** for the very same purpose. Within 3 hours a solution (1 molar) of H_3_B ⋅ SMe_2_ in CDCl_3_ containing 5 mol% (i. e. ^5^/_3_ mol % referred to active B−H functionalities) of **12** is near‐quantitatively converted to alkoxyborane equivalents at 1.0–1.1 bar CO_2_ pressure (Table 1, Entry 5). The conversion rate exceeds the one observed for the use of aluminum hydride **1 b** (Table 1, Entry 2). Interestingly, the catalytic cycle suggested by Fontaine and coworkers for the use of the bis(amino)naphthalene comprises the formation of a boronium dihydride species of type **10** ^+^ but no boron carboxylate species of type **11** ^+^ is suggested. This observation raises the question if the action of **10** ^+^[OTs]^−^ as a catalyst relies on the intermediate formation of boron formate **11** ^+^ or on ligand detachment and the provision of **12** as the active species. Given that the conversion rate for the use of **10** ^+^[OTs]^−^ significantly differs from the one for the use of **12** we consider a boron‐centered mechanism as outlined in Scheme 4 viable (for **10** ^+^[OTs]^−^) that differs from the mechanism for the use of a bidentate organic superbase (e. g. 1,8‐bis(dimethylamino)naphthalene, **12**) as proposed by Fontaine.5d Taking into account that **12** can form **10** ^+^ when reacted with H_3_B⋅SMe_2_ we assume that its activity as a (pre)catalyst might rely on a dual mechanism running in part via **11** ^+^ (Scheme 4) and in part following the catalytic cycle of Fontaine which does not involve the formation of an analoguous bis(amino) boron formate.5d

As **12** had turned out to exhibit the highest catalytic activity we also used it in combination with catecholborane (HBcat) and 9‐BBN−H (9‐borabicyclo[3.3.1]nonane, HBBN) as alternate reductants. These boranes are commonly acknowledged to be less potent hydroboration agents than H_3_B ⋅ SMe_2_. Still, when using **12** as a (pre)catalyst we observed near‐quantitative conversion of either borane within 13 h when exposed to a CO_2_ atmosphere (1.0–1.1 bar) in CDCl_3_. In case of HBcat ^1^H and ^11^B NMR analysis confirmed the formation of H_3_COBcat and O(Bcat)_2_ (presumably along with H_2_C(OBcat)_2_ and HCO_2_Bcat, see the SI). When the dialkylborane is used the NMR analysis suggested the formation of a mixture of H_3_COBBN, O(BBN)_2_, H_2_C(OBBN)_2_, and HCOO(BBN) similar to the report of Cantat and coworkers on the analogous conversion using various organic nitrogen bases as catalysts (see the SI, Figures S37, S38).^17^ Given that **12** promotes the CO_2_ reduction with HBcat we also probed selected boron and aluminum complexes as catalysts for comparison. The results are outlined in Table 2. In accordance with the decreased hydroboration activity of HBcat in comparison to H_3_B ⋅ SMe_2_ the conversions generally take longer and a lower ratio of methoxy containing products is obtained. Unsurprisingly, the relative catalytic activity of the compounds follows the trend from the trihydridoborane reactions (Table 1) with **12** being the most potent catalyst and **9** ^+^ showing the by far lowest conversion rates. This suggests that a similar mechanism for the CO_2_ hydroboration is at work for either reducing agent.

**Table 2:** Results on catalytic hydroboration/reduction of CO_2_ with catecholborane using NHI‐based catalysts.

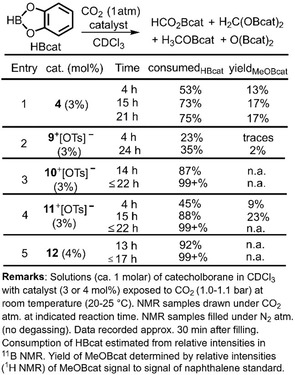

In summary, we have demonstrated the applicability of various aluminum and boron hydride complexes **1 a**,**b**, **9** ^+^, **10** ^+^, **11** ^+^_,_ as well as the organic superbase **12** for the catalytic reduction of CO_2_ with H_3_B ⋅ SMe_2_, catecholborane, and 9‐BBN−H as a hydride source. In this regard, the “free” bis(NHI) ligand **12** was found to be the most active (pre)catalyst. The bulkier aluminum hydride **1 b** exhibited comparable conversion rates while the less congested aluminum hydride **1 a** was significantly less active. The bis(NHI) aluminum dihydride salt **9** ^+^[OTs]^−^ possessed lower activity than **1 a**. The boron dihydride salt **10** ^+^[OTs]^−^ proved to be a far more potent catalyst than its cationic aluminum congener (**9** ^+^). The metal(loid) hydrides (**1**, **10** ^+^) were demonstrated to form metal formate complexes (**4**–**7** ^+^, **11** ^+^) upon conversion with CO_2_. The tetracarboxylate **6**, the dicarboxylate **4**, and the monocarboxylates **7** ^+^[HCO_2_(B(C_6_F_5_)_3_)_2_]^−^, as well as **11** ^+^[OTs]^−^ were isolated at room temperature under an atmosphere of argon or nitrogen. The aluminum formates **4** and **6** were shown to form ill‐defined product mixtures upon reaction with an excess H_3_B ⋅ SMe_2_. From the boron formate **11** ^+^ the dihydride **10** ^+^ was replenished by reaction with H_3_B ⋅ SMe_2_. For **1 a**,**b** it is hypothesized that an aluminum formate is initially formed which converts with the borane to a complex product mixture that contains the actual catalytically active species. For **10** ^+^ the relevant processes (e. g. CO_2_ insertion, σ‐bond metathesis) are supposed to majorly occur at the bis(NHI)‐bonded boron center. For **12** the [BH_4_]^−^ anion formed via auto‐ionization between bis(NHI) and H_3_B ⋅ SMe_2_ is believed to function as the hydride transferring species.

## Conflict of interest

The authors declare no conflict of interest.

## Supporting information

As a service to our authors and readers, this journal provides supporting information supplied by the authors. Such materials are peer reviewed and may be re‐organized for online delivery, but are not copy‐edited or typeset. Technical support issues arising from supporting information (other than missing files) should be addressed to the authors.

Supplementary
