# Supplementary material for: Catalytic CO2 Reduction with Boron‐ and Aluminum Hydrides
Source: ChemCatChem. 2019 Sep 30;11(21):5275–81. doi: 10.1002/cctc.201901255 (PMC6919925; doi:10.1002/cctc.201901255)
Supplement: Supplementary file 1 — Supplementary [file CCTC-11-5275-s001.pdf]

CHEMCATCHEM

Supporting Information

© Copyright Wiley-VCH Verlag GmbH & Co. KGaA, 69451 Weinheim, 2019

Catalytic CO_2 Reduction with Boron- and Aluminum Hydrides

Daniel Franz, Christian Jandl, Claire Stark, and Shigeyoshi Inoue*© 2019 The Authors. Published by Wiley-VCH Verlag GmbH & Co. KGaA.
This is an open access article under the terms of the Creative Commons Attribution License, which permits use, distribution and reproduction in any medium, provided the original work is properly cited.This manuscript is part of the Special Issue on New Concepts in Homogeneous Catalysis.

Contents

1.) Experimental Details – Synthesis and Isolation 3

2.) Experimental Details – Catalysis Study 28

3.) X-Ray Crystallographic Details 55

4.) Supplementary References 69

1.) Experimental Details – Synthesis and Isolation

General considerations: All experiments and manipulations were carried out under dry oxygen-free nitrogen using standard Schlenk techniques and glassware or in an MBraun glovebox workstation containing an atmosphere of purified argon if not stated otherwise. For stirring, PTFE-coated magnetic stirrer bars were used. Liquid phases were transferred using standard PE/PP syringes equipped with stainless steel cannula or directly canted from vessel to vessel if not stated otherwise. Solvents were dried by standard methods (e.g. withdrawal from MBraun Solvent Purification System, storage over molecular sieves (3 Å), degassing via freeze-pump-thaw cycling, distillation from sodium/ketylradical or CaH_2). Carbon dioxide gas cylinders (5.0 grade, 99.999% Vol) were supplied by Westfalen AG. Reagents were purchased from commercial suppliers and processed as received if not stated otherwise. The reagents **1a**,[S1] **1b**,[S2] $I^{Dip}NH$,[S3] and $\mathbf{10}^{+}[OTs]^{-}$[S4] were synthesized following the literature procedures. Trityl "Krossing's salt" ($Ph_3C^+[Al(OC(CF_3)_3)_4]^-$) was purchased from IoLiTec Ionic Liquids Technologies GmbH. ^{1}H, ^{11}B, ^{13}C NMR spectra were recorded on Bruker Avance 300 MHz or 400 MHz spectrometers and referenced to residual solvent signals as internal standards (^{1}H and ^{13}C) or an external standard ($Et_2O{\cdot}BF_3$ for ^{11}B). Values for the chemical shift (δ) are given in parts per million. Assignment of ^{13}C resonances was mostly supported by $^{1}H^{13}C$-HSQC or $^{1}H^{13}C$-HMQC experiments. Elemental analyses were carried out by the microanalytical laboratory of the Catalysis Research Center, Technische Universität München.
Abbreviations: s = singlet, d = doublet, t = triplet, sept = septet, br = broad, n.a. = not applicable/no answer, n.r. = not resolved, n.o. = not observed, ps = pseudotriplet, I^{Dip} = 1,3-bis(2,6-di*iso*propylphenyl)-imidazolin-2-ylidene, Ts = *p*-tolylsulfonyl, SCXRD = Single Crystal X-ray diffraction, HSQC = Heteronuclear Single Quantum Coherence/Correlation, HMQC = Heteronuclear Multiple Quantum Coherence/Correlation.

Procedure for the Isolation of {$I^{Dip}NAl(H)CO_2H$}$_2$ (4)

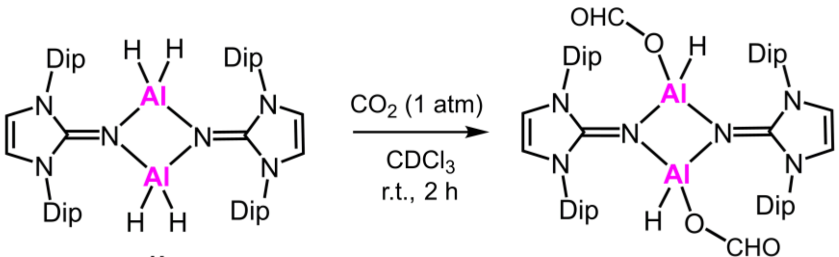

504 mg of **1b** (0.58 mmol) were dissolved in 6 mL $CDCl_3$ in a 50 mL Schlenk flask. The solution was frozen at liquid nitrogen temperature and the flask evacuated. It was pressurized with CO_2 (1.0-1.1 bar) while being thawed in a water bath (fresh from "non-warm" tap). After 2 h of stirring at room temperature, the solvent is removed *in vacuo* to afford an off-white solid. The crude product was dissolved in THF (ca. 10 mL) and a colorless solid precipitated upon addition of pentane (ca. 40 mL) which was separated. After drying *in vacuo*, 430 mg of a colorless powder were isolated (0.45 mmol, 78%).

^{1}H NMR (400.1 MHz, $CDCl_3$): δ = 7.30 (t, 4H, J = 7.7 Hz, DipH-4), 7.10 (d, 8H, J = 7.8 Hz, DipH-3,5), 6.98 (s, 2H, O_2C*H*), 6.24 (s, 4H, NC*H*), 2.66 (sept, 8H, J = 6.8 Hz, C*H*(CH_3)$_2$), 2.04 (br, 2H, Al*H*), 1.22 (d, 24H, J = 6.7 Hz, CH(C*H*$_3$)$_2$), 1.04 (d, 24H, J = 6.8 Hz, CH(C*H*$_3$)$_2$). **^{13}C{^{1}H} NMR** (100.6 MHz, $CDCl_3$): δ = 162.5 (O_2*C*H), 147.7 (ArC), 132.1 (ArC), 130.7 (ArC), 124.7 (ArC), 116.9 (N*C*H), 28.9 (*C*H(CH_3)$_2$), 25.5 (CH(*C*H$_3$)$_2$), 22.2 (CH(*C*H$_3$)$_2$), n.a. (ArC).

Elemental analysis: calcd (%) for **4**, $C_{56}H_{76}Al_2N_6O_4$, [951.23]: C 70.71, H 8.05, N 8.84; found: C 70.45, H 8.03, N 8.93.

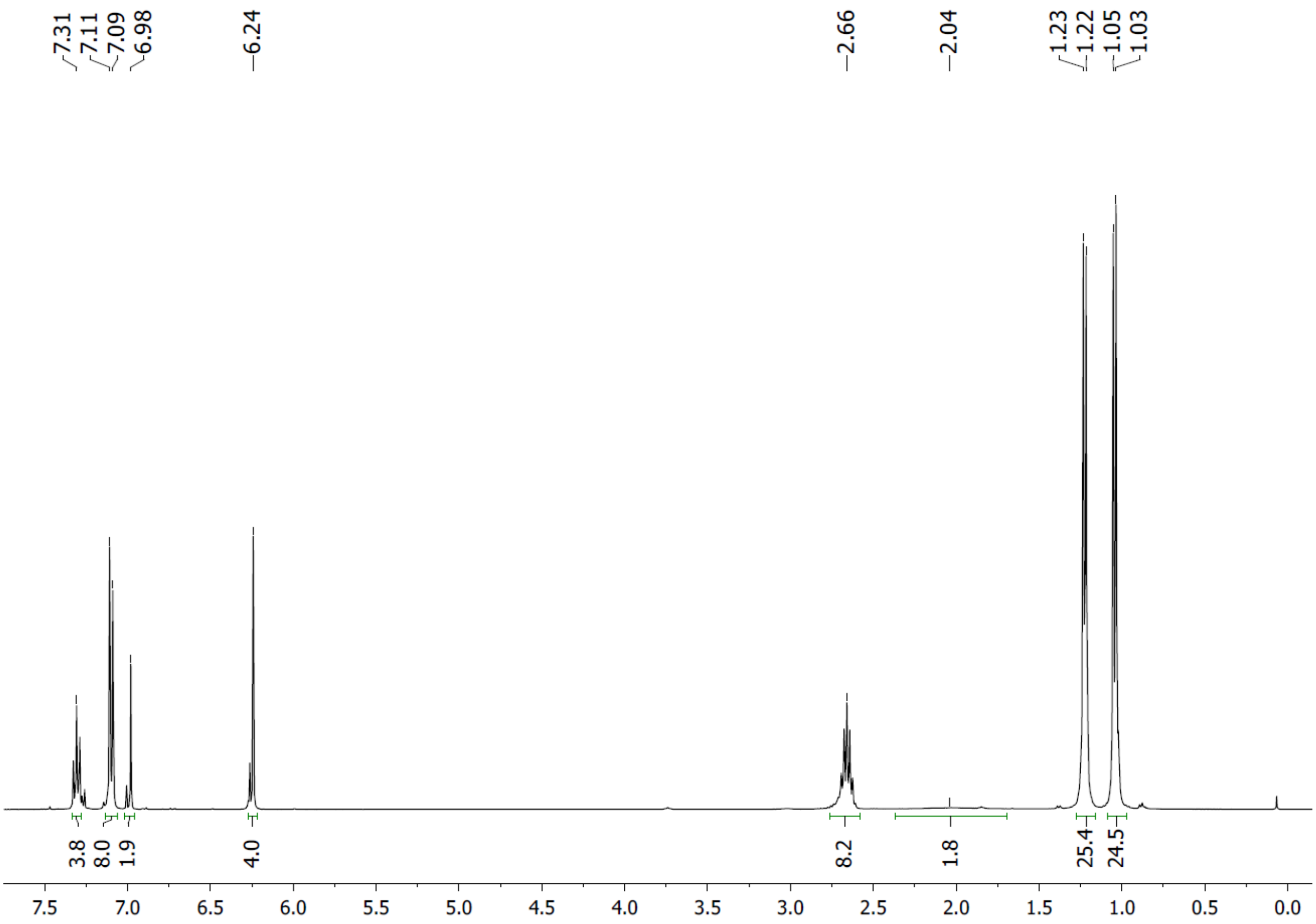

Figure S1. ^{1}H NMR spectrum (400.1 MHz, $CDCl_3$) of **4**.

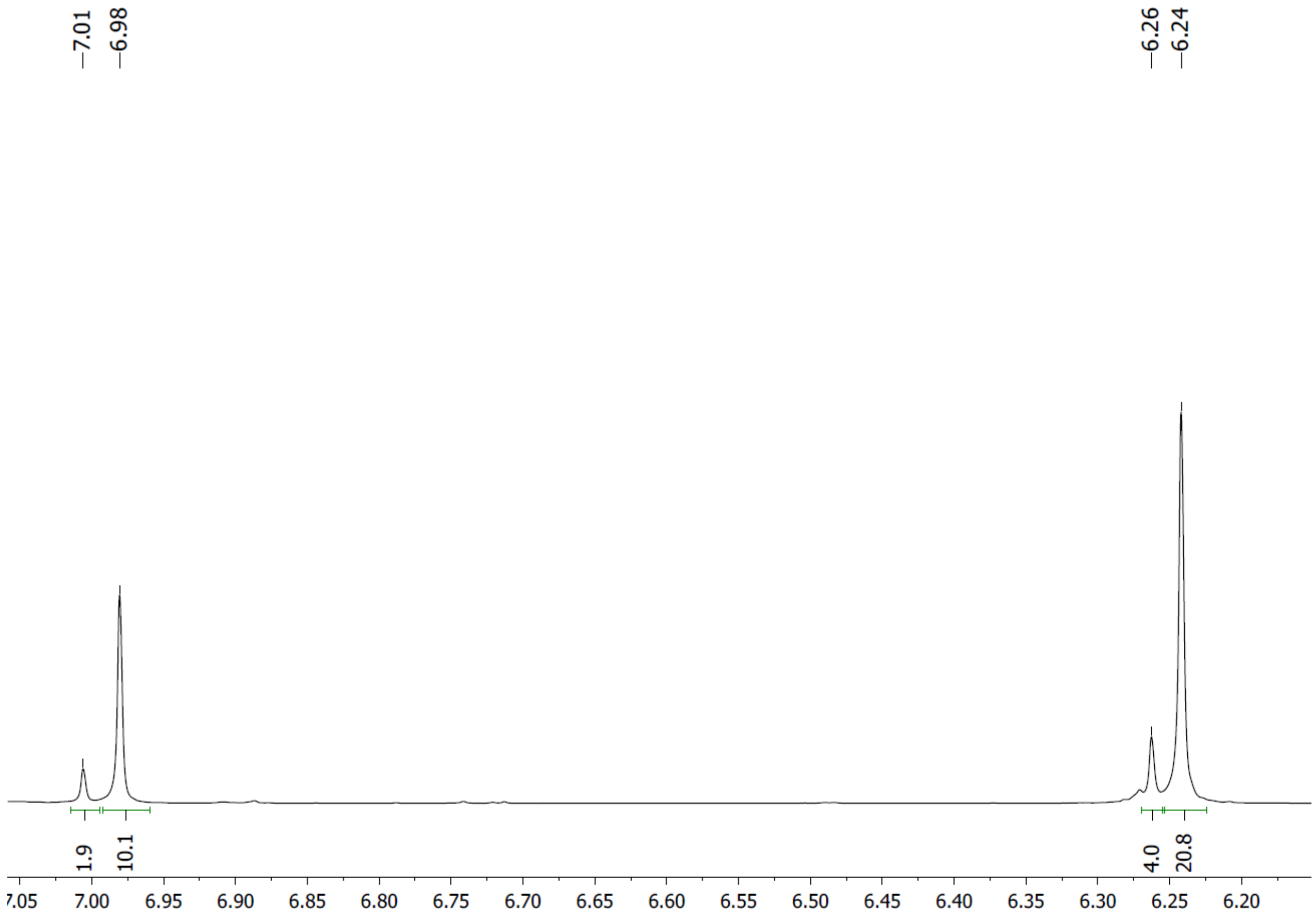

Figure S2. Expansion of the ^{1}H NMR spectrum from Figure S1 to show selected signals of the assumed *cis*-isomer as a minor component (NC*H* at 6.26 ppm and CO_2*H* at 7.01 ppm).

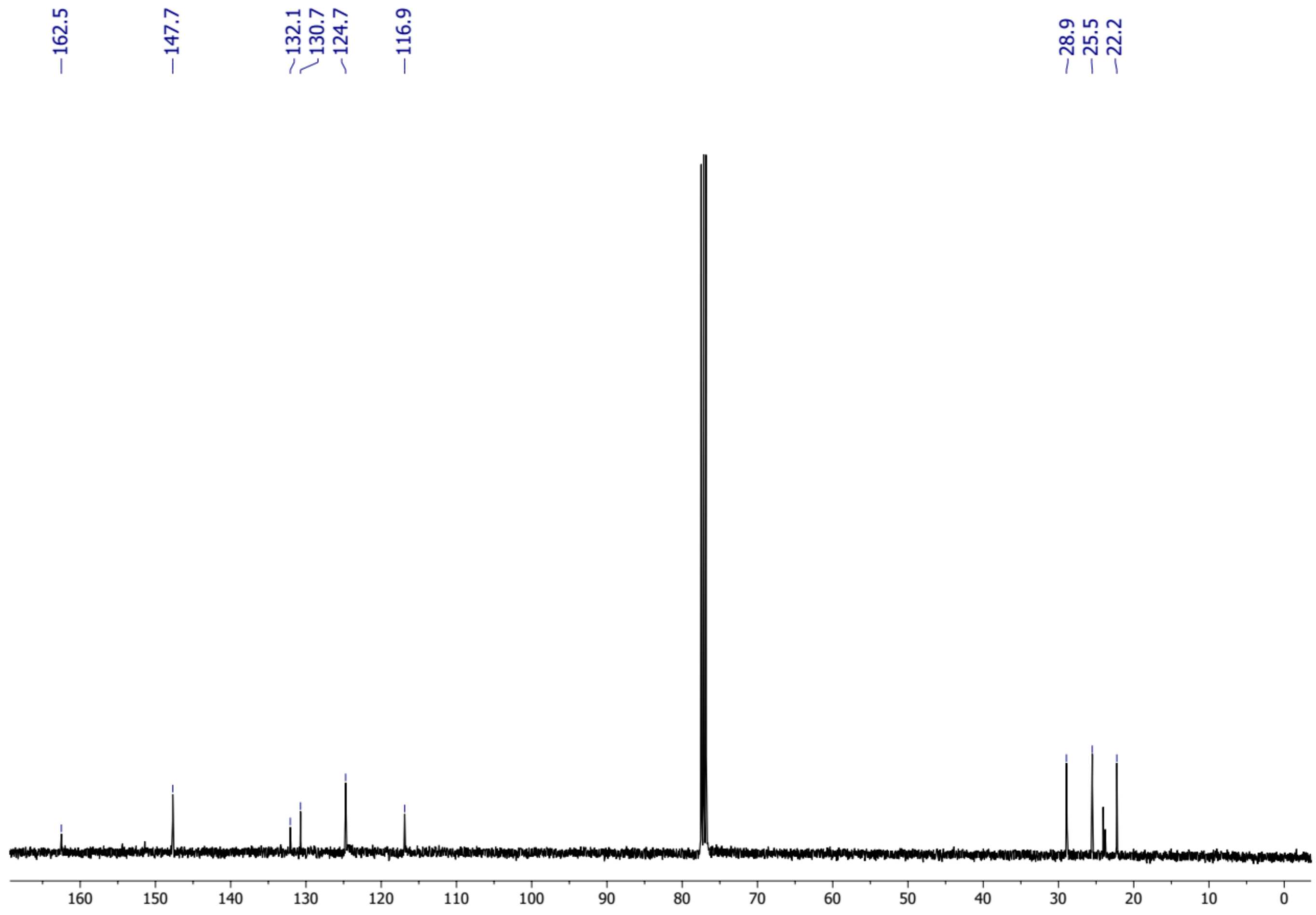

Figure S3. $^{13}C\{^1H\}$ NMR spectrum (100.6 MHz, $CDCl_3$) of **4**.

Procedure for the Synthesis of $\{I^{Dip}NAl(CO_2H)_2\}_2$ (5)

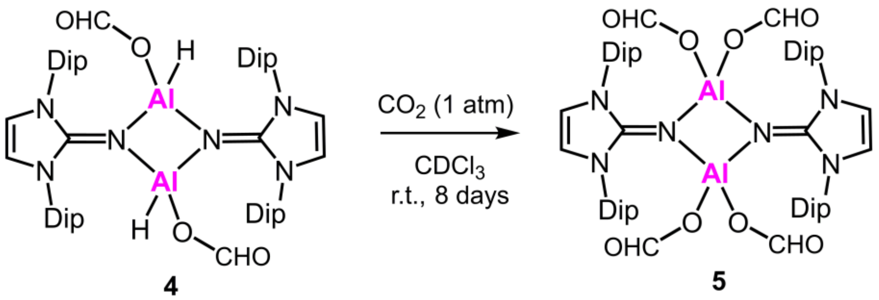

4 **5**

A reaction reaction mixture as for the isolation of **4** was continuously exposed to CO_2 over 8 days with frequent repressurizing of the CO_2 atmosphere (1.0-1.1 bar) within the otherwise sealed flask. After the indicated period the ^{1}H NMR spectrum in $CDCl_3$ (i.e. the reaction medium) revealed complete conversion of **4** to **5** and the latter is marked by a characteristic formate HCO_2 signal at 7.47 ppm.

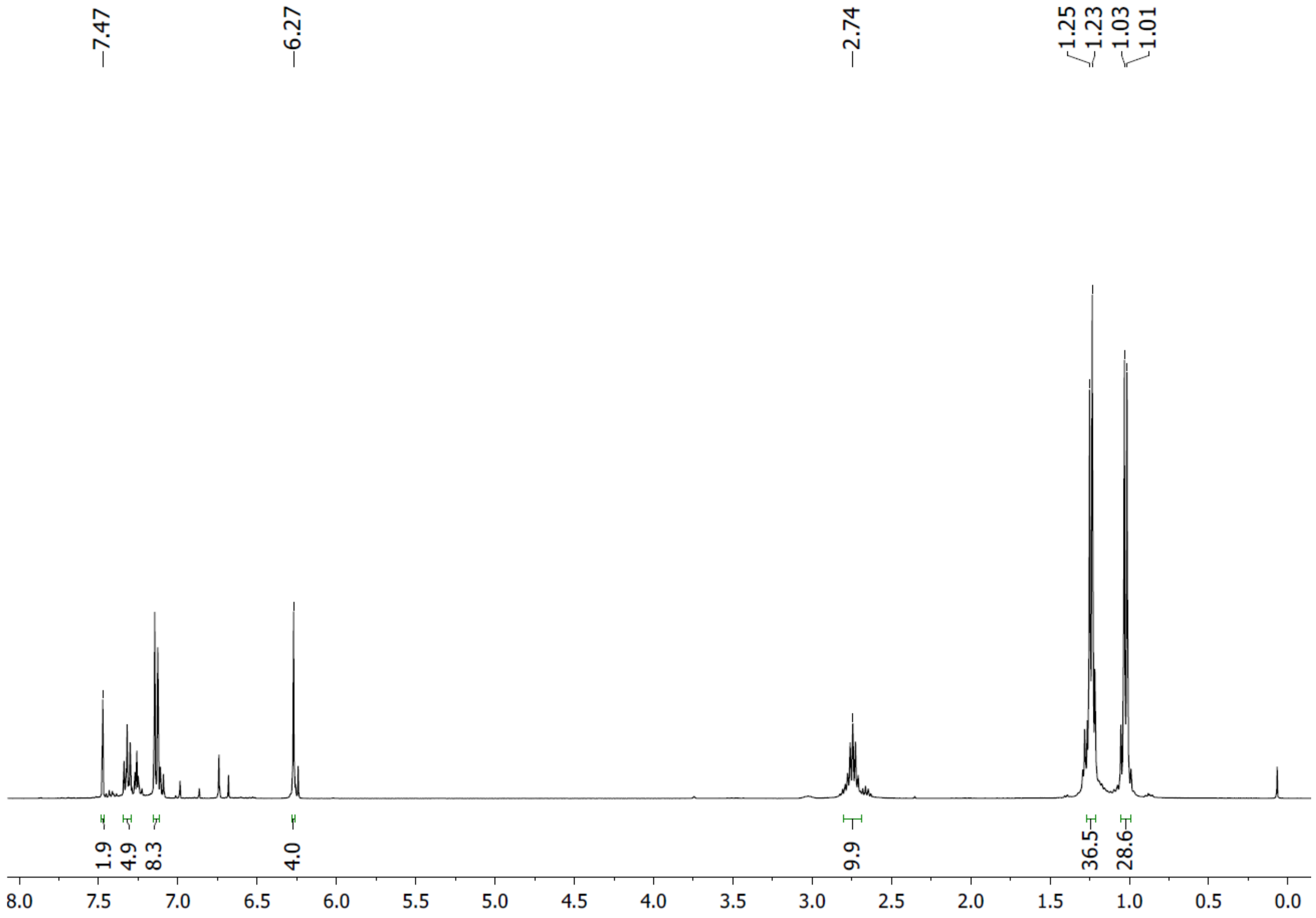

Figure S4. ^{1}H NMR spectrum (400.1 MHz, $CDCl_3$) after prolonged exposition of **4** to CO_2 atmosphere showing presumed **5** as the major component.

Procedure for the Isolation of $\{I^{Mes}NAl(CO_2H)_2\}_2$ (6)

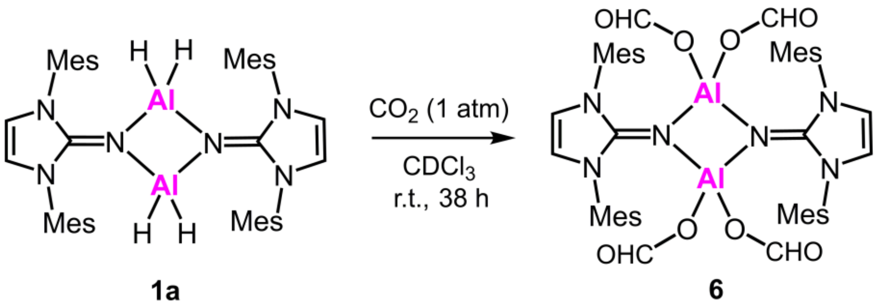

In a 50 mL Schlenk flask 495 mg of **1a** (0.71 mmol) were dissolved in $CDCl_3$. The solution is frozen at liquid nitrogen temperature and the flask was evacuated. It was pressurized with CO_2 (1.0-1.1 bar) while being thawed in a water bath (fresh from "non-warm" tap). The reaction mixture was stirred in the sealed flask and after 18 h it was repressurized with CO_2 atmosphere (1.0-1.1 bar). After stirring at room temperature for a total period of 38 h the volatiles were removed *in vacuo*. The crude product was suspended in Et_2O (5 mL) and THF was added until a clear solution was obtained (ca. 13 mL). The product was crystallized at −30 °C. The cold supernatant was withdrawn from the sedimented solid and the crystalline material was dried *in vacuo*. From the glass vessel 356 mg of product (**6**) were collected (0.41 mmol, 58%). Crystals suitable for SCXRD analysis were grown by slow diffusion of solvent vapor from a pentane/toluene mixture into a solution of **6** in $CDCl_3$.

^{1}H NMR (400.1 MHz, $CDCl_3$): δ = 7.32 (s, 4H, O_2CH), 6.89 (s, 8H, MesH-3,5), 6.22 (s, 4H, NC*H*), 2.29 (s, 12H, Mes-CH_3-4), 2.02 (s, 24H, Mes-CH_3-2,6). **^{13}C{^{1}H} NMR** (100.6 MHz, $CDCl_3$): δ = 161.8 (O_2CH), 149.3 (N*C*N), 140.8 (MesC), 137.3 (MesC), 130.1 (MesC), 130.0 (MesC-3,5), 116.1 (N*C*H), 21.2 (MesCH_3-4), 18.1 (MesCH_3-2,6).

Elemental analysis: calcd (%) for **6**, $C_{46}H_{52}Al_2N_6O_8$, [870.92]: C 63.44, H 6.02, N 9.65; found: C 63.49, H 6.33, N 9.43.

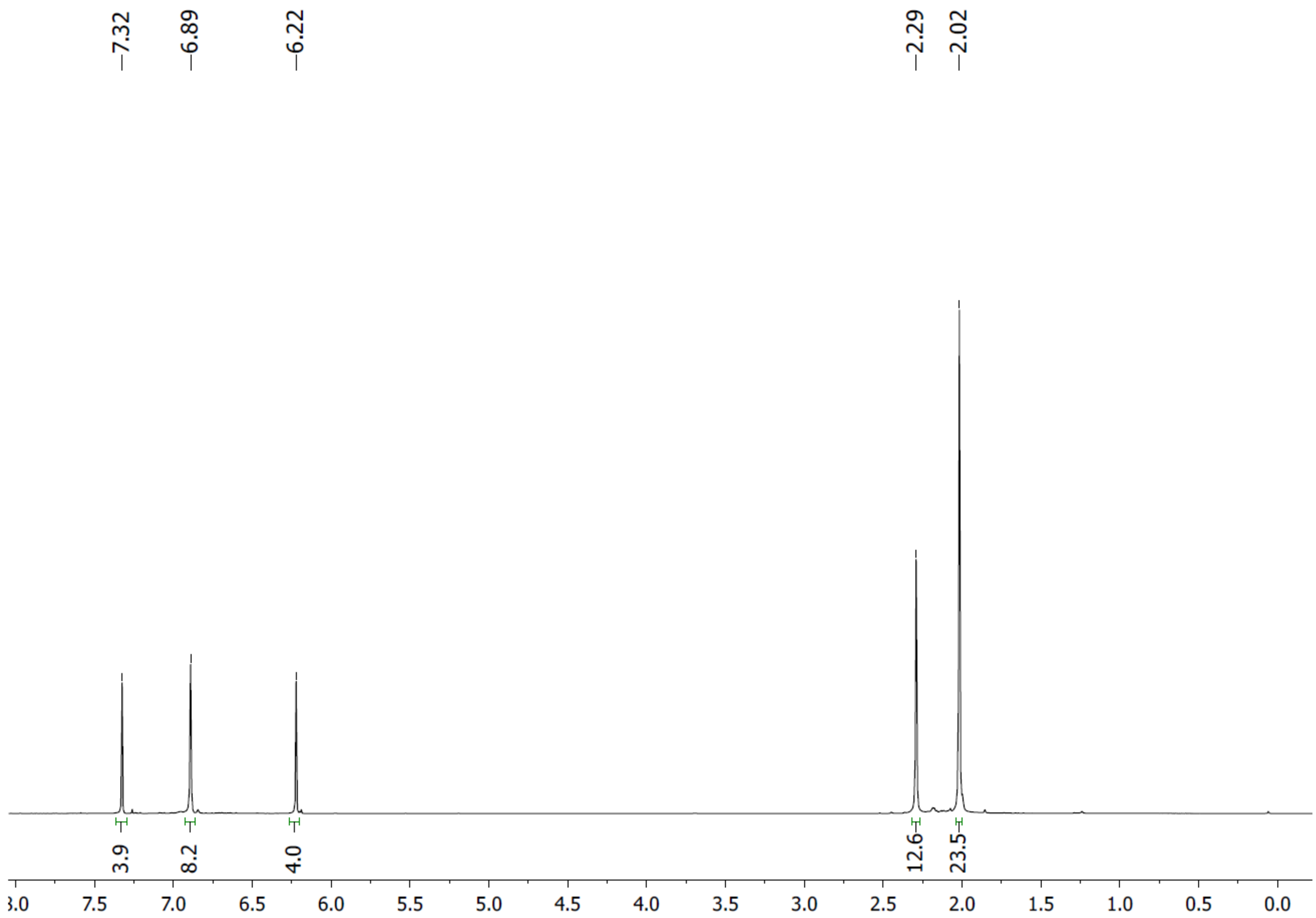

Figure S5. ^{1}H NMR spectrum (400.1 MHz, $CDCl_3$) of **6**.

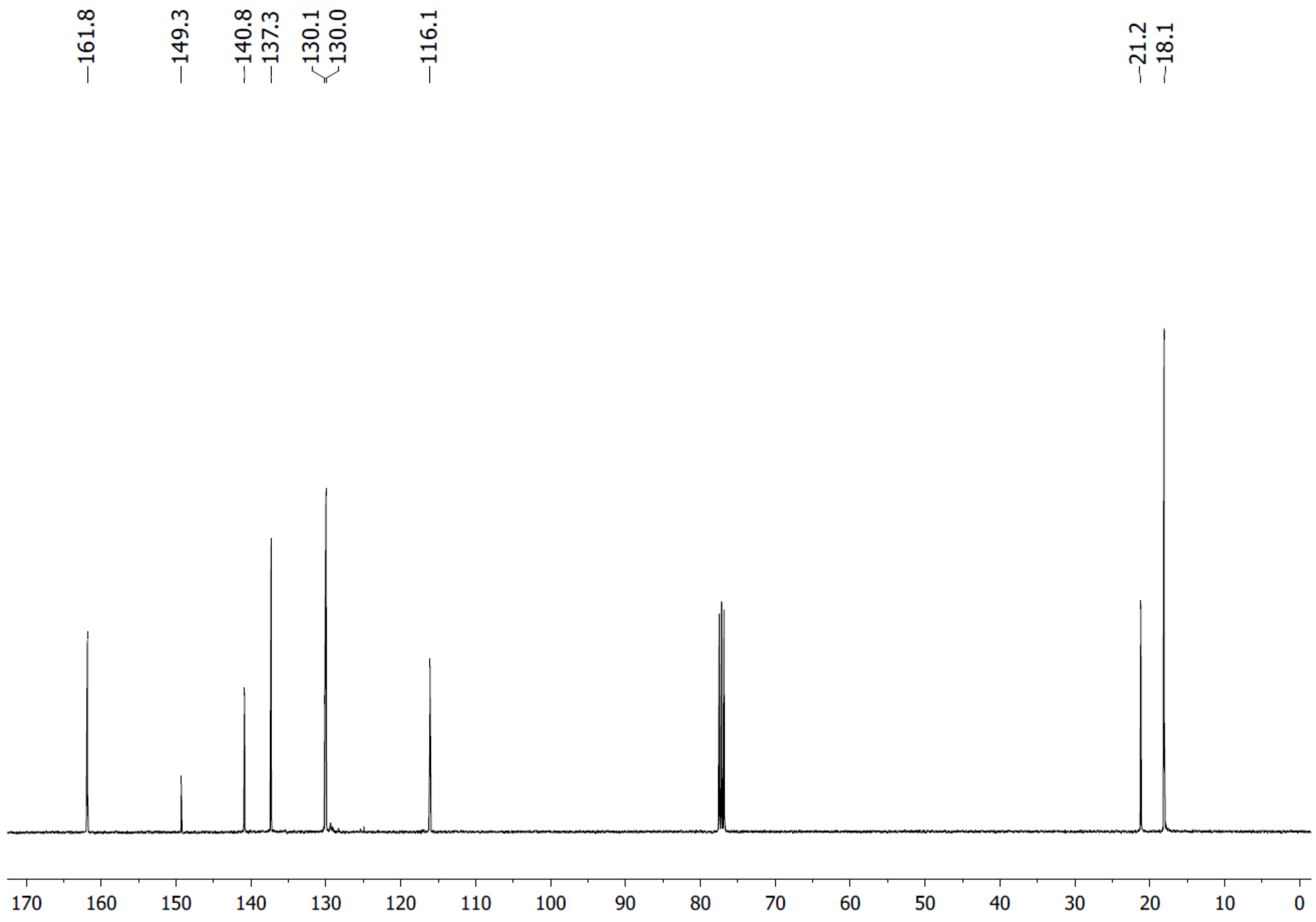

Figure S6. $^{13}C\{^{1}H\}$ NMR spectrum (100.6 MHz, $CDCl_3$) of **6**.

Procedure for the Isolation of $[(I^{Dip}NAlH)_2HCO_2]^+[HCO_2(B(C_6F_5)_3)_2]^-$ ($7^+[HCO_2(B(C_6F_5)_3)_2]^-$)

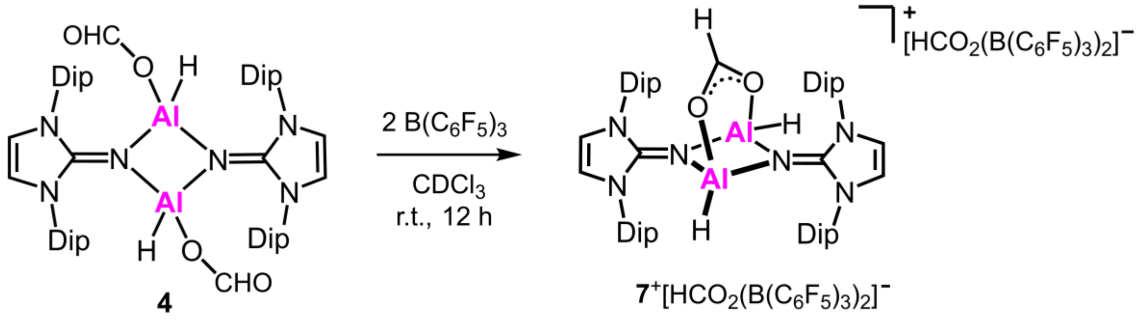

A 25 mL Schlenk flask was charged with 234 mg of **2** (0.26 mmol) and 253 mg BCF (0.49 mmol, BCF = tris(pentafluorophenyl)borane). The mixture of solids was cooled to 0 °C and 3.9 mL $CDCl_3$ were added by syringe. The reaction mixture was stirred for 30 min at 0 °C, the cooling was removed and it was stirred over night at room temperature. An oil separated upon addition of pentane to the reaction mixture. Separation of the phases and removal of volatiles from the oil leads to formation of an off-white foam that is crushed into a fine powder using a spatulum.

^{1}**H NMR** (400.1 MHz, $CDCl_3$): δ = 8.18 (s, 1H, BCF-O_2CH), 7.69 (s, 1H, Al-O_2CH), 7.38 (t, 4H, J = 7.8 Hz, DipH-4), 7.13 (d, 8H, J = 7.8 Hz, DipH-3,5), 6.45 (s, 4H, NCH), 2.36 (sept, 8H, J = 6.7 Hz, Dip-CH($CH_3)_2$), 1.12 (d, 24H, J = 6.8 Hz, Dip-CH(C$H_3)_2$), 1.08 (d, 24H, J = 6.8 Hz, Dip-CH(C$H_3)_2$). ^{11}**B NMR** (128.4 MHz, $CDCl_3$): δ = −0.7. ^{13}**C{^{1}H} NMR** (100.6 MHz, $CDCl_3$): δ = 173.6 (O_2CH), 172.4 (O_2CH), 150.1 (NCN), 146.9 (DipC-1), 131.7 (DipC-2,6), 130.8 (DipC-4), 125.4 (DipC-3,5), 116.7 (NCH), 29.0 (CH($CH_3)_2$), 25.1 (CH($CH_3)_2$), 22.5 (CH($CH_3)_2$), n.a. (CF).

Elemental analysis: calcd (%) for $\mathbf{7}^+[HCO_2(B(C_6F_5)_3)_2]^-$ [1975.19]: C 55.94, H 3.88, N 4.25; found: C 55.19, H 3.76, N 4.19. The low value found for carbon is explained by the formation of incombustible boron carbides.

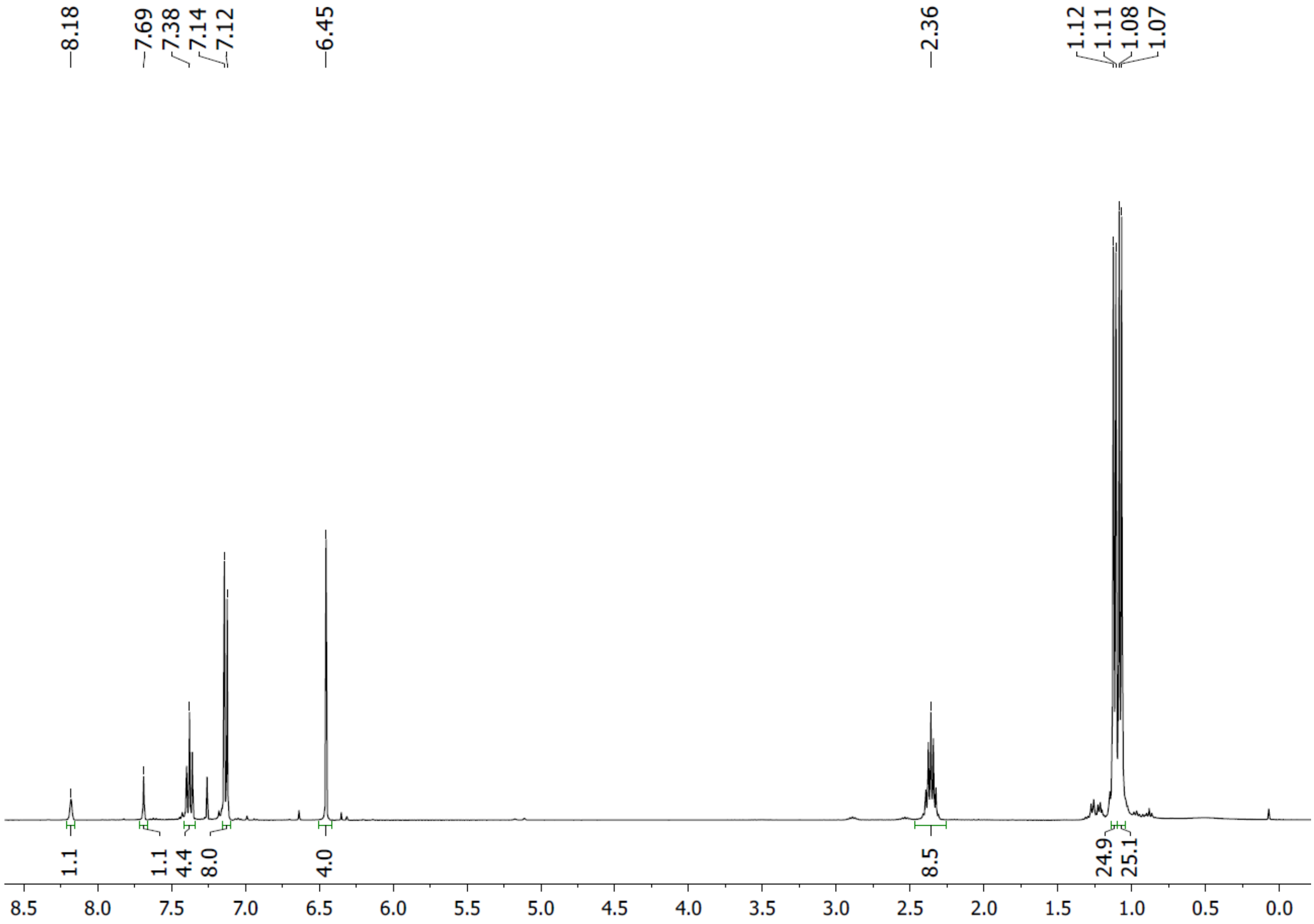

Figure S7. ^{1}H NMR spectrum (400.1 MHz, $CDCl_3$) of crude **7**$^{+}$[$HCO_2(B(C_6F_5)_3)_2$]$^{-}$.

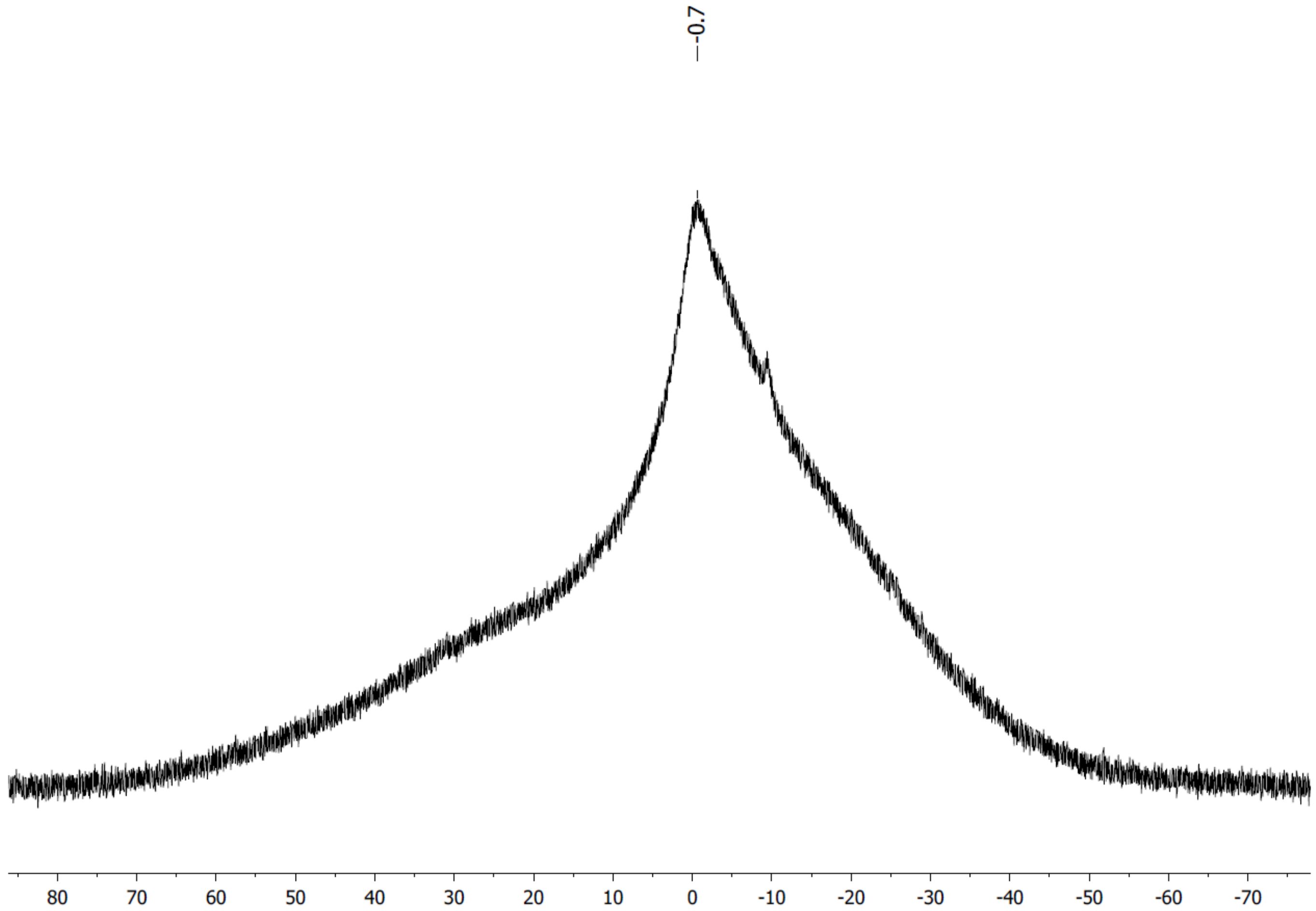

Figure S8. ^{11}B NMR spectrum (128.4 MHz, $CDCl_3$) of crude **7**$^+[HCO_2(B(C_6F_5)_3)_2]^-$.

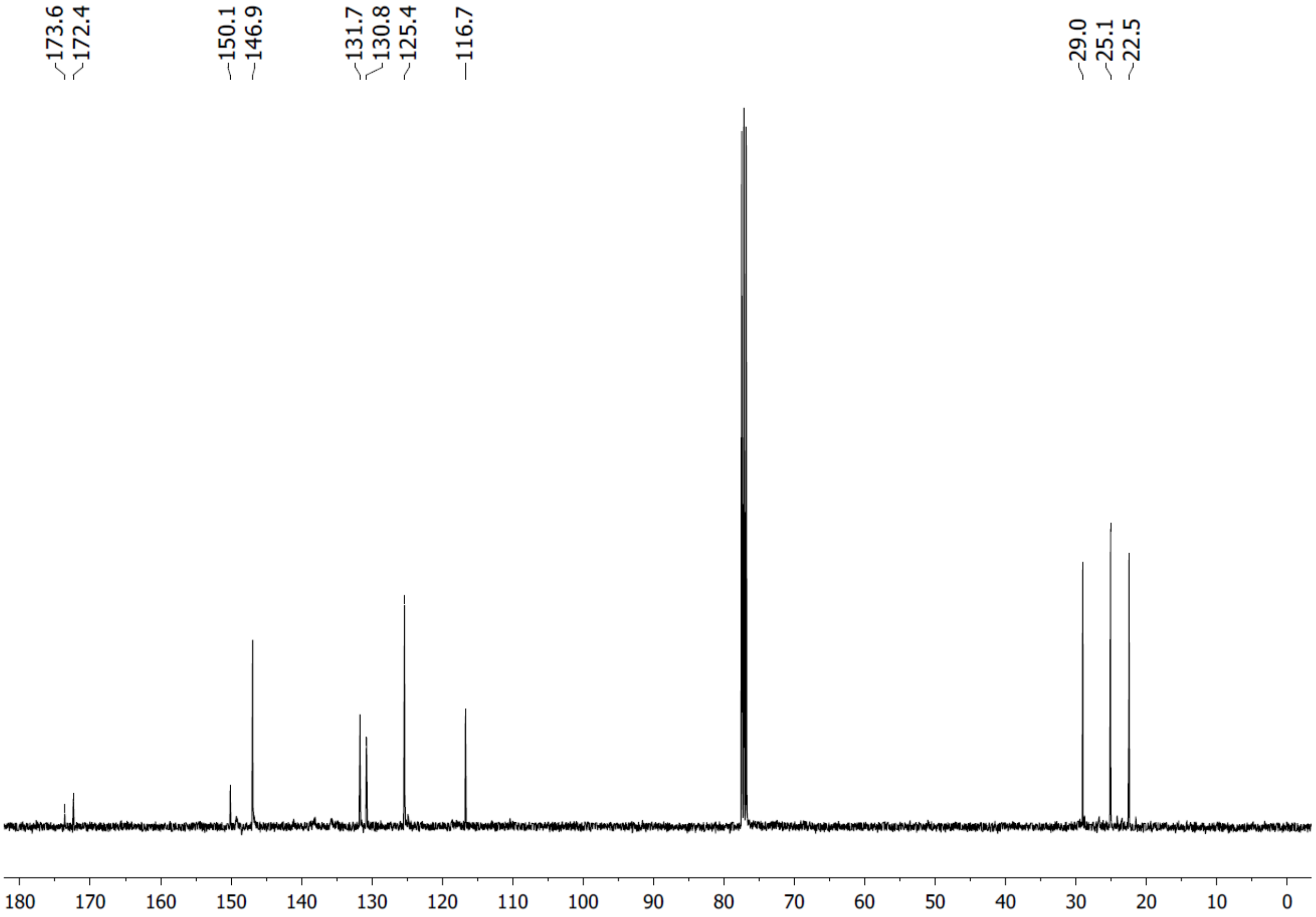

Figure S9. $^{13}C\{^{1}H\}$ NMR spectrum (100.6 MHz, $CDCl_3$) of **7**$^{+}[HCO_2(B(C_6F_5)_3)_2]^{-}$.

Procedure for the Crystallization of $[(I^{Dip}NAlH)_2HCO_2]^+[Al(OC(CF_3)_3)_4]^-$ ($7^+[Al(OC(CF_3)_3)_4]^-$)

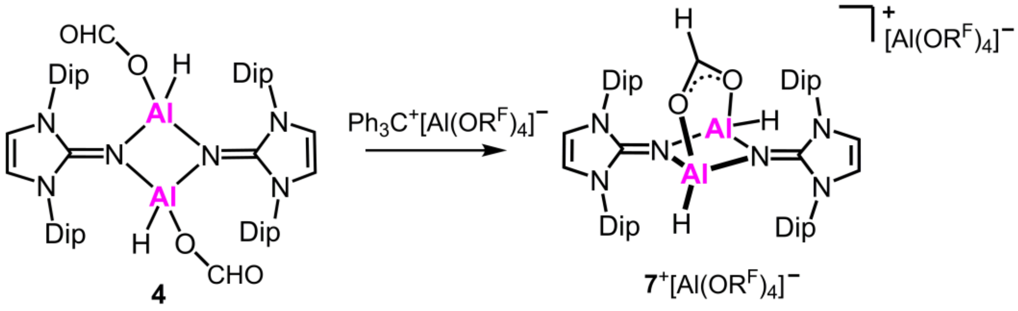

Crude **4** (55 mg, 0.06 mmol) and $Ph_3C^+[Al(OC(CF_3)_3)_4]^-$ were mixed in 1 mL toluene and stirred for 24 h. The resulting suspension was mixed with pentane (1.5 mL). The solid was sedimented and the supernatant withdrawn. The solid was dissolved in 0.7 mL 1,2-difluorobenzene and the vial containing this solution was placed in a reservoir with a mixture of toluene/pentane (2 mL / 4 mL) for gas phase diffusion exchange of solvent. After three days the vial was separated from the reservoir and the solvent slowly allowed to evaporate in a glovebox workstation atmosphere to afford crystals suitable for SCXRD analysis.

Procedure for the synthesis of crude $(I^{Dip}NCH_2)_2{\cdot}(HOTs)_2$ (8·$(HOTs)_2$)

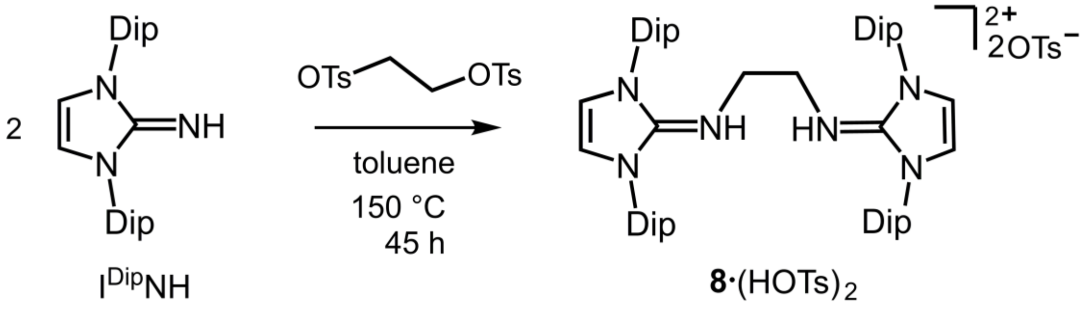

The compound was synthesized and isolated similar to a procedure for $(L^{Mes}NCH_2)_2{\cdot}(HOTs)_2$[S4]: A Schlenk vessel equipped with a *J. Young's* PTFE tap and a magnetic stirrer bar was charged with $L^{Dip}NH$ (4.456 g, 11.04 mmol) and 1,2-bistosylethane (2.05 g, 5.5 mmol). To the stirring mixture of solids was added toluene (35 mL) and the pressure within the vessel was reduced (a negligible amount of toluene evaporated or it was cooled to prevent solvent loss). The tap was closed to seal the reaction vessel before placing it into an oil bath heated to 150 °C (the top level of the oil should be a little above the surface level of the reaction mixture), and the reaction was driven for 45 h. The solid was collected on a frit and washed with 20 mL toluene before compressing the filter cake by applying a pressure gradient along the frit. The powder was dried in vacuum after which 5.683 g (4.83 mmol, 88%) of crude product were transfered from the frit.

^{1}H NMR (400.1 MHz, $CDCl_3$): δ = 8.22 (br, 2H, N*H*), 7.46 (t, *J* = 8 Hz, 4H, DipH-4), 7.38 (d, *J* = 8 Hz, 4H, TosH-2,6 or -3,5), 7.20 (d, *J* = 8 Hz, 8H, DipH-3,5), 6.97 (d, *J* = 8 Hz, 4H, TosH-2,6 or -3,5), 6.85 (s, 4H, NC*H*), 2.80 (br, 4H, C_2H_4), 2.45 (sept, *J* = 7 Hz, 8H, Dip-C*H*$(CH_3)_2$), 2.27 (s, 6H, Tos-CH_3), 1.10 (d, *J* = 7 Hz, 24H, Dip-CH(CH_3)$_2$), 0.98 (d, *J* = 7 Hz, 24H, Dip-CH(CH_3)$_2$). **^{13}C{^{1}H} NMR** (100.6 MHz, $CDCl_3$): δ = 145.9 (ArC), 144.8 (ArC), 143.6 (ArC), 138.5 (ArC), 132.0 (ArC), 129.2 (ArC), 128.0 (ArC), 126.3 (ArC), 125.3 (ArC), 119.8 (N*C*H), 42.5 (C_2H_4), 29.1 (*C*H$(CH_3)_2$), 24.3 (CH(*C*H_3)$_2$), 23.1 (CH(*C*H_3)$_2$), 21.4 (Tos-*C*H_3).

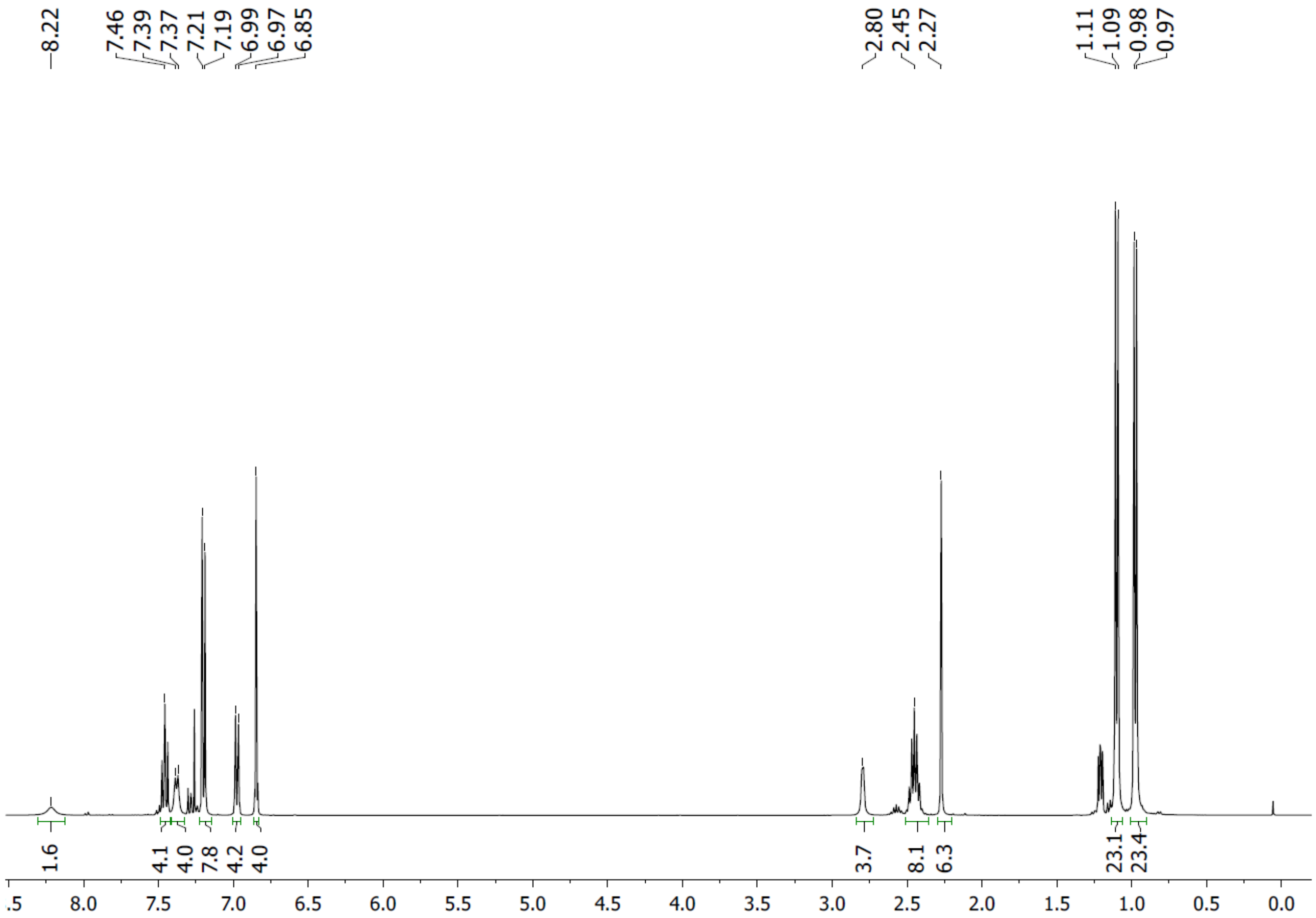

Figure S10. ^{1}H NMR spectrum (400.1 MHz, $CDCl_3$) of crude **8**·$(HOTs)_2$.

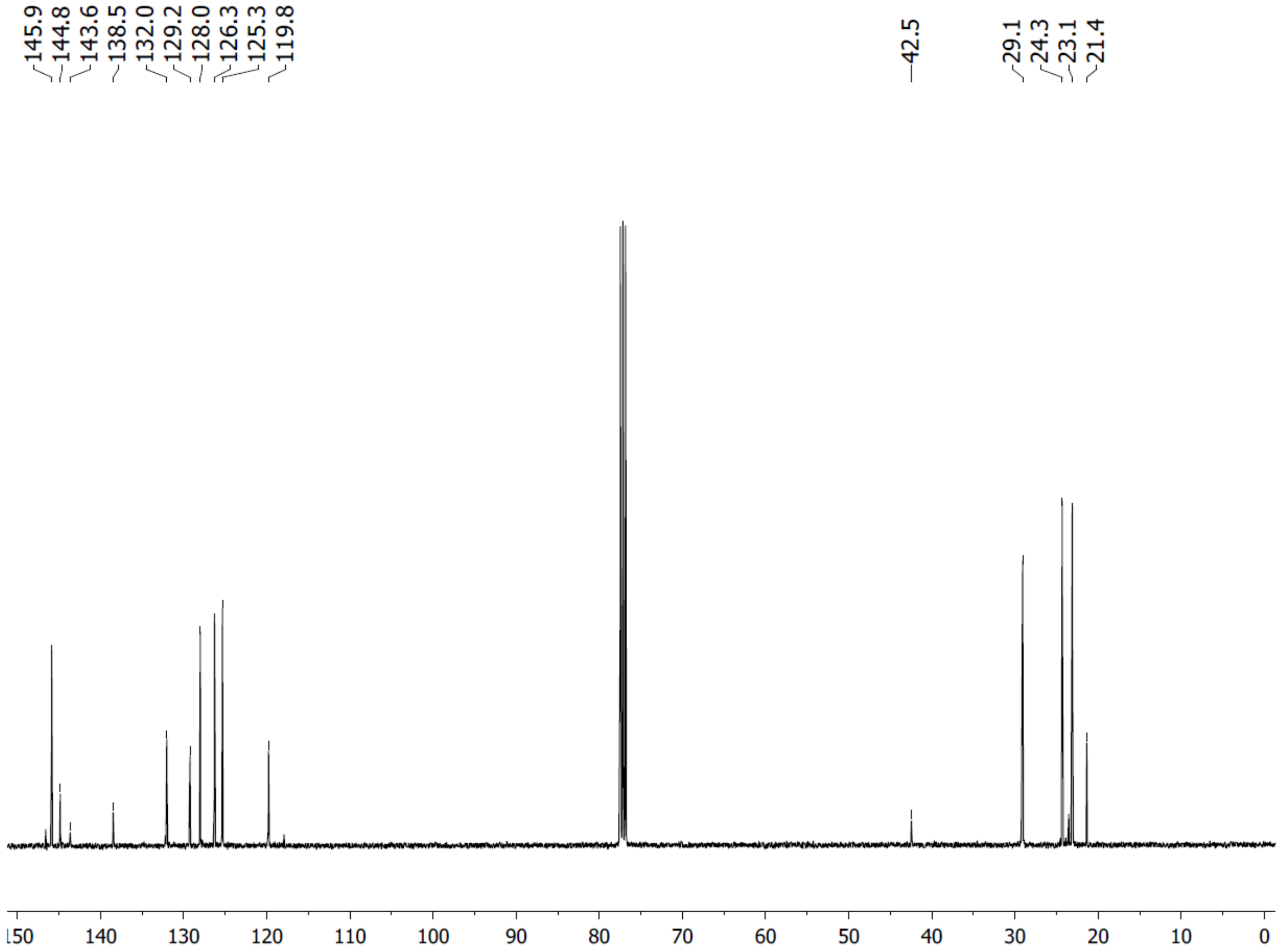

Figure S11. $^{13}C\{^1H\}$ NMR spectrum (100.6 MHz, $CDCl_3$) of crude **8**·$(HOTs)_2$.

Procedure for the Isolation of [(I^{Dip}NCH$_2$)$_2$AlH$_2$]$^+$[OTs]$^-$ (9$^+$[OTs]$^-$)

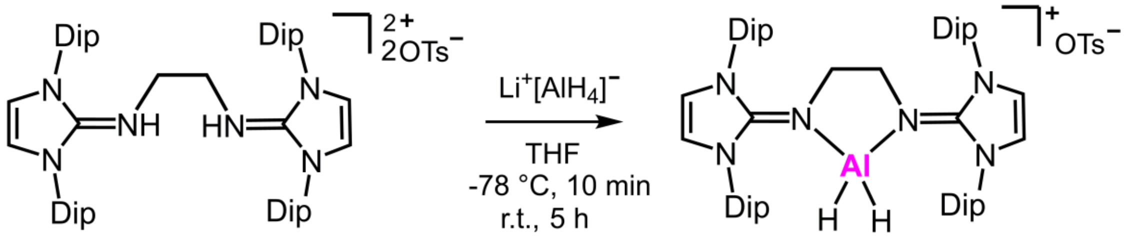

8·(HOTs)$_2$ **9**$^+$[OTs]$^-$

To a suspension of **8**·(HOTs)$_2$ (1.165 g, 0.99 mmol) in 4 mL THF was carefully added a solution (1 molar) of Li[AlH$_4$] in THF (0.89 g ≡ 0.98 mL, 0.98 mmol) at −78 °C with stirring. The reaction was continued for 10 min at low temperature then the cooling was removed and it was stirred for 5 h at room temperature *(beware: evervescence!)*. A slightly turbid solution was obtained and the volatiles were evaporated under reduced pressure (30 min). The residue was dried in dynamic vaccum for 3 h to yield a foamy solid. The product was extracted into 4.5 mL CDCl$_3$ and mixing the filtrate with 6 mL pentane resulted in phase separation (liquid/liquid, lower phase with smaller volume). After storage at −30 °C over night no solid had formed. However, upon briefly keeping the biphasic mixture at room temperature few crystal seeds spontanously formed. Crystal growth was continued at −30 °C for one day to yield a major solid fraction. The cold supernatant was decanted and the solid fraction was homogenized by manipulation with a spatulum before it was extensively dried in dynamic vacuum for one day. From the glass vessel 649 mg of a coloreless powder were transfered that analyzed to **9**$^+$[OTs]$^-$ with no lattice solvent included (0.63 mmol, 64%).
Crystals suitable for SCXRD analysis were grown from a mixture of THF, Et$_2$O, pentane and 1,2-difluorobenzene at −30 °C over a period of 10 days.

^{1}H NMR (300.1 MHz, CDCl$_3$): δ = 7.84 (d, *J* = 8 Hz, 2H, TosH-2,6 or -3,5), 7.42 (t, *J* = 8 Hz, 4H, DipH-4), 7.14 (d, *J* = 8 Hz, 8H, DipH-3,5), 7.00 (d, *J* = 8 Hz, 2H, TosH-2,6 or -3,5), 6.50 (s, 4H, NC*H*), 2.53 (sept, *J* = 7 Hz, 8H, Dip-C*H*(CH$_3$)$_2$), 2.36 (s, 4H, C$_2$*H*$_4$), 2.24 (s, 3H, Tos-C*H*$_3$), 1.77 (br, 2H, Al*H*), 1.10 (d, *J* = 7 Hz, 24H, Dip-CH(C*H*$_3$)$_2$), 1.08 (d, *J* = 7 Hz, 24H, Dip-CH(C*H*$_3$)$_2$). **^{13}C{^{1}H} NMR** (100.6 MHz, CDCl$_3$): δ = 149.0 (ArC), 146.8 (ArC), 146.5 and 145.3 (ArC), 137.7 (ArC), 131.8 (ArC), 131.0 (ArC), 128.1 (ArC), 126.6 (ArC), 124.7 (ArC), 118.7 (N*C*H), 46.0 (*C*$_2$H$_4$), 29.1 (*C*H(CH$_3$)$_2$), 25.2 (CH(*C*H$_3$)$_2$), 22.3 (CH(*C*H$_3$)$_2$), 21.4 (Tos-*C*H$_3$).
Elemental analysis: calcd (%) for **9**$^+$[OTs]$^-$ [1033.45]: C 73.22, H 8.29, N 8.13, S 3.10; found: C 72.92, H 8.46, N 8.32, S 2.80.

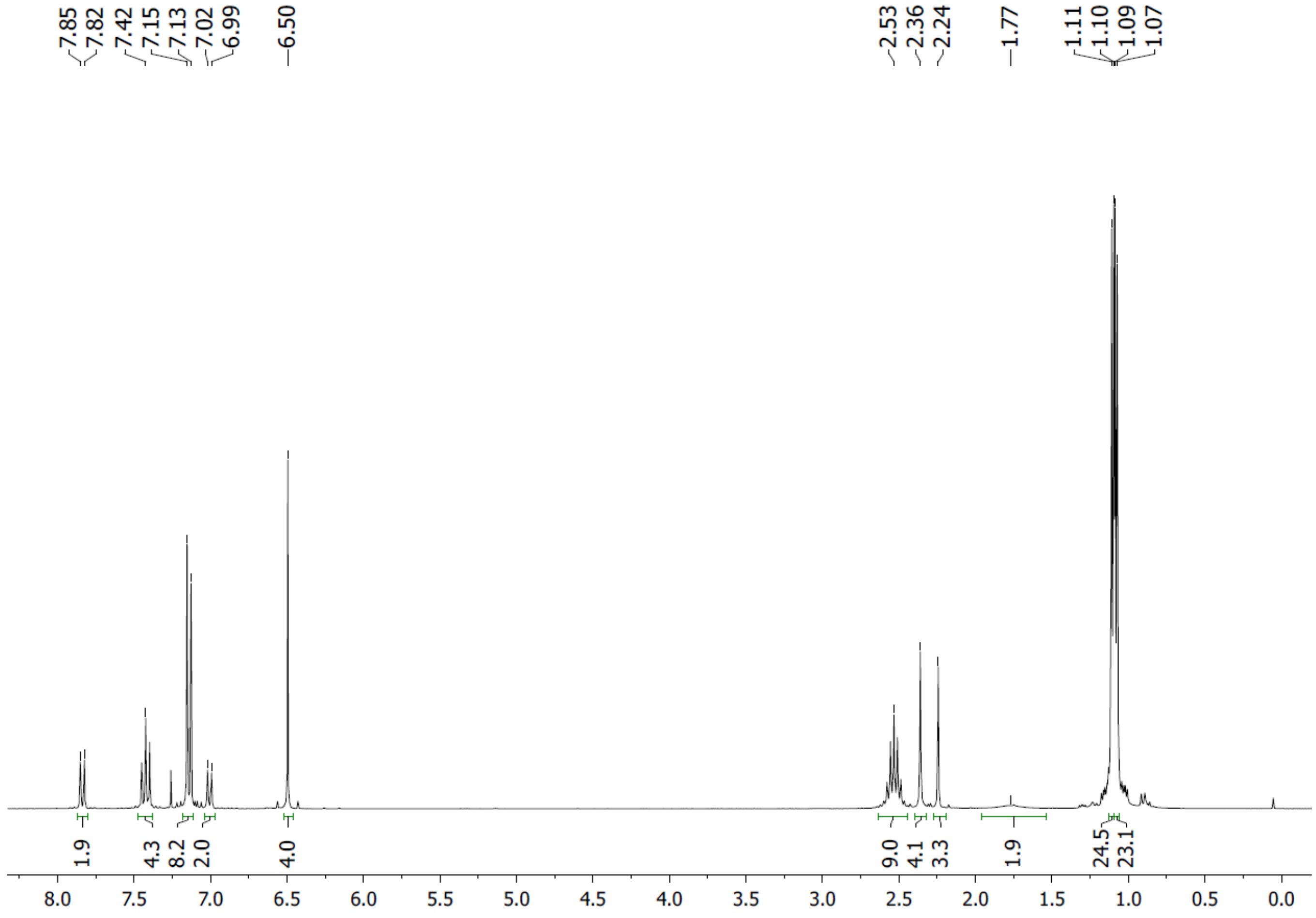

Figure S12. ^1H NMR spectrum (300.1 MHz, $CDCl_3$) of **9**$^+$[OTs]$^-$.

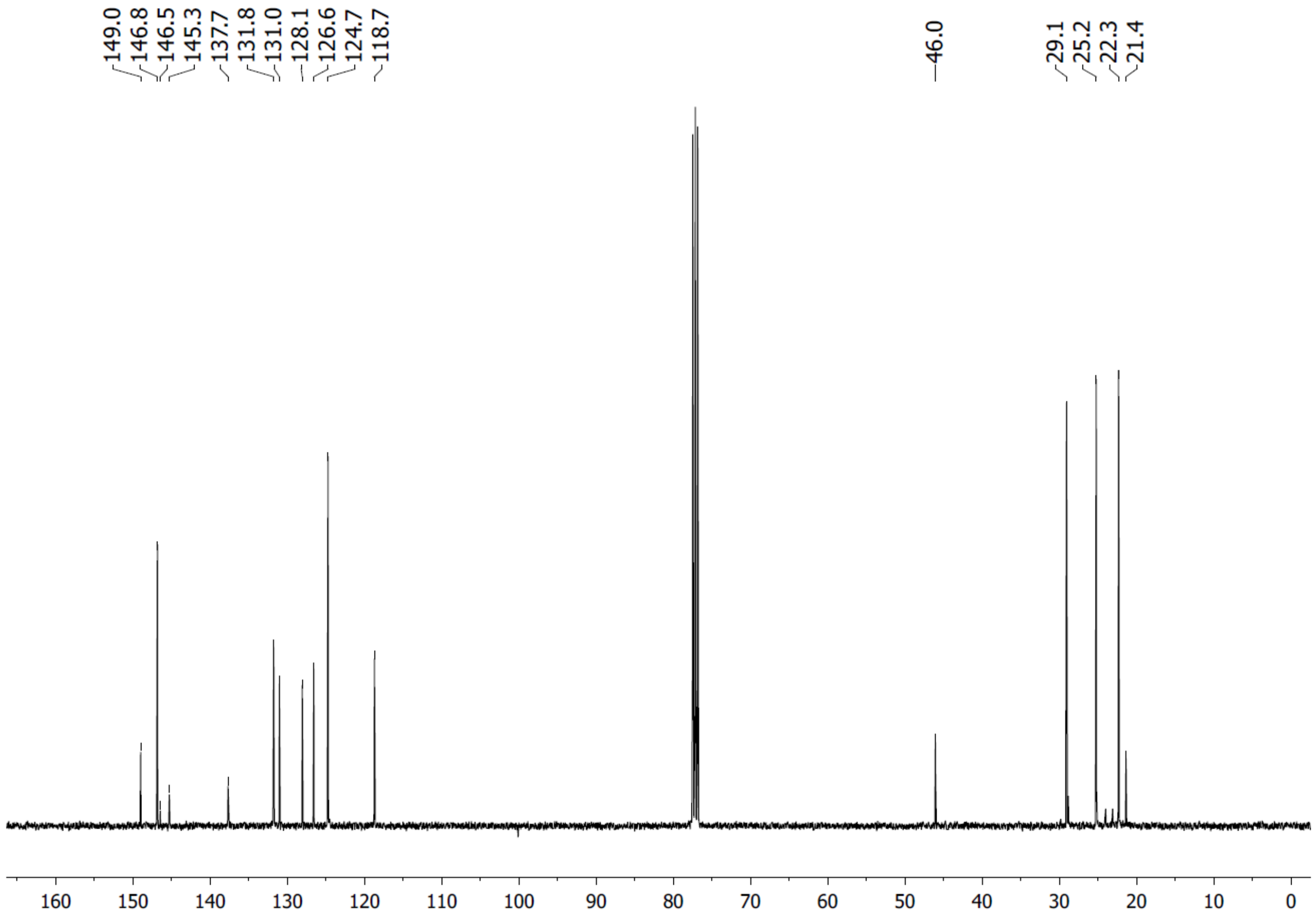

Figure S13. $^{13}C\{^{1}H\}$ NMR spectrum (100.6 MHz, $CDCl_3$) of **9**$^{+}$[OTs]$^{-}$.

Procedure for the Isolation of $[(I^{Dip}NCH_2)_2B(H)HCO_2]^+[OTs]^-$ ($\mathbf{11}^+[OTs]^-$)

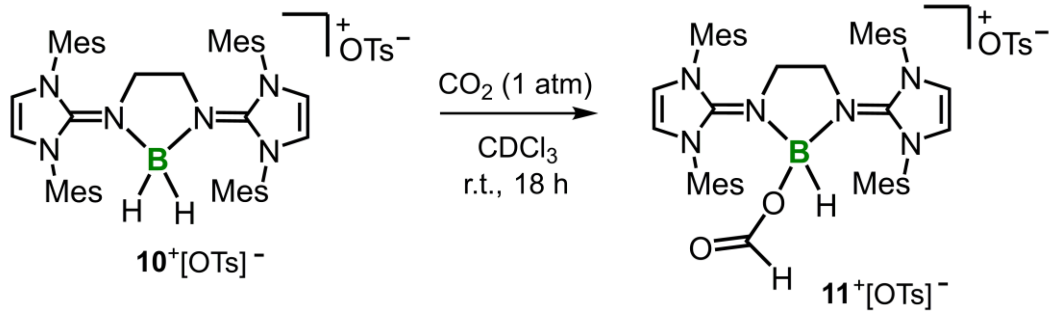

In a 50 mL Schlenk flask $\mathbf{10}^+[OTs]^-$ (377 mg, 0.44 mmol) was dissolved in $CDCl_3$ (4 mL). The solution was frozen at liquid nitrogen temperature and the vessel evacuated. While thawing in a water bath (fresh from "non-warm" tap) the flask was pressurised with CO_2 (1.0-1.1 bar) and the reaction driven for 18 h at room temperature. The solvent was evaporated *in vacuo* to yield a solid foam which was crushed to a fine powder using a spatulum. From the reaction vessel 350 mg of a colorless powder were transferred that analyzed to $\mathbf{10}^+[OTs]^-$ containing $^2/_3$ equivalent of $CDCl_3$ as lattice solvent (0.36 mmol, 82%).
Crystals suitable for SCXRD analysis were obtained from a product fraction containing stoichiometric amounts of CH_2Cl_2 dissolved in a mixture of $CDCl_3$, THF, and Et_2O (1:1:1) that had been stored at −30 °C for one day.

^{1}H NMR (400.1 MHz, $CDCl_3$): δ = 7.72 (d, J = 8 Hz, 2H, TosH-2,6 or -3,5), 6.98 (d, J = 8 Hz, 2H, TosH-2,6 or -3,5), 6.90 (s, 4H, MesH-3,5), 6.88 (s, 4H, MesH-3,5), 6.61 (s, 4H, NC*H*), 5.51 (s, 1H, HCO_2), 2.76 (m, 2H, C_2H_4), 2.47 (m, 2H, C_2H_4), 2.34 (s, 12H, MesCH_3-4), 2.24 (s, 3H, Tos-CH_3), 2.00 (s, 12H, MesCH_3-2,6), 1.97 (s, 12H, MesCH_3-2,6), n.a. B*H*. **^{11}B NMR** (128.4 MHz, $CDCl_3$): δ = −1.2. **^{13}C{^{1}H} NMR** (100.6 MHz, $CDCl_3$): δ = 163.8 (HCO_2), 145.2 (ArC), 144.9 (ArC), 140.3 (ArC), 137.8 (ArC), 135.5 and 135.2 (ArC), 132.0 (ArC), 129.8 and 129.5 (ArC), 128.0 (ArC), 126.2 (ArC), 119.1 (N*C*H), 45.5 (C_2H_4), 21.2 (Tos-CH_3), 21.1 (MesCH_3-4), 17.9 (MesCH_3-2,6), 17.6 (MesCH_3-2,6).
Elemental analysis: calcd (%) for $\mathbf{10}^+[OTs]^-\cdot(CDCl_3)_{2/3}$ [973.22]: C 65.00, H 6.46, N 8.64, S 3.29; found: C 64.57, H 6.47, N 8.71, S 3.12.

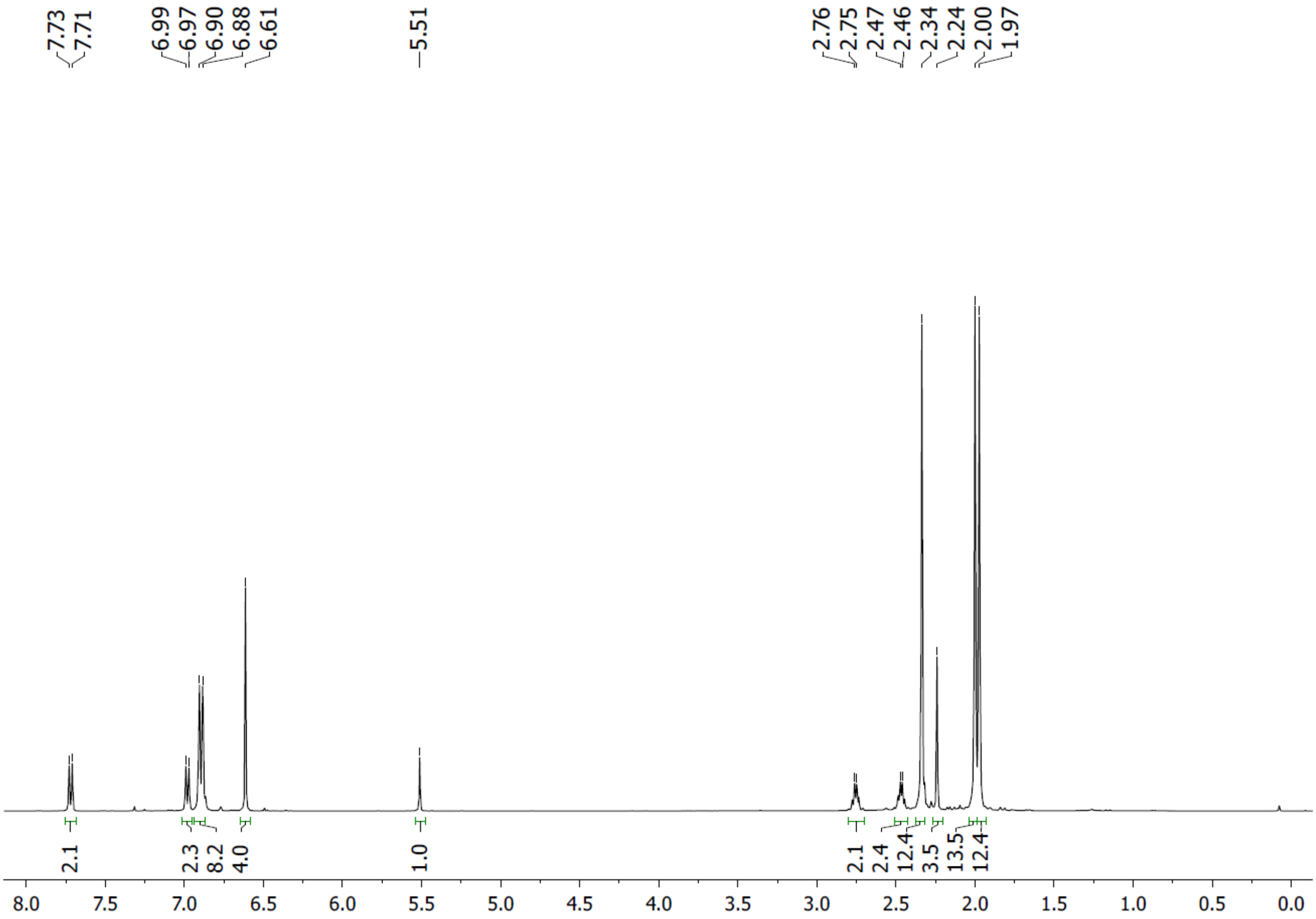

Figure S14. ^{1}H NMR spectrum (400.1 MHz, $CDCl_3$) of **11**$^{+}$[OTs]$^{-}$.

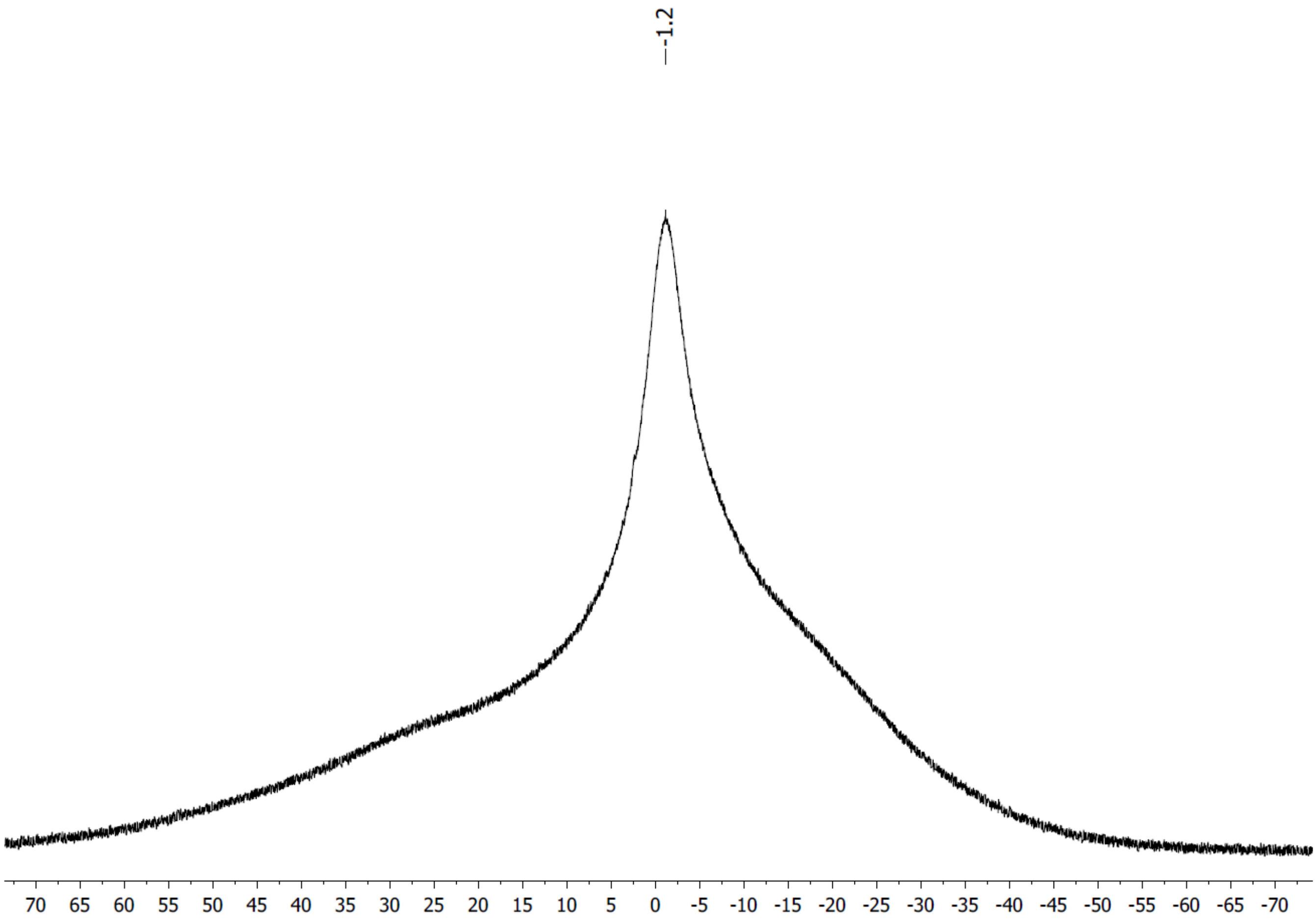

Figure S15. ^{11}B NMR spectrum (128.4 MHz, $CDCl_3$) of **11**$^{+}$[OTs]$^{-}$ (note: the half-width of the signal was not determined as it is difficult to distinguish from the broad signal produced by the sample tube and NMR apparatus).

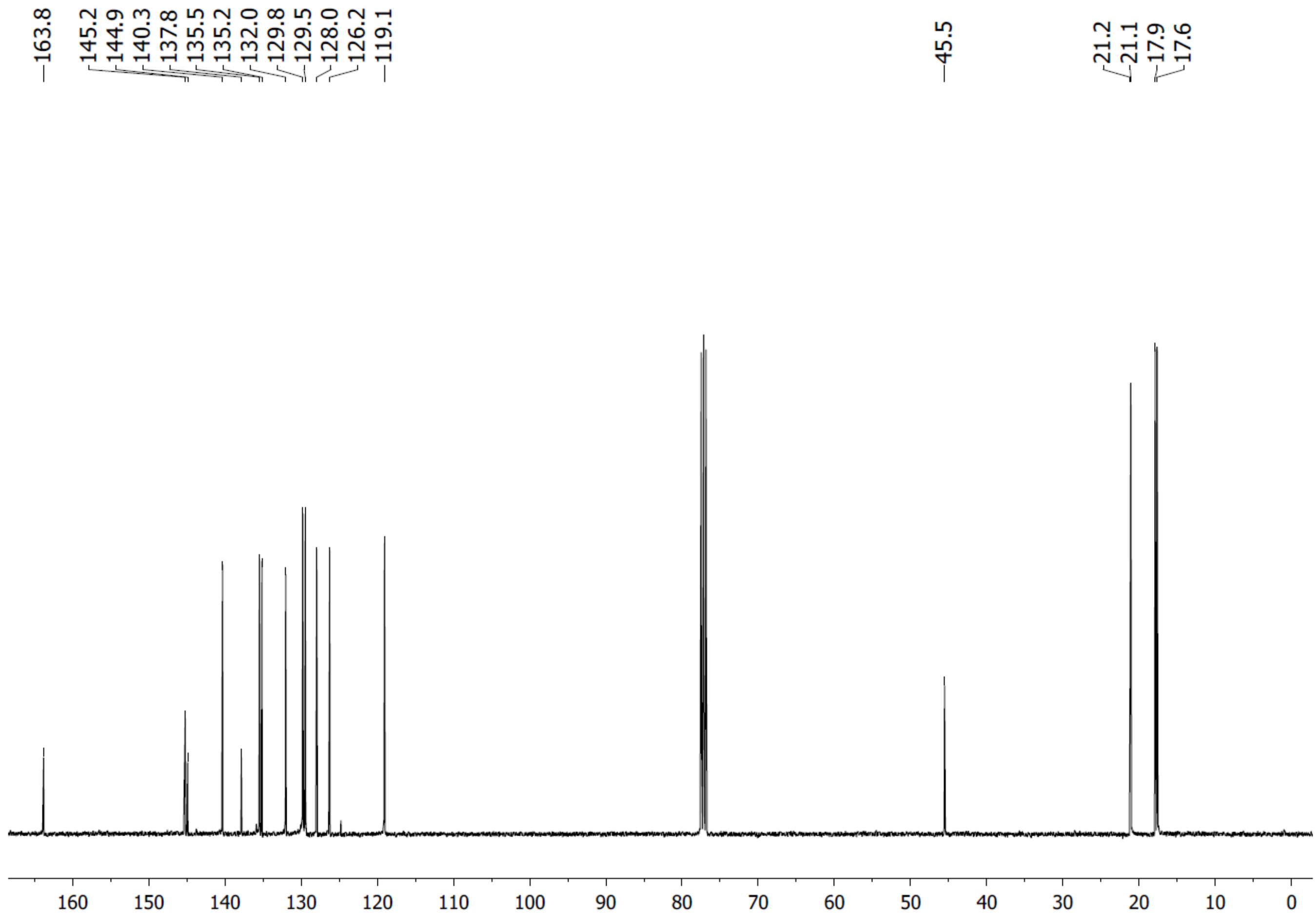

Figure S16. $^{13}C\{^{1}H\}$ NMR spectrum (100.6 MHz, $CDCl_3$) of **11**$^+$[OTs]$^-$.

2.) Experimental Details – Catalysis Study

General procedure for the catalytic CO_2 reduction with borane dimethylsulfide complex

In a glovebox workstation a Schlenk flask was charged with the (pre)catalyst, the naphthalene standard (only in case of **9**$^+$[OTs]$^-$) and $CDCl_3$ (the conversions were driven on a 2-4 mL scale with regard to the reaction-mixture volume, the relative amounts can be taken from Table 1 of the main article). At the Schlenk line borane dimethylsulfide complex was added and the resulting mixture frozen at liquid nitrogen temperature before setting the flask to vacuum. The liquid nitrogen bath was removed, the flask was pressurized with CO_2 (1.0-1.1 bar) and a water bath (fresh from "non-warm" tap) was applied for controlled thawing of the mixture with stirring. After temperature accomodation the water bath was removed and the reaction driven at room temperature with constant CO_2 pressure.

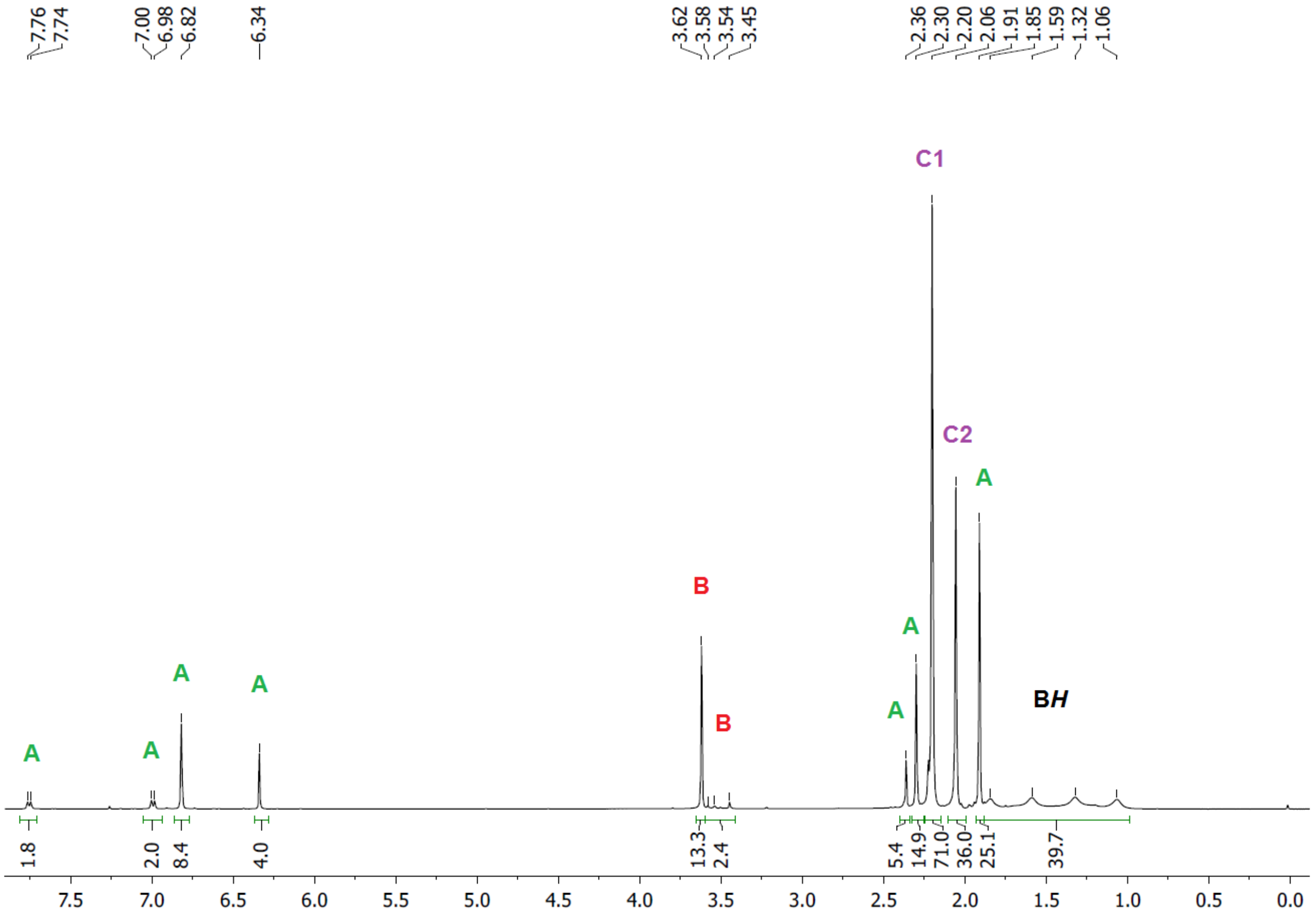

Figure S17. ^{1}H NMR spectrum (400.1 MHz, $CDCl_3$) of the conversion of $Me_2S{\cdot}BH_3$ in CO_2 atmosphere using **10**$^+$[OTs]$^-$ as a (pre)catalyst after 1 h reaction time. **A**: signals of bis(NHI) and tosylate; **B**: signals of methoxyborane species (different aggregation types of "$H_3COB(O)$" explain multiple signals); **C1**: Me_2S in $Me_2S{\cdot}BH_3$; **C2**: Me_2S; **B*H***: quartet with four lines of equal intensity produced by hydrogen atoms in $Me_2S{\cdot}BH_3$ (note: spin of ^{11}B nucleus = $^3/_2$).

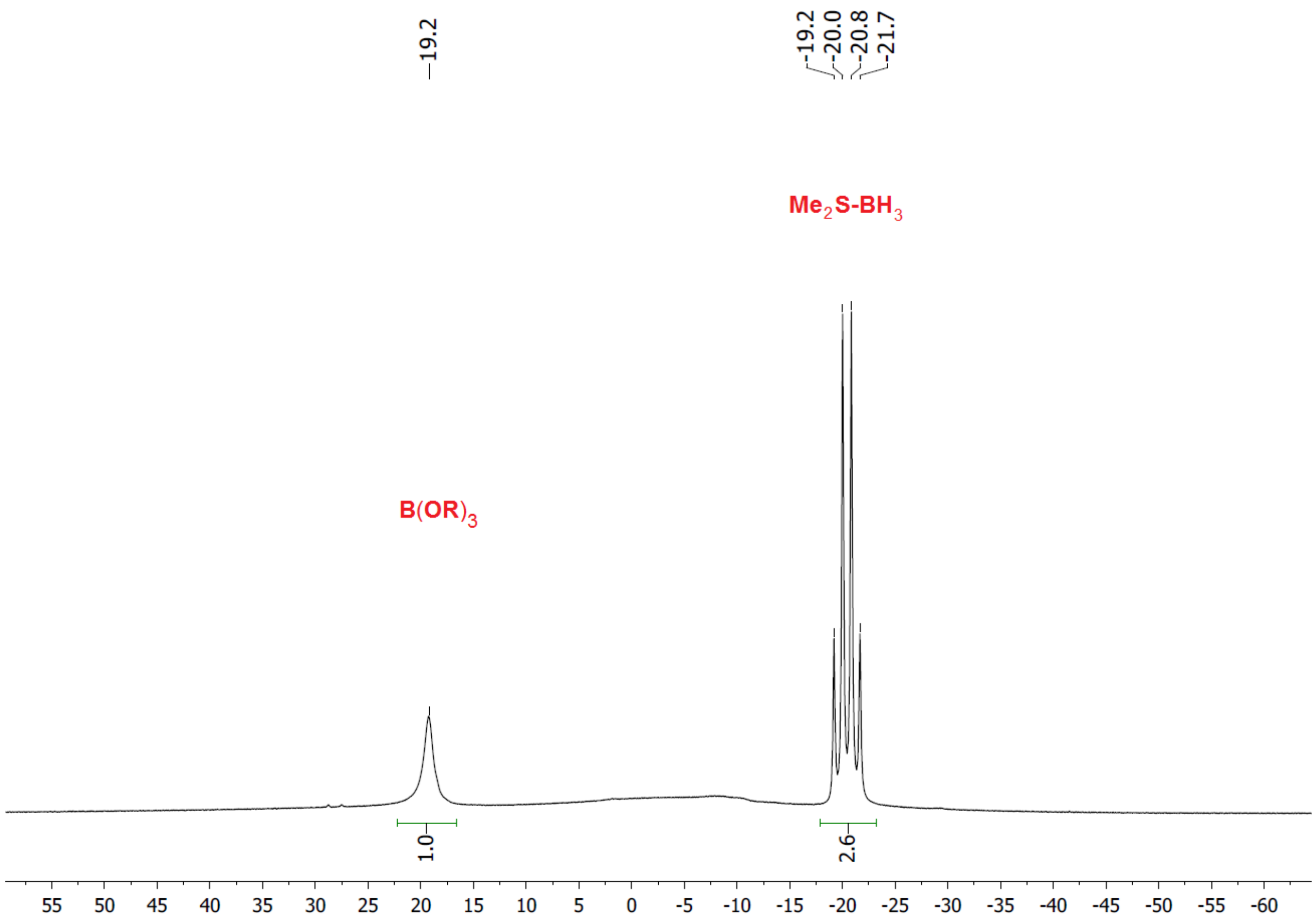

Figure S18. ^{11}B NMR spectrum (128.4 MHz, $CDCl_3$) of the conversion of $Me_2S{\cdot}BH_3$ in CO_2 atmosphere using **10**$^+$[OTs]$^-$ as a (pre)catalyst after 1 h reaction time (OR = methoxy or boroxide, baseline not corrected).

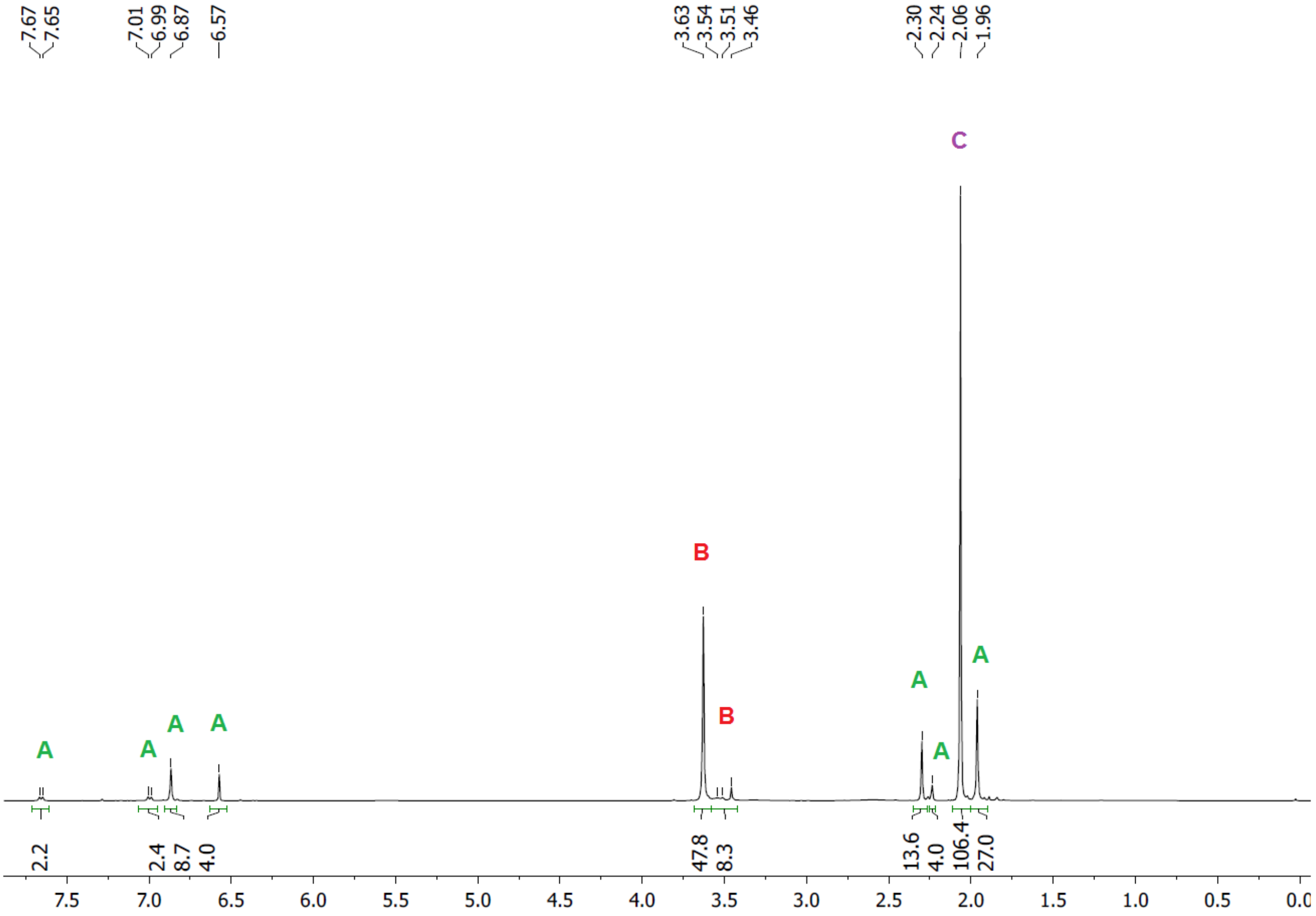

Figure S19. ^{1}H NMR spectrum (400.1 MHz, $CDCl_3$) of the conversion of $Me_2S{\cdot}BH_3$ in CO_2 atmosphere using **10**$^+$[OTs]$^-$ as a (pre)catalyst after 24 h reaction time. A: signals of bis(NHI) and tosylate; B: signals of methoxyborane species (different aggregation types of "$H_3COB(O)$" explain multiple signals); C: Me_2S.

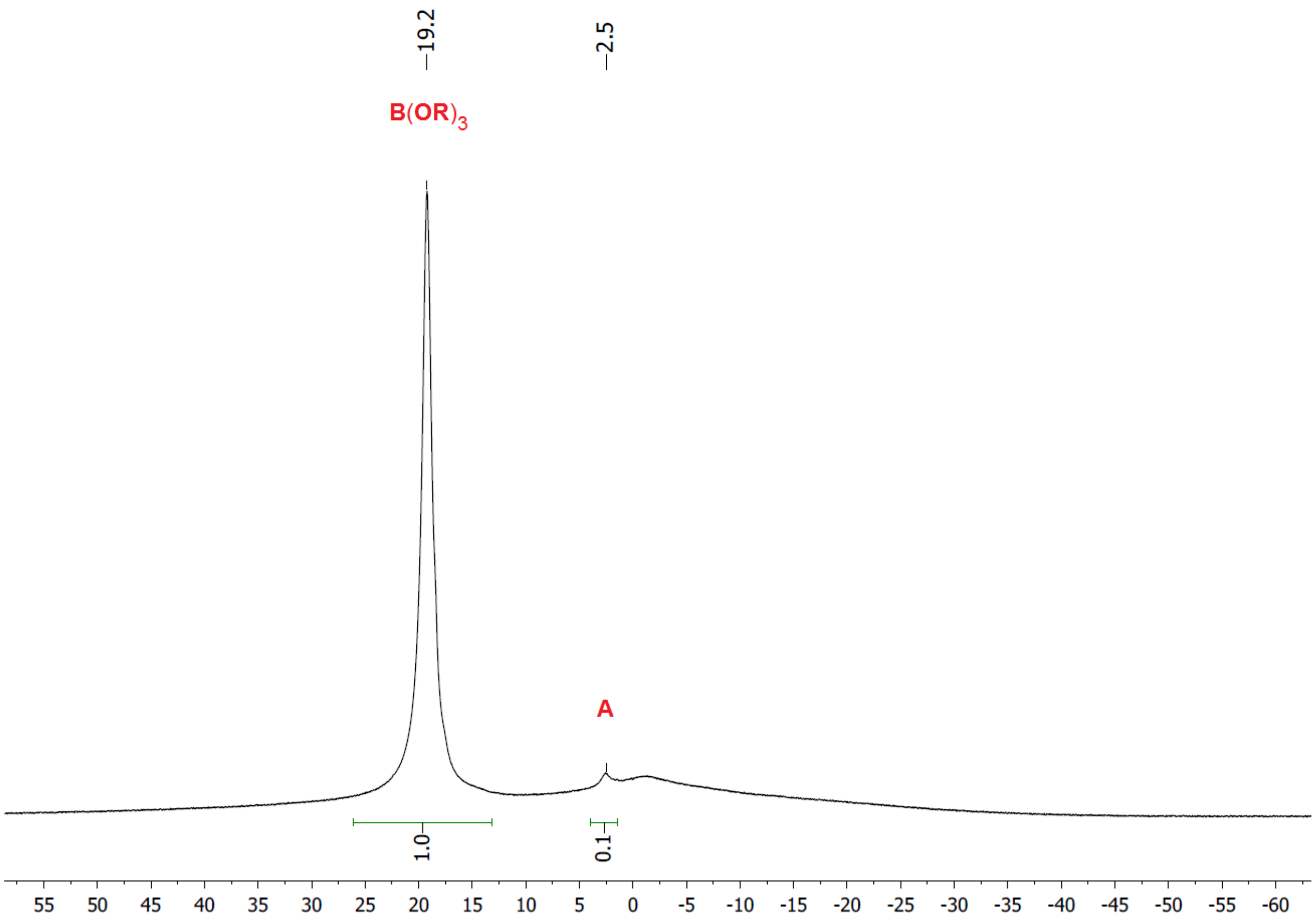

Figure S20. ^{11}B NMR spectrum (128.4 MHz, $CDCl_3$) of the conversion of $H_3B{\cdot}SMe_2$ in CO_2 atmosphere using **10**$^+$[OTs]$^-$ as a (pre)catalyst after 24 h reaction time (**OR** = methoxy or boroxide, baseline not corrected). **A**: unassigned four-coordinate boron species.

Procedure for the conversion of 4 with excess borane dimethylsulfide complex

To a solution of the biscarboxylate **4** (25 mg, 0.03 mmol) in 0.5 mL $CDCl_3$ contained in an NMR sample tube were added few droplets of $H_3B{\cdot}SMe_2$ via syringe. The tube was shaken vigorously for mixing and NMR spectra recorded after indicated elapsed time (see captions of Figures below), The relative intensities of the Me_2S signal indicated that 3 equivalents of the borane dimethylsulfide complex had been added. See main article for spectra interpretation.

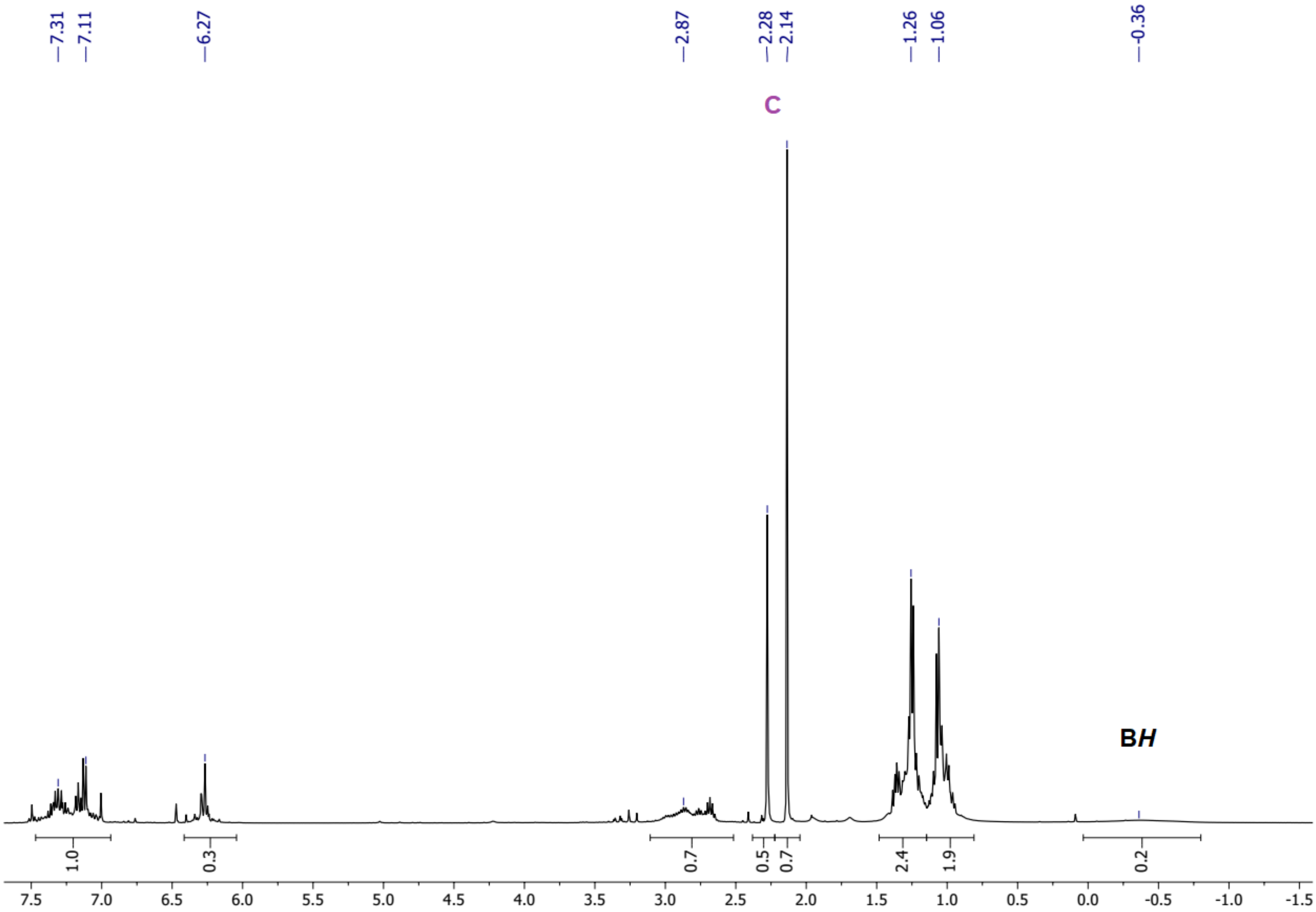

Figure S21. ^{1}H NMR spectrum (400.1 MHz, $CDCl_3$) of the conversion of **4** with approx. 3 equiv $H_3B\cdot SMe_2$ (recorded after approx. 60 min, not referenced to internal standard) showing an ill-defined mixture of species. **C**: Me_2S species.

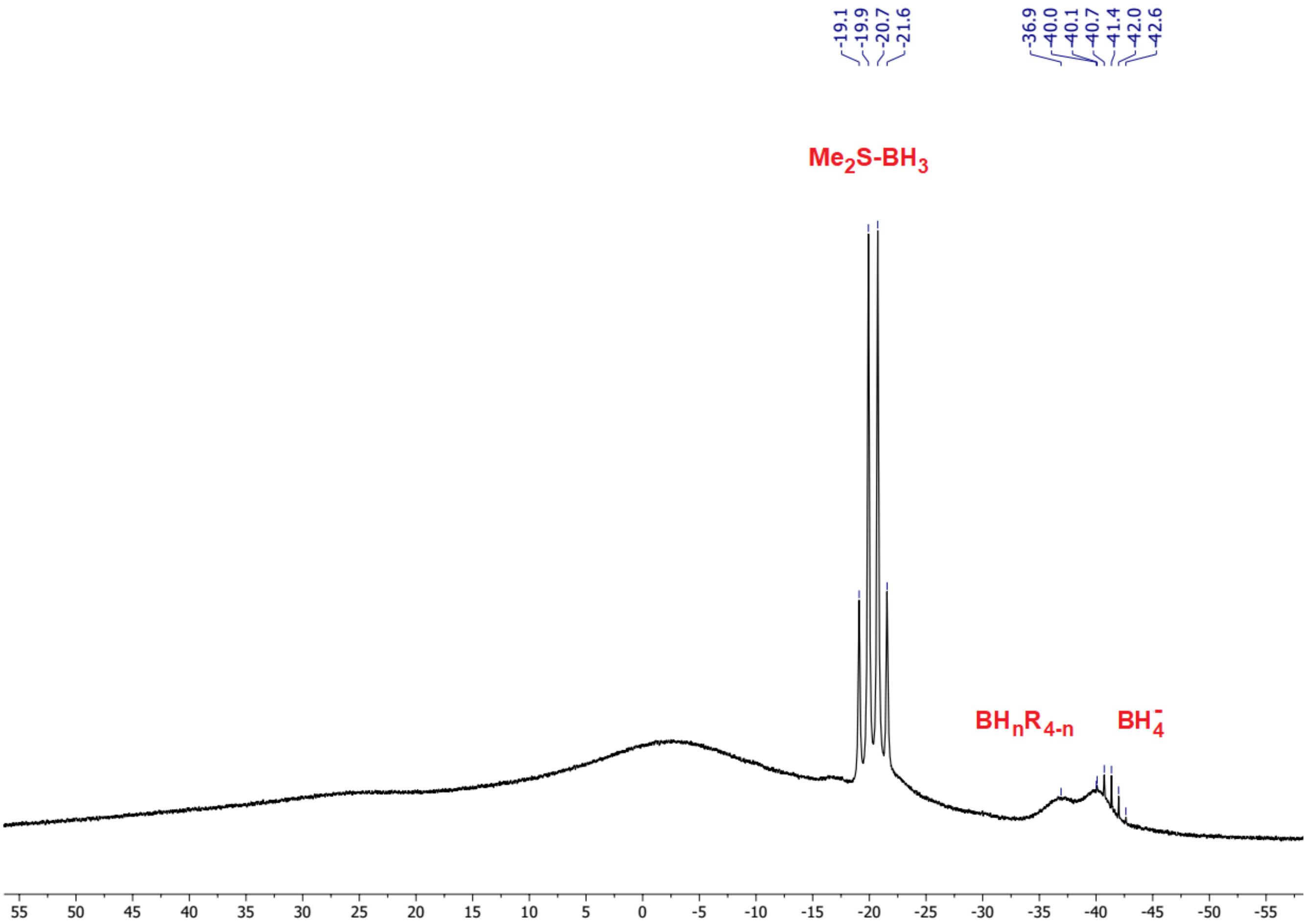

Figure S22. ^{11}B NMR spectrum (128.4 MHz, $CDCl_3$) of the conversion of **4** with approx. 3 equiv $Me_2S{\cdot}BH_3$ (recorded after approx. 1 h, see main article for additional interpretation).

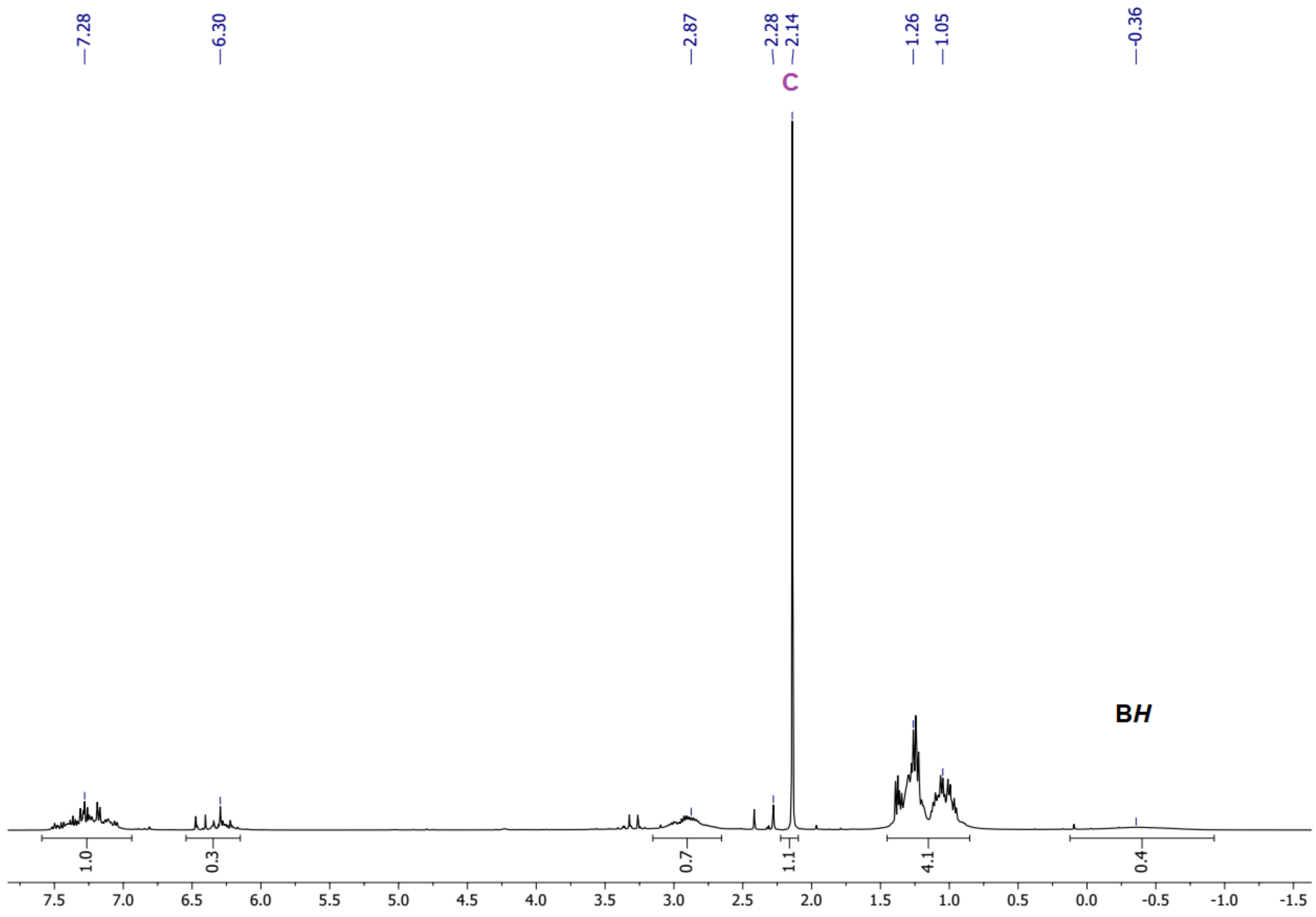

Figure S23. ^1H NMR spectrum (400.1 MHz, $CDCl_3$) of the conversion of **4** with approx. 3 equiv $H_3B{\cdot}SMe_2$ (recorded after 5 hours, not referenced to internal standard) showing an ill-defined mixture of species. **C**: Me_2S species.

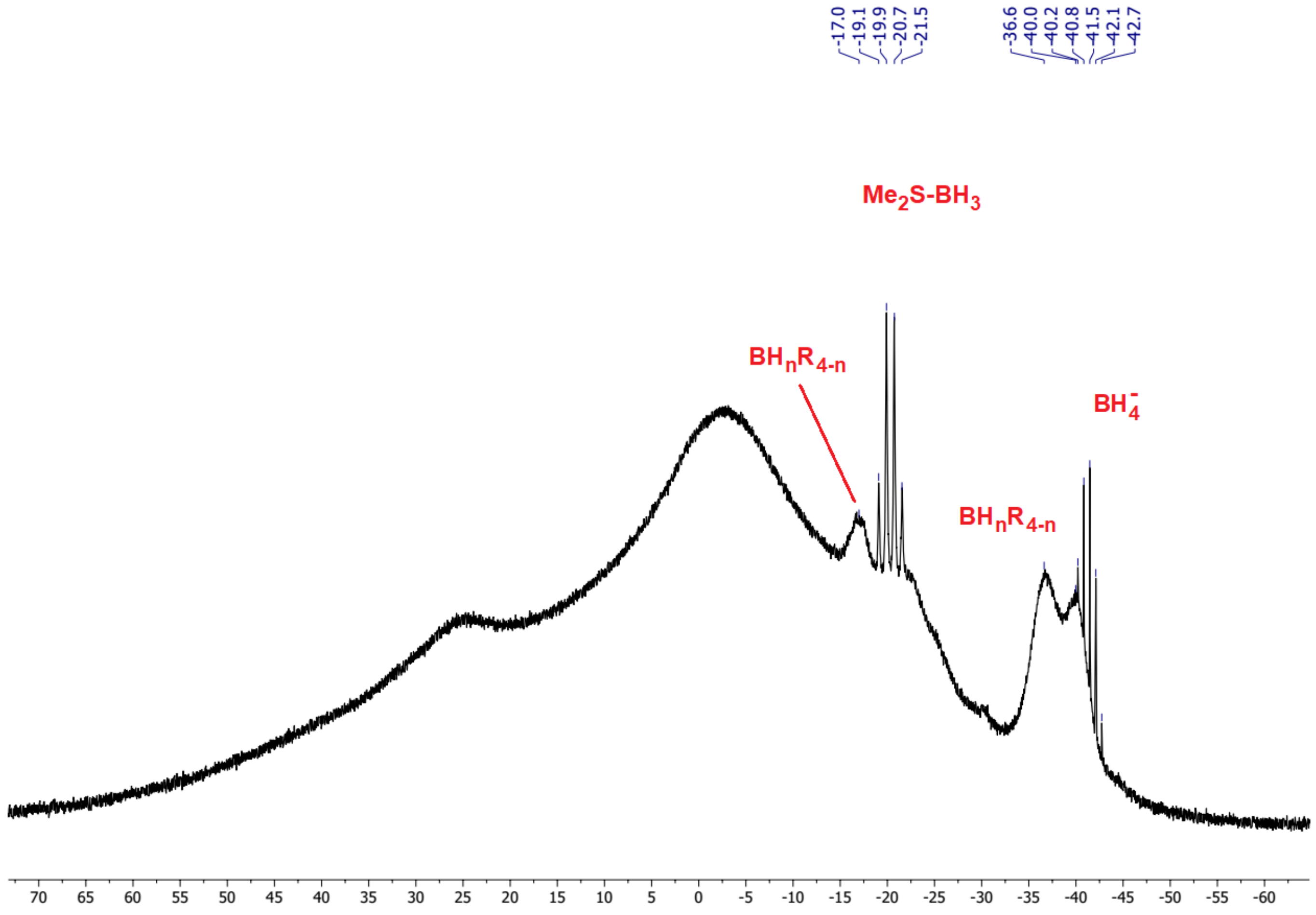

Figure S24. ^{11}B NMR spectrum (128.4 MHz, $CDCl_3$) of the conversion of **4** with approx. 3 equiv $Me_2S{\cdot}BH_3$ (recorded after 5 h, see main article for additional interpretation).

Procedure for the conversion of 6 with excess borane dimethylsulfide complex

To a solution of the tetracarboxylate **6** (31 mg, 0.04 mmol) in 0.5 mL $CDCl_3$ contained in an NMR sample tube were added few droplets of $H_3B \cdot SMe_2$ via syringe. The tube was shaken vigorously for mixing and NMR spectra recorded after indicated elapsed time (see captions of Figures below). The relative intensities of the Me_2S signal indicated that 6 equivalents of the borane dimethylsulfide complex had been added. See main article for spectra interpretation.

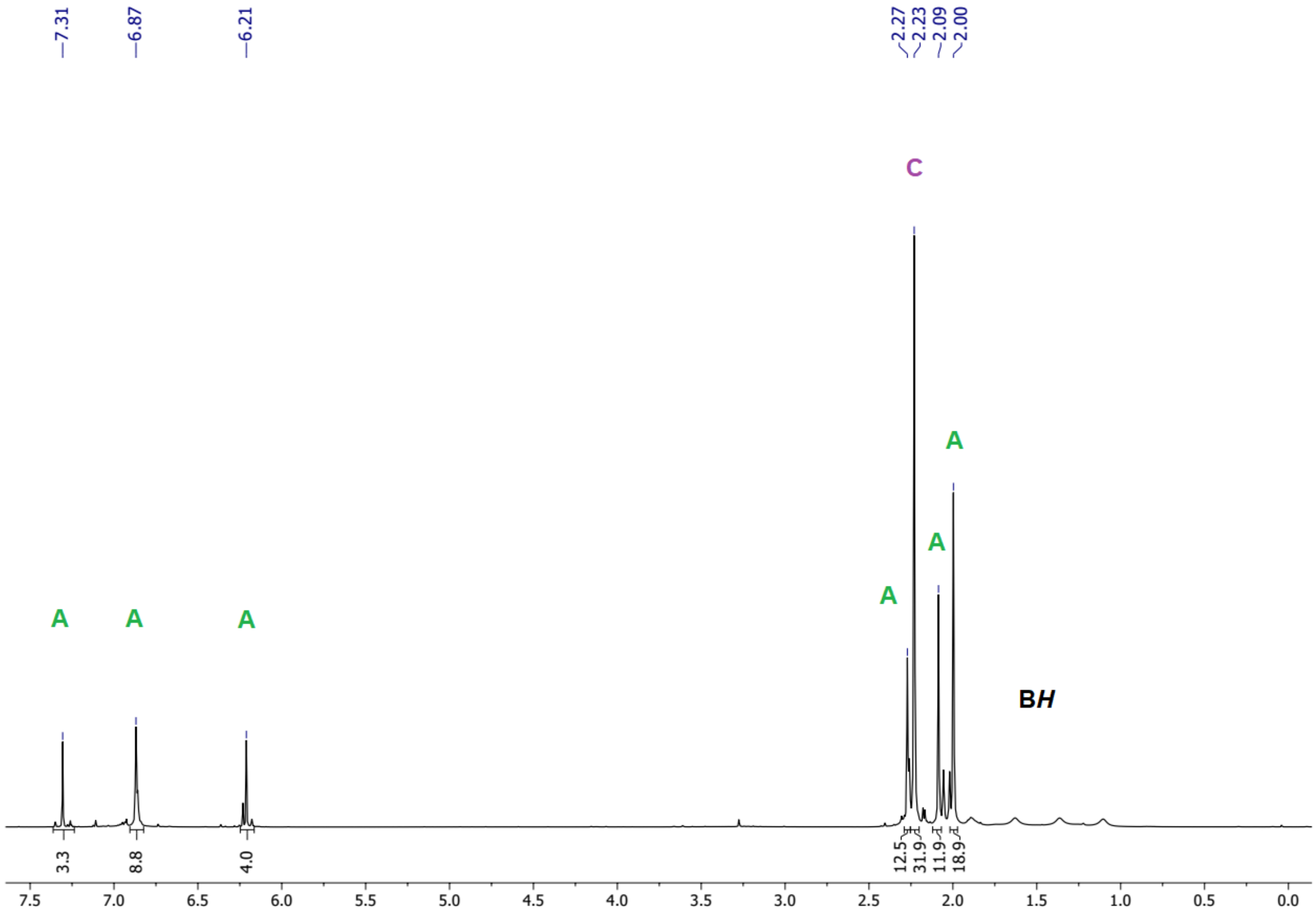

Figure S25. ^{1}H NMR spectrum (400.1 MHz, $CDCl_3$) of the conversion of **6** with 6 equiv $H_3B{\cdot}SMe_2$ (recorded after approx. 45 min) showing **6** as the major NHI-composed species (A). C: Me_2S species.

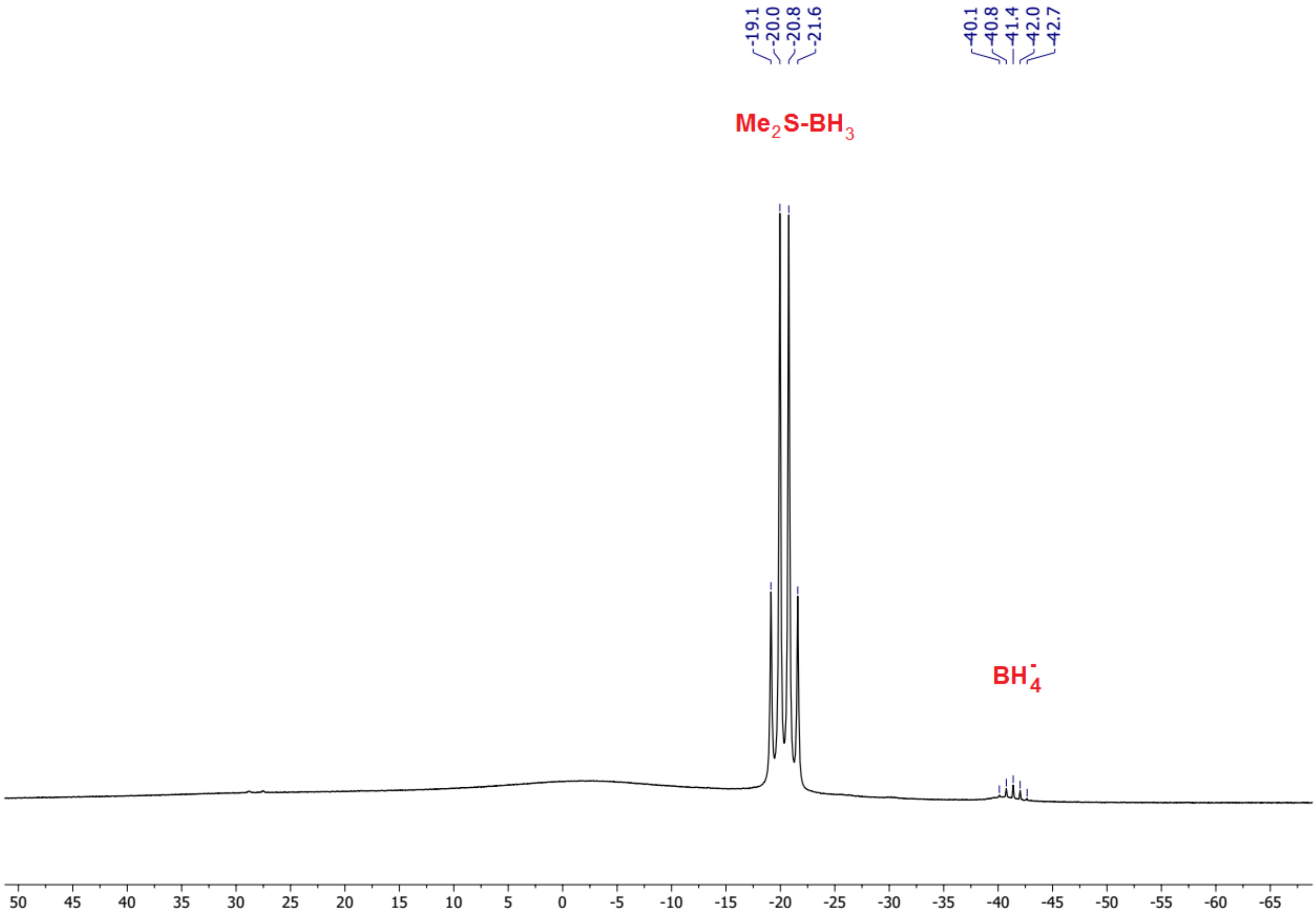

Figure S26. ^{11}B NMR spectrum (128.4 MHz, $CDCl_3$) of the conversion of **6** with 6 equiv $Me_2S{\cdot}BH_3$ (recorded after approx. 60 min).

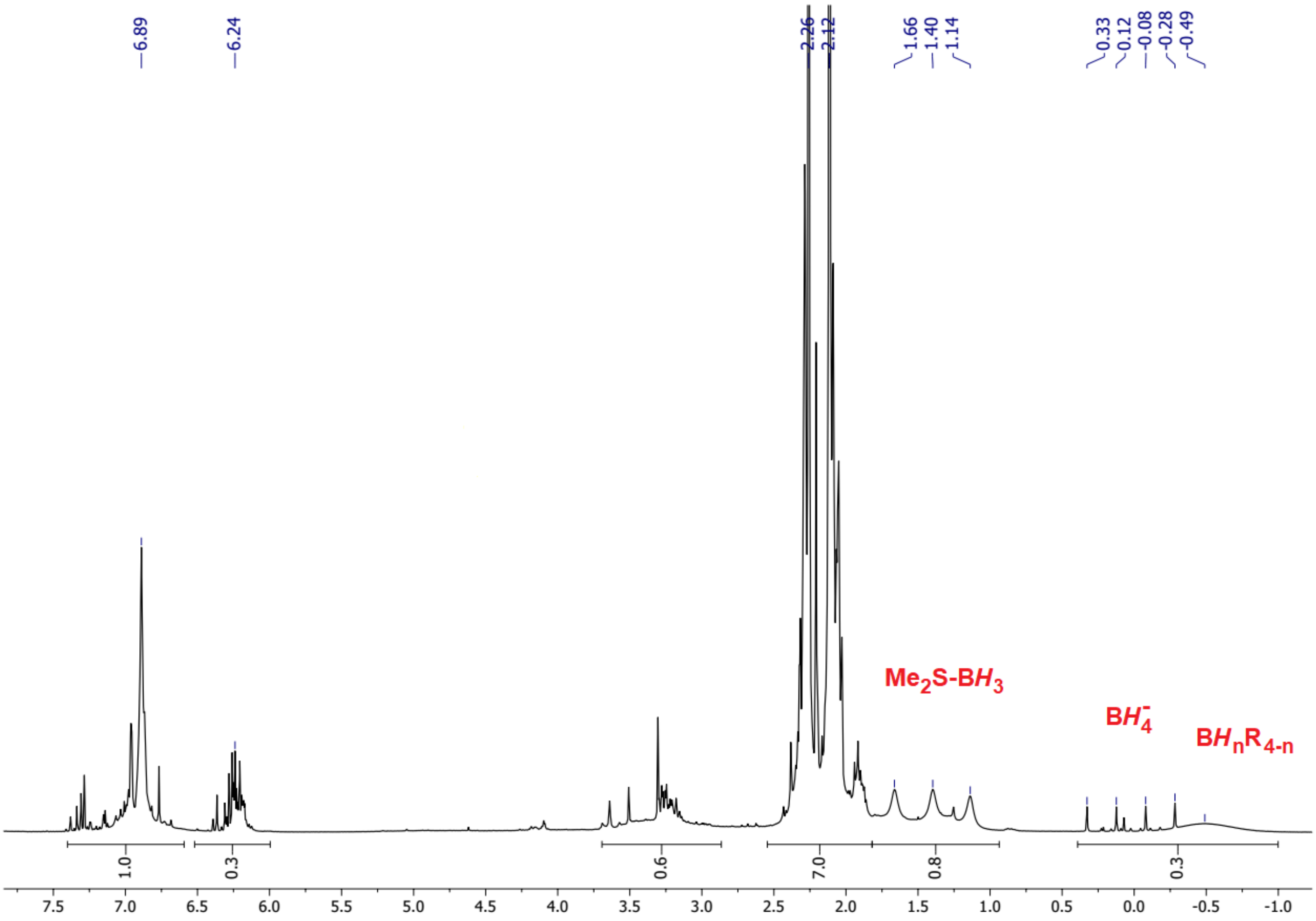

Figure S27. ^{1}H NMR spectrum (400.1 MHz, $CDCl_3$) of the conversion of **6** with 6 equiv $Me_2S{\cdot}BH_3$ (recorded after approx. 8 hours) showing an ill-defined mixture of species (not referenced to an internal standard, R = ligand).

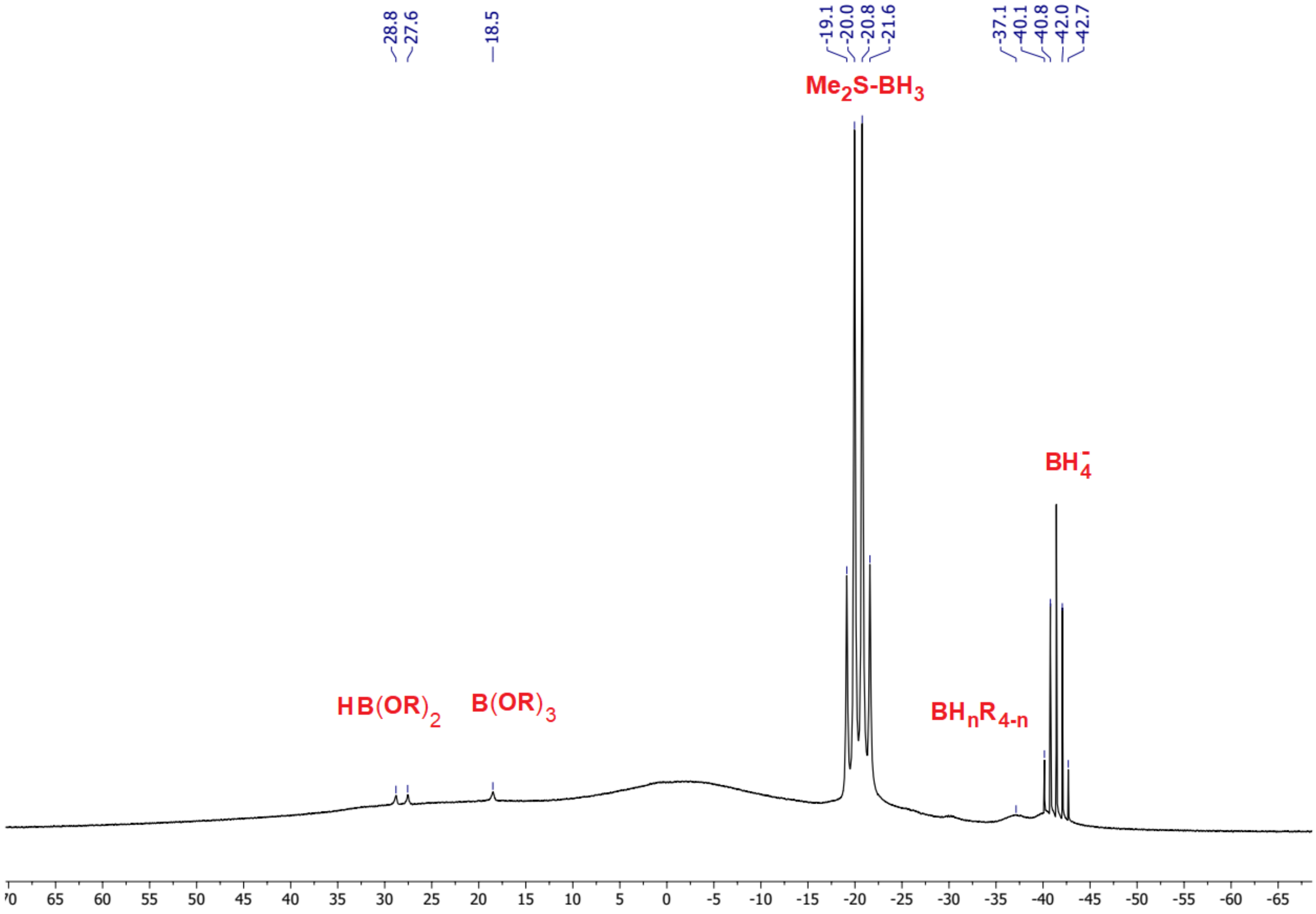

Figure S28. ^{11}B NMR spectrum (128.4 MHz, $CDCl_3$) of the conversion of **6** with 6 equiv $Me_2S{\cdot}BH_3$ (recorded after approx. 8.5 hours, R = ligand).

Procedure for the conversion of 11^+[OTs]$^-$ with excess borane dimethylsulfide complex

To a solution of **11**$^+$[OTs]$^-$ (25 mg, 0.03 mmol) in 0.5 mL $CDCl_3$ contained in an NMR sample tube were added few droplets of $H_3B{\cdot}SMe_2$ via syringe. The tube was shaken vigorously for mixing. The recording of NMR data commenced approximately 30 min after addition. The relative intensities of the Me_2S signals ("free" and borane-bonded) indicated that 7 equivalents of the borane dimethylsulfide complex had been added. The resonances (^1H, ^{11}B) corresponded to the formation of **10**$^+$ (compared to an authentic sample and fitting within the typical 0.01-0.02 ppm error margin/deviation) and a methoxyborane species (see below for depiction of spectra).

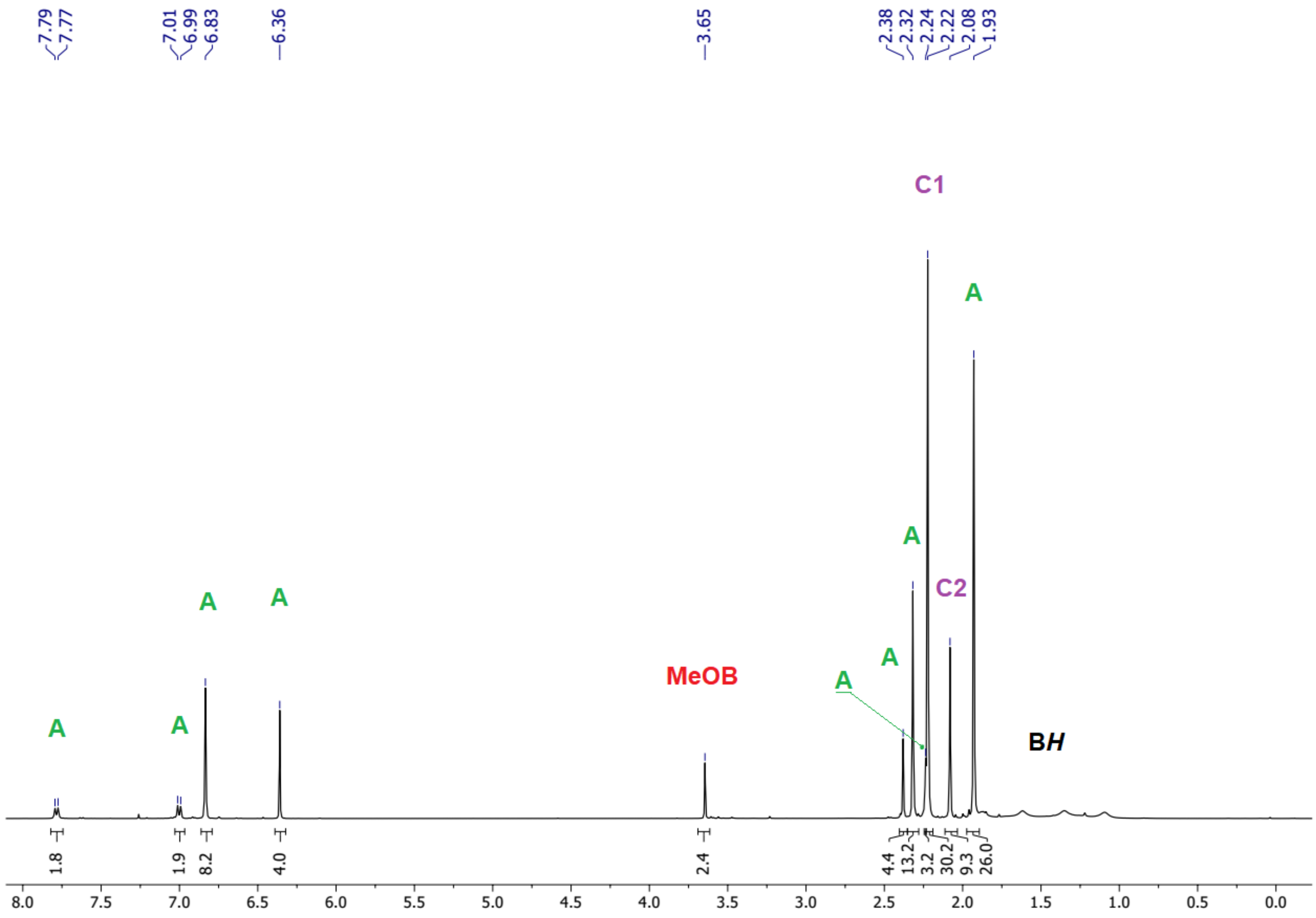

Figure S29. ^{1}H NMR spectrum (400.1 MHz, $CDCl_3$) of the conversion of **11**$^+$[OTs]$^-$ with excess $H_3B{\cdot}SMe_2$ (recorded after approx. 30 min) showing **10**$^+$[OTs]$^-$ (**A**) and a methoxyborane species (**MeOB**). **C1**: Me_2S; **C2**: "free" Me_2S.

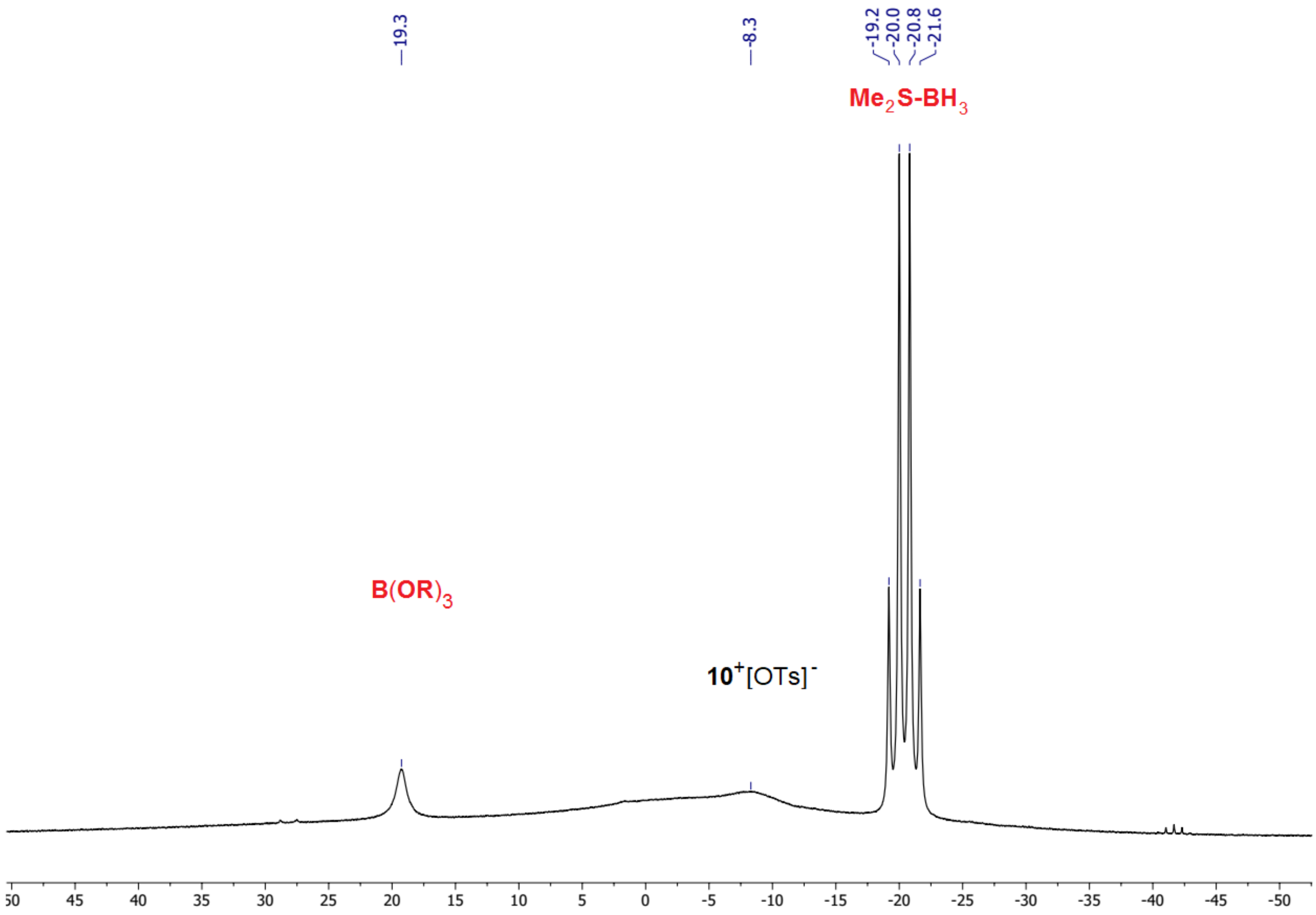

Figure S30. ^{11}B NMR spectrum (128.4 MHz, $CDCl_3$) of the conversion of **11**$^{+}$[OTs]$^{-}$ with excess $Me_2S{\cdot}BH_3$ showing **10**$^{+}$[OTs]$^{-}$, oxoborane (OR = methoxy or boroxide), and residual borane dimethylsulfide complex.

^{1}H NMR spectra demonstrating the stability of 1b in $CDCl_3$

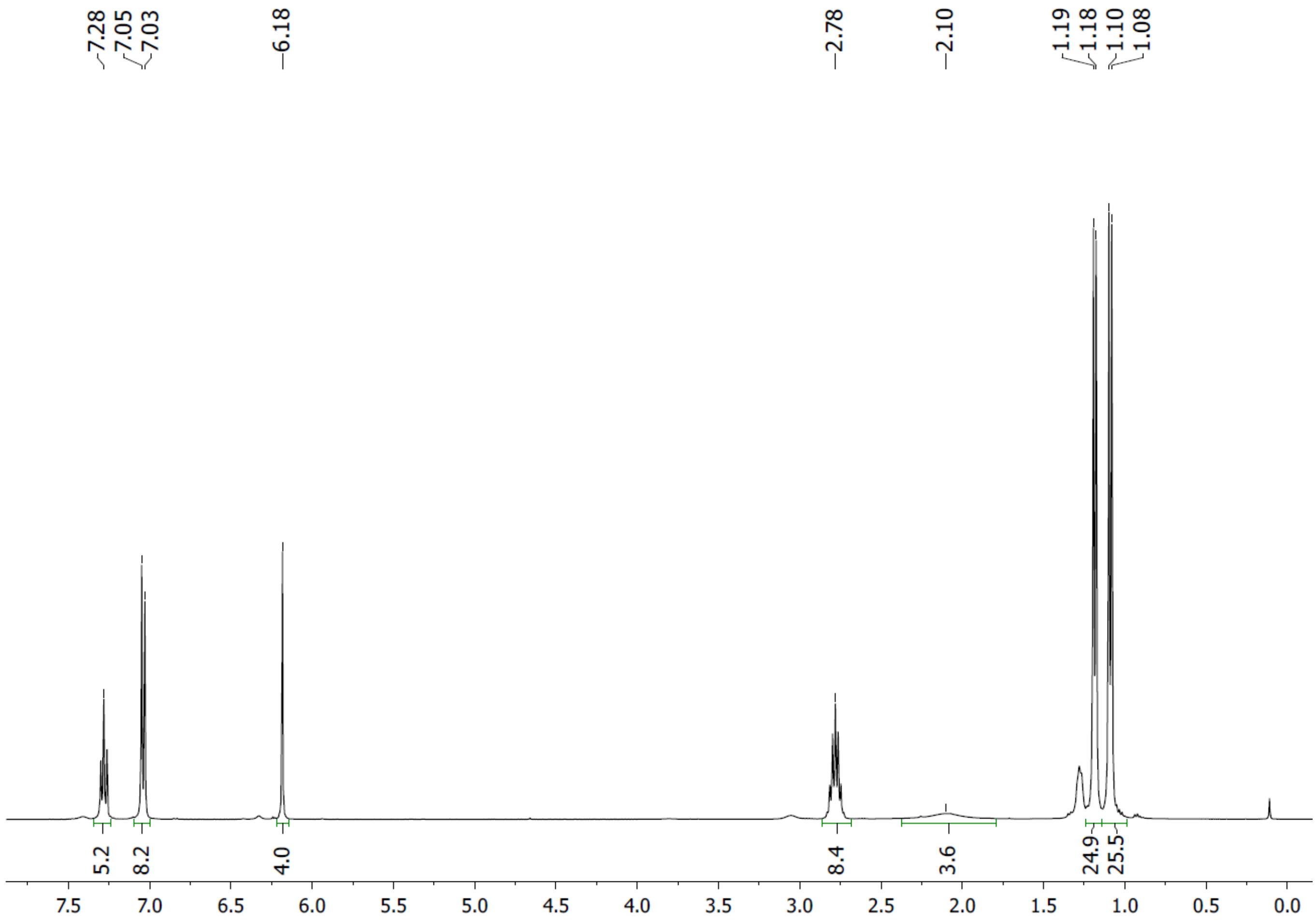

Figure S31. ^{1}H NMR spectrum (400.1 MHz, $CDCl_3$) of **1b** after storage of the sample solution for 24 h demonstrating the sufficient stability of the compound in this medium (note: Al*H* at 2.10; traces of the protonated ligand NHI-H contained).

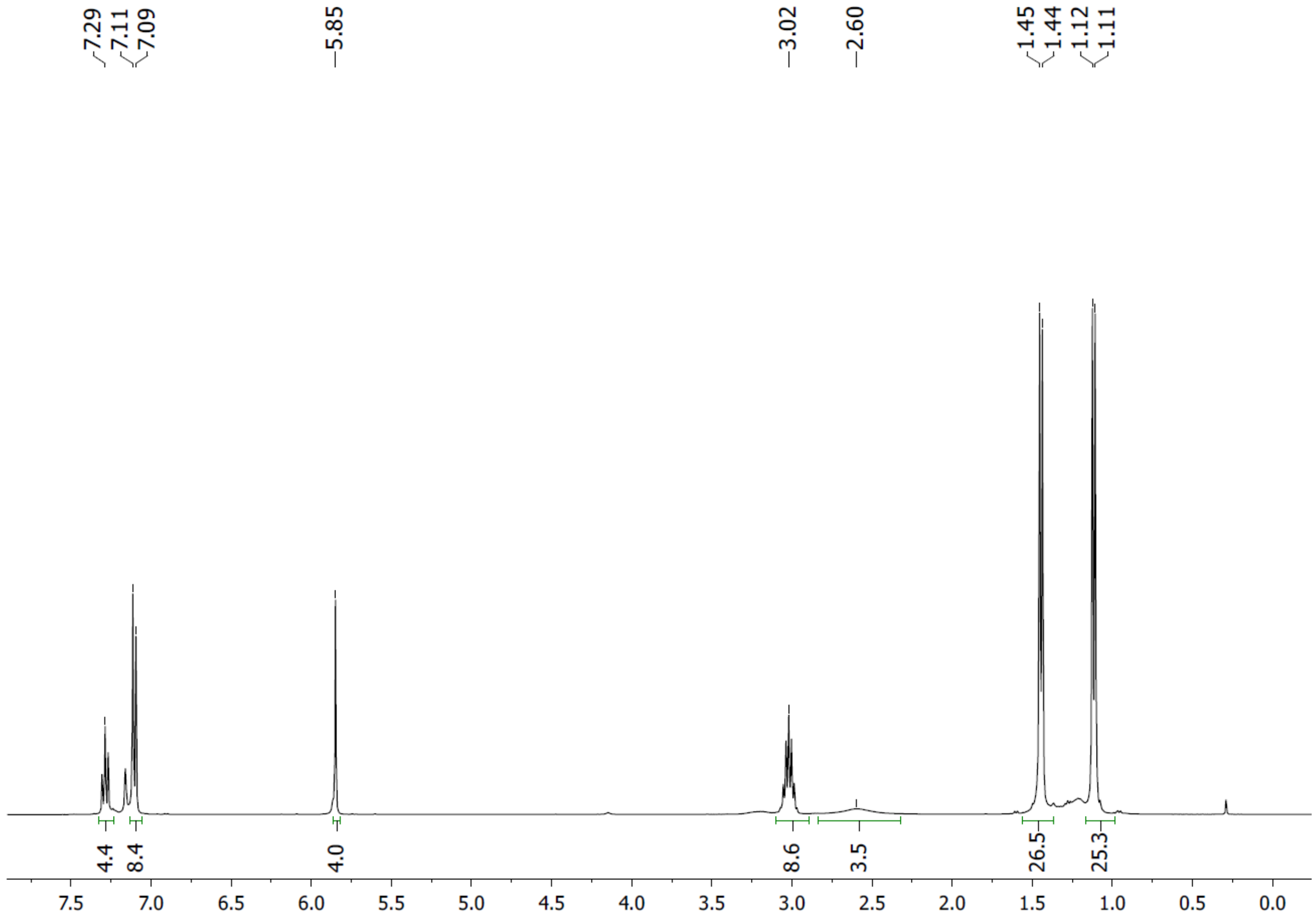

Figure S32. ^{1}H NMR spectrum (400.1 MHz, C_6D_6) of **1b** after evaporation of the solvent (i.e. $CDCl_3$) from the sample solution (see previous Figure for spectrum) under reduced pressure and redissolution of the residue in C_6D_6 (note: Al*H* at 2.60; traces of the protonated ligand NHI-H contained). This confirms the sufficient stability of **1b** in $CDCl_3$. Reference for the compound's proton NMR shifts in C_6D_6 can be found in the literature[S2].

General procedure for the catalytic CO_2 reduction with catecholborane (HBcat)

In a glovebox workstation a Schlenk vessel was charged with the (pre)catalyst, the naphthalene standard (only in selected instances), catecholborane and $CDCl_3$ in this order (in some instances the order of addition of catecholborane and $CDCl_3$ was inverse; the conversions were driven on a 2-4 mL scale with regard to the reaction-mixture volume, the relative amounts can be taken from Table 2 of the main article). At the Schlenk line borane dimethylsulfide complex was added and the resulting mixture frozen at liquid nitrogen temperature before setting the flask to vacuum. The liquid nitrogen bath was removed, the flask was pressurized with CO_2 (1.0-1.1 bar) and a water bath (fresh from "non-warm" tap) was applied for controlled thawing of the mixture with stirring. After temperature accomodation the water bath was removed and the reaction driven at room temperature with constant CO_2 pressure.

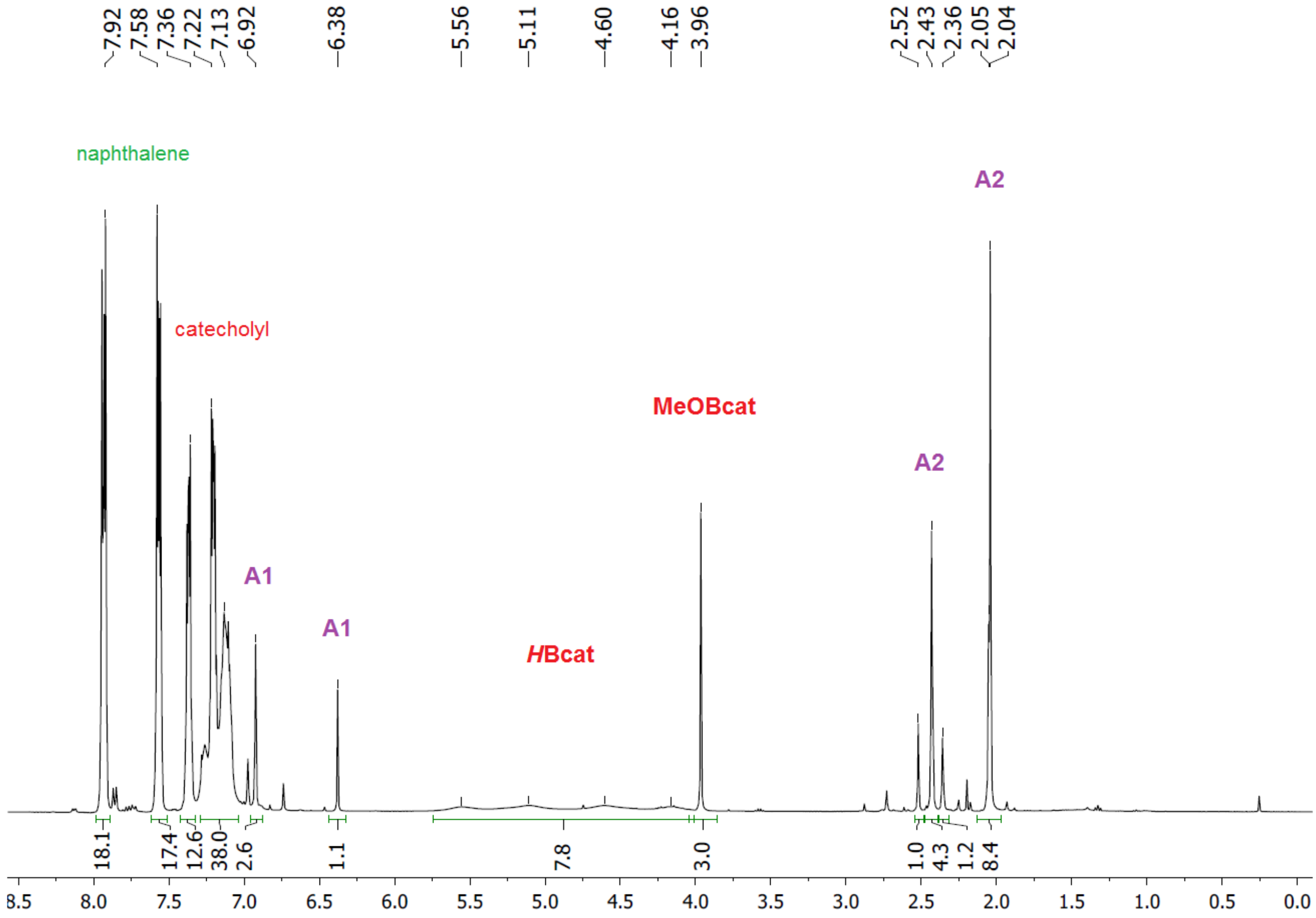

Figure S33. ^{1}H NMR spectrum (400.1 MHz, $CDCl_3$) of the conversion of catecholborane (HBcat) in CO_2 atmosphere using **11**$^+$[OTs]$^-$ as a (pre)catalyst after 4 h reaction time. **A1**: presumed signals of bis(NHI) species or HCOOBcat; **A2**: presumed signals of bis(NHI) species or tosylate (note: spin of ^{11}B nucleus = $^3/_2$, spectrum not referenced to an internal standard).

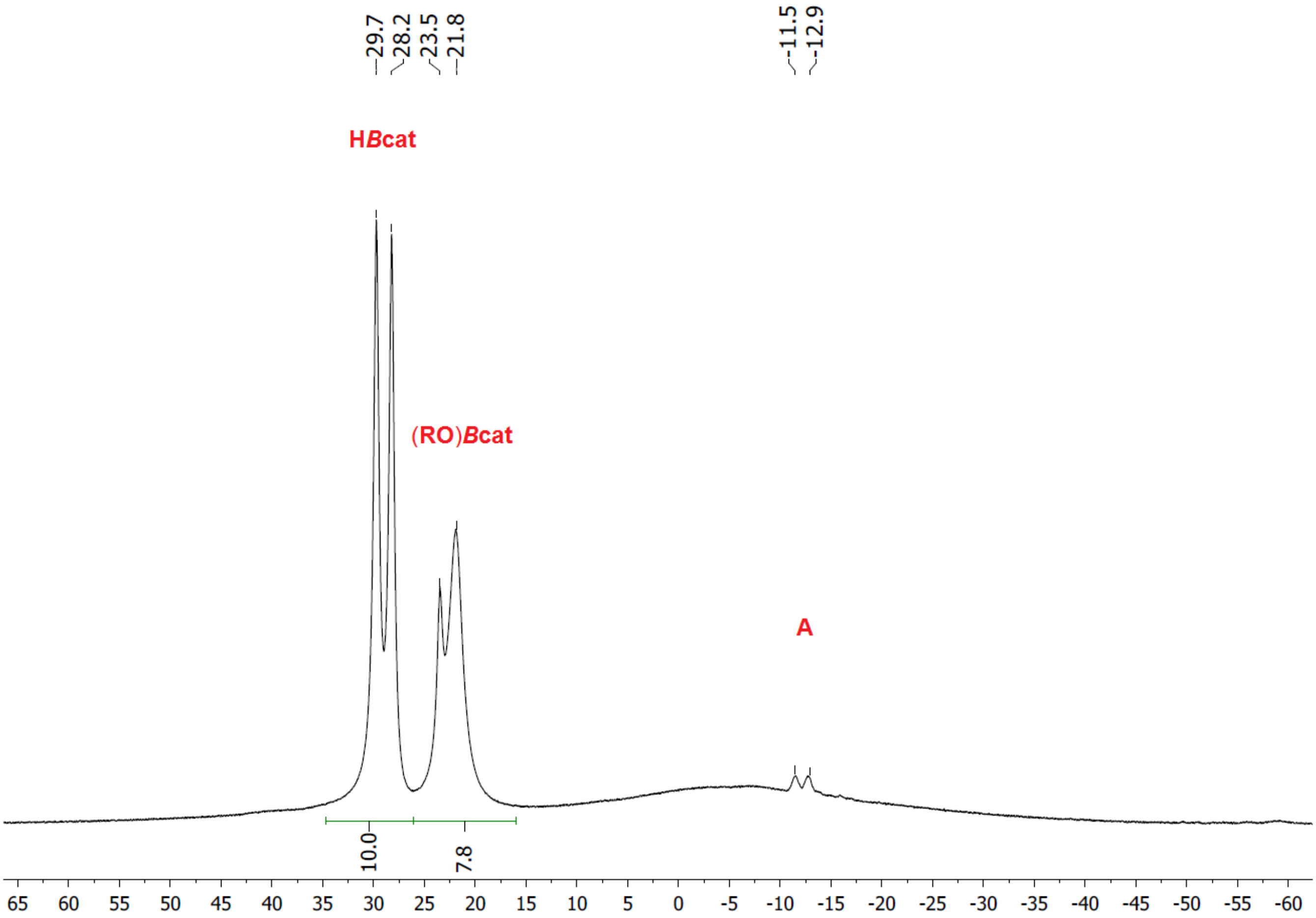

Figure S34. ^{11}B NMR spectrum (128.4 MHz, $CDCl_3$) of the conversion of catecholborane (HBcat) in CO_2 atmosphere using **11**$^+$[OTs]$^-$ as a (pre)catalyst after 4 h reaction time (RO = H_3CO, catBOCH_2O, HCOO or catBO). **A**: unassigned four-coordinate boron monohydride species.

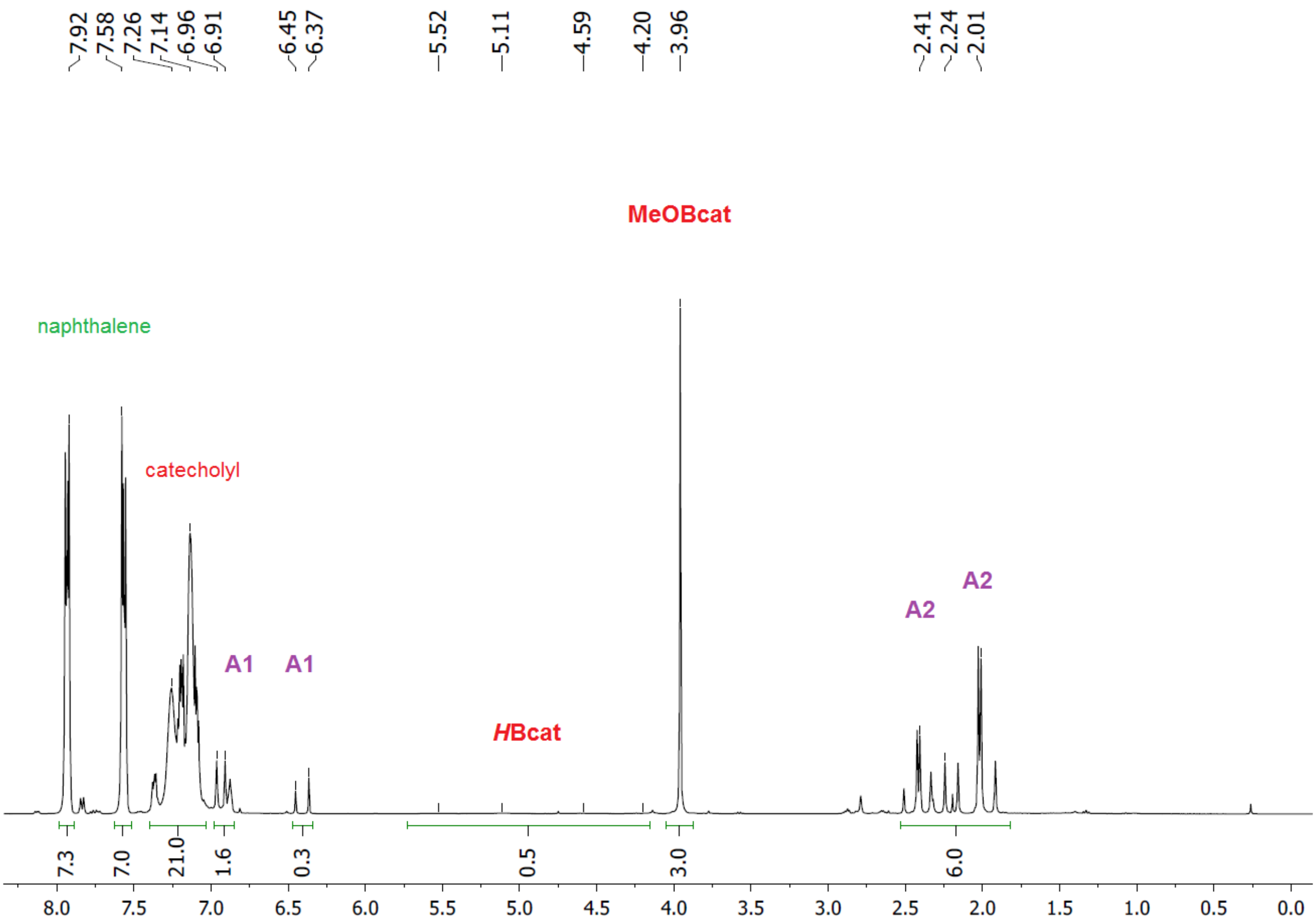

Figure S35. ^{1}H NMR spectrum (400.1 MHz, $CDCl_3$) of the conversion of catecholborane (HBcat) in CO_2 atmosphere using **11**$^{+}$[OTs]$^{-}$ as a (pre)catalyst after 15 h reaction time. **A1**: presumed signals of bis(NHI) species or HCOOBcat; **A2**: presumed signals of bis(NHI) species or tosylate (note: spin of ^{11}B nucleus = $^{3}/_{2}$, spectrum not referenced to an internal standard).

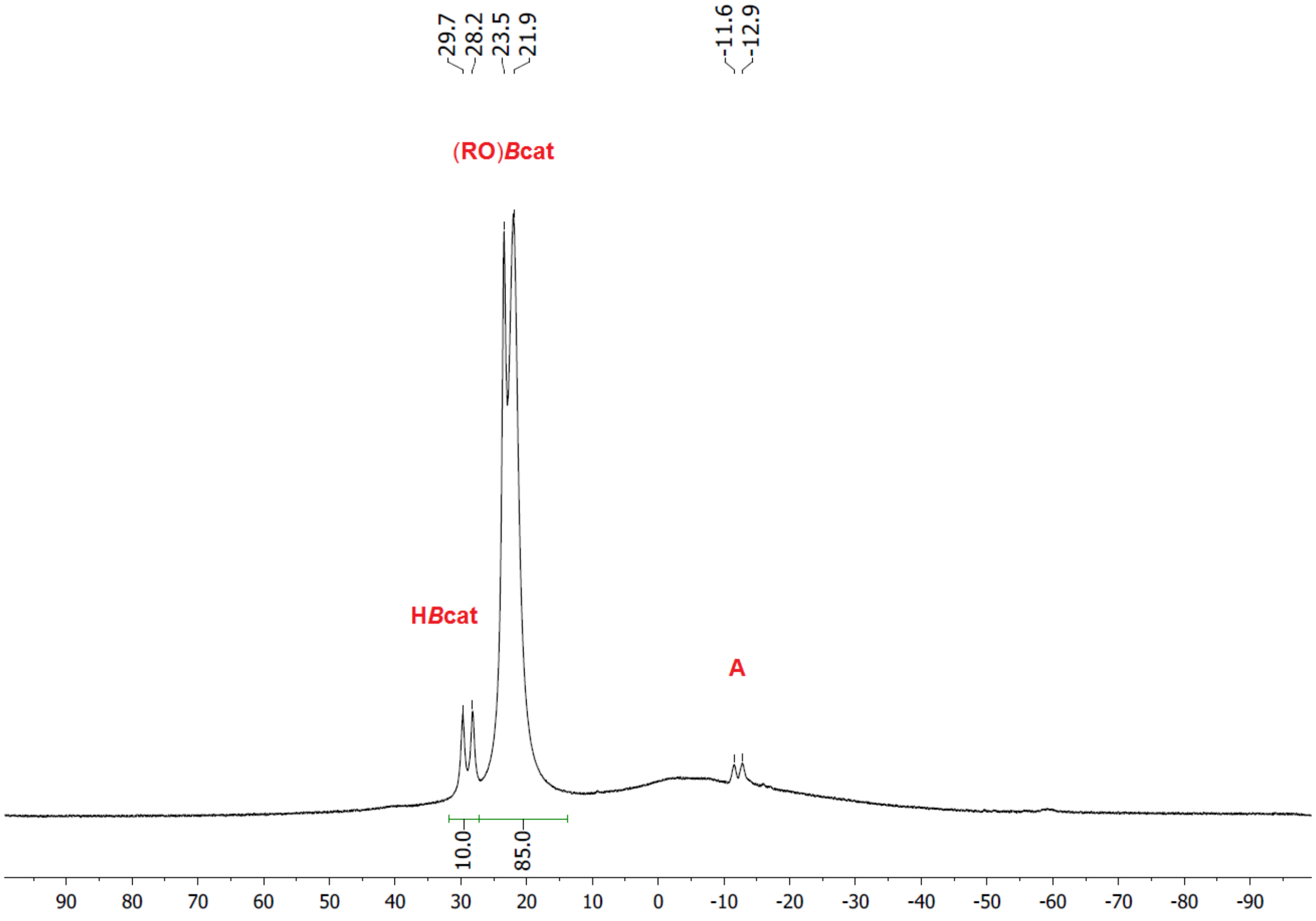

Figure S36. ^{11}B NMR spectrum (128.4 MHz, $CDCl_3$) of the conversion of catecholborane (HBcat) in CO_2 atmosphere using **11**$^{+}$[OTs]$^{-}$ as a (pre)catalyst after 15 h reaction time (RO = H_3CO, $catBOCH_2O$, HCOO or catBO). **A**: unassigned four-coordinate boron monohydride species.

Procedure for the catalytic CO_2 reduction with 9-borabicyclo[3.3.1]nonane (H-BBN)

The catalytic conversion was carried out as detailed for the use of catecholborane as a reductant using the "free" bisNHI **12** as a (pre)catalyst and H-BBN as a hydride source. See the main article for further interpretation.

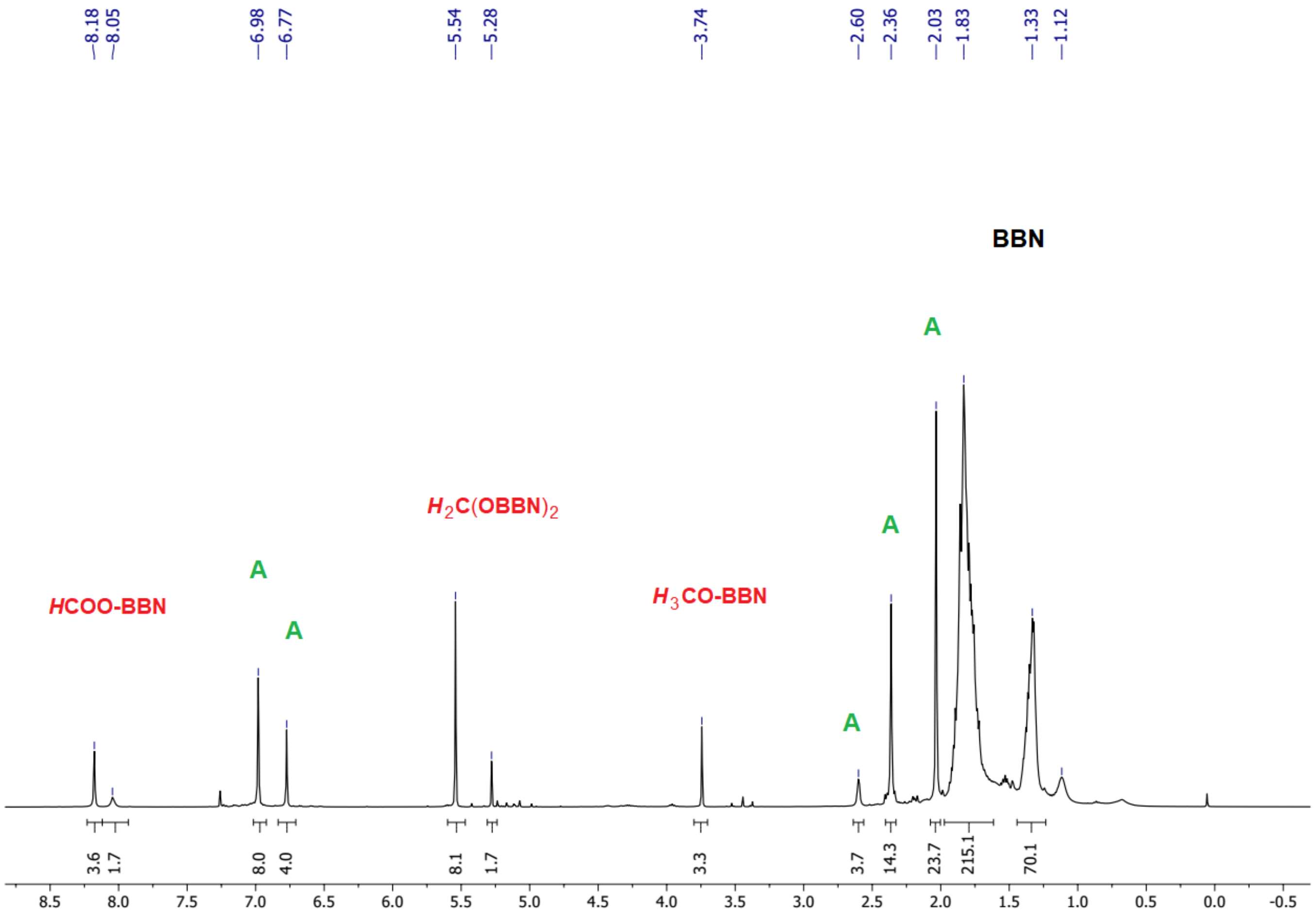

Figure S37. ^{1}H NMR spectrum (400.1 MHz, $CDCl_3$) of the conversion of H-BBN in CO_2 atmosphere using **12** as a (pre)catalyst after 13 h reaction time (BBN = 9-borabicyclo[3.3.1]nonyl). **A**: presumed signals of a bis(NHI) species.

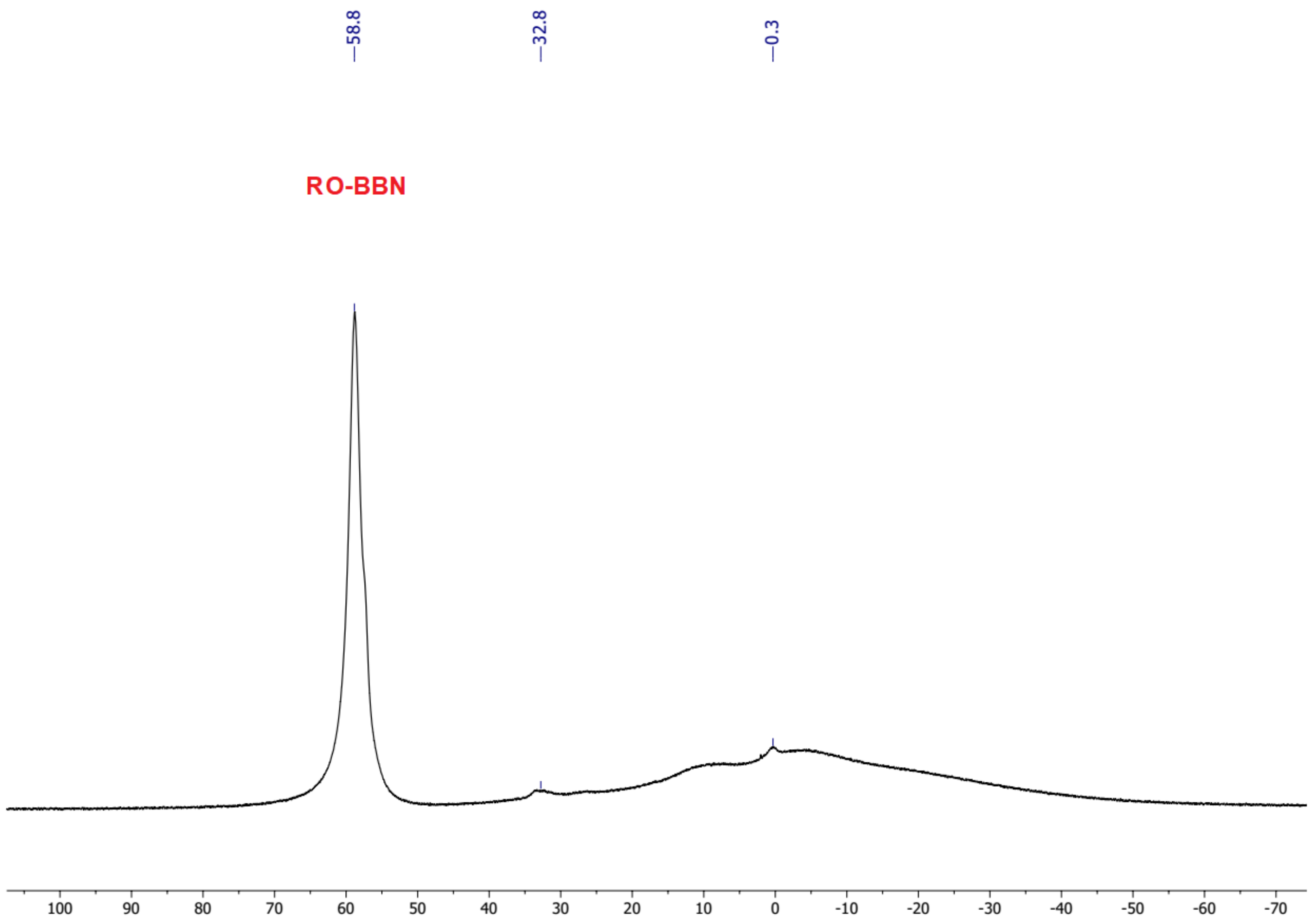

Figure S38. ^{11}B NMR spectrum (128.4 MHz, $CDCl_3$) of the conversion of H-BBN in CO_2 atmosphere using **12** as a (pre)catalyst after 13 h reaction time (BBN = 9-borabicyclo[3.3.1]nonyl, RO = H_3CO, (O-BBN)CH_2O, HCOO).

3.) X-ray Crystallographic Details

General Considerations: Data were collected on a single crystal x-ray diffractometer equipped with a CMOS detector (Bruker APEX III, κ-CMOS), an IMS microsource with MoK_α radiation (λ = 0.71073 Å) and a Helios optic using the APEX3 software package (Compounds **4**, **7**$^+$[Al(OC(CF_3)$_3$)$_4$]$^-$, **9**$^+$[OTs]$^-$) or on a single crystal x-ray diffractometer equipped with a CCD detector (Rigaku Oxford Diffraction, SuperNova, Atlas), a micro-focus sealed tube with CuK_α radiation (λ = 1.54184 Å) and a mirror monochromator using the CrysAlisPro software package (Compounds **6**, **11**$^+$[OTs]$^-$).[S5,S6] The measurements were performed on single crystals coated with perfluorinated polyether oil. The crystals were fixed on top of a kapton micro sampler and frozen under a stream of cold nitrogen. A matrix scan was used to determine the initial lattice parameters. Reflections were corrected for Lorentz and polarisation effects, scan speed, and background using the CrysAlisPro software package or SAINT.[S6,S7] Absorption correction, including odd and even ordered spherical harmonics was performed using the CrysAlisPro software package or SADABS.[S6,S7] Space group assignment was based upon systematic absences, E statistics, and successful refinement of the structure. The structures were solved using SHELXT with the aid of successive difference Fourier maps, and were refined against all data using SHELXL-2014 in conjunction with SHELXLE.[S8,S9,S10] Hydrogen atoms – except if bound to Al or B – were calculated in ideal positions as follows: Methyl hydrogen atoms were refined as part of rigid rotating groups, with a C–H distance of 0.98 Å and $U_{iso(H)} = 1.5 \cdot U_{eq(C)}$. Other H atoms were placed in calculated positions and refined using a riding model, with methylene and aromatic C–H distances of 0.99 Å and 0.95 Å, respectively, and other C–H distances of 1.00 Å, all with $U_{iso(H)} = 1.2 \cdot U_{eq(C)}$. In the case of **7**, the Al-H distances were constrained at 1.51 Å. Non-hydrogen atoms were refined with anisotropic displacement parameters. Full-matrix least-squares refinements were carried out by minimizing $\Sigma w(F_o^2 - F_c^2)^2$ with the SHELXL weighting scheme.[S8] Neutral atom scattering factors for all atoms and anomalous dispersion corrections for the non-hydrogen atoms were taken from *International Tables for Crystallography*.[S11] A split layer refinement was used to treat with disordered groups and additional restraints on distances, angles and anisotropic displacements parameters were employed to achieve convergence within physically meaningful limits. The unit cell of **9**$^+$[OTs]$^-$ contains two molecules of tetrahydrofuran and two molecules of pentane, the unit cell of **11**$^+$[OTs]$^-$ contains four molecules of dichloromethane with partial tetrahydrofuran occupancy and the unit cell of **6** contains twelve molecules of chloroform; these were treated as a diffuse contribution to the overall scattering without specific atom positions using the PLATON/SQUEEZE procedure.[S12] Images of the crystal structure were generated with Mercury and PLATON.[S13,S14] The supplementary crystallographic data for this publication can be acquired free of charge by The Cambridge Crystallographic Data Centre via **CCDC Reference Numbers: 1940504 to 1940508**

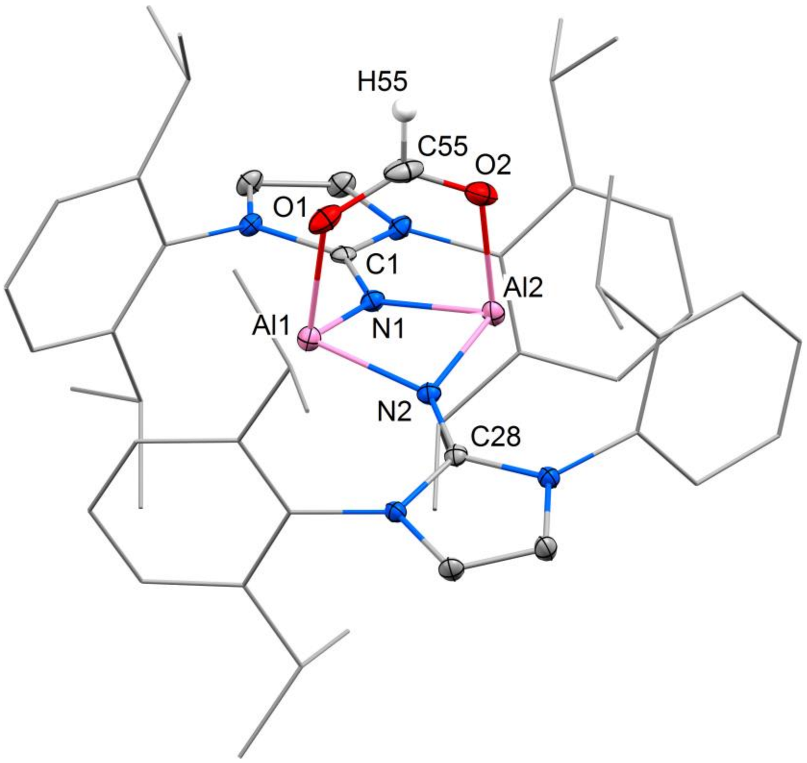

Figure S39. Molecular structure of 7^{+} (in $7^{+}[Al(OR^{F})_4]^{-}$) in the solid state as derived from SCXRD analysis (thermal ellipsoids are depicted at the 30% level). Dip groups are depicted as wireframe model. Structure refinement afforded two independent molecules in the asymmetric unit and only one is shown. One Al center of each molecule bears an exocyclic formate group as a minor occupied site instead of a hydride (not shown, occupancy factor for exocyclic formate = ca. 20%). Hydrogen atoms omitted except at formate. Selected bond lengths [Å], angles [°], and atom···atom distance [Å] (equivalent value for second molecule in brackets): Al1-O1 = 1.879(3) [1.883(3)], Al2-O2 = 1.884(3) [1.889(3)], Al1-N1 = 1.875(3) [1.885(3)], Al1-N2 = 1.878(3) [1.885(3)], O1-C55 = 1.261(6) [1.261(6)], O2-C55 = 1.256(6) [1.257(6)], N1-C1 = 1.317(5) [1.305(4)], N2-C28 = 1.325(4) [1.317(4)]; O1-Al1-N1 = 97.1(1) [98.1(1)], O1-Al1-N2 = 96.0(1) [94.5(1)], Al2···Al1-O1 = 84.4(1) [84.3(1)], O1-C55-O2 = 127.3(4) [126.7(4)]; Al···Al = 2.630(2) [2.632(2)].

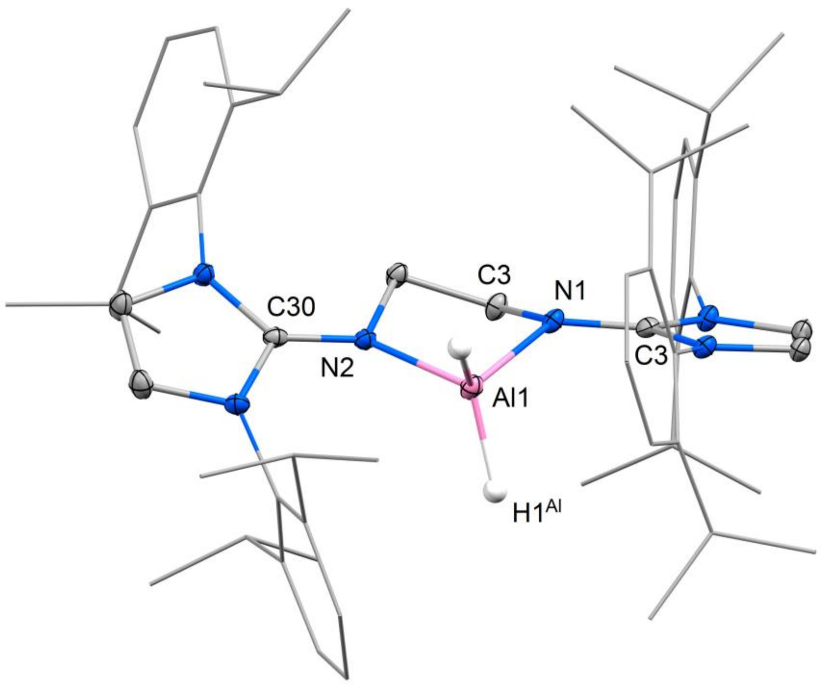

Figure S40. Molecular structure of **9**$^+$ (in **9**$^+$[OTs]$^-$(solvent)) as derived from SCXRD study. Thermal ellipsoids are depicted at the 30% level. The Dip groups are shown as wireframe model. Hydrogen atoms omitted except at Al. Selected bond lengths [Å] and angles [°]: Al1–N1 = 1.942(2), Al1–N2 = 1.924(2), N1–C3 = 1.332(2), N2–C30 = 1.326(2); N1–Al1–N2 = 86.8(1), Al1–N1–C3 = 126.4(1), Al1–N1–C1 =107.1(1), C1–N1–C3 = 119.4(1).

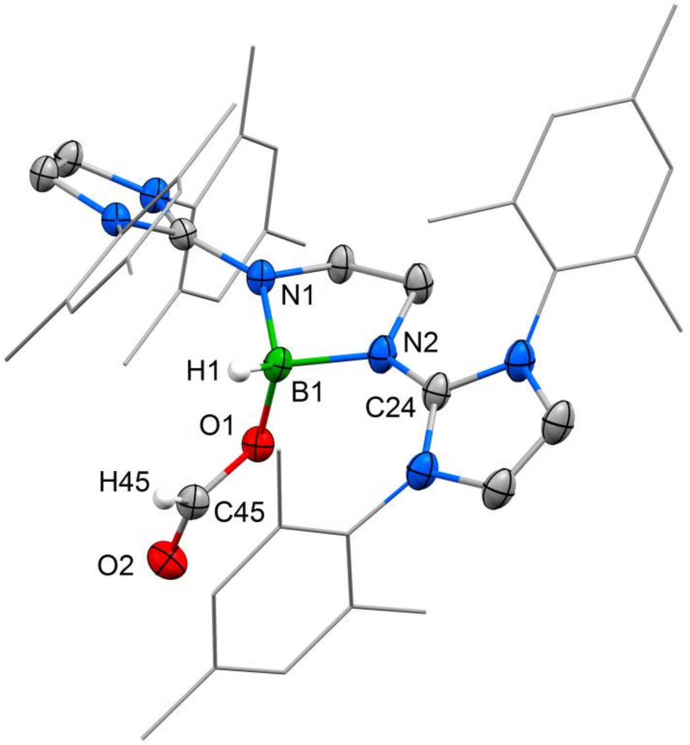

Figure S41. Molecular structure of $\mathbf{11}^+$ (in $\mathbf{11}^+[OTs]^-$) in the solid state as derived from SCXRD analysis (thermal ellipsoids are depicted at the 30% level). Mes groups are depicted as wireframe model. Hydrogen atoms omitted except at boron. Selected bond lengths and angles: B1-O1 = 1.505(2), B1-N1 = 1.556(3), B1-N2 = 1.554(3), O1-C45 = 1.312(2), O2-C45 = 1.206(2), N2-C24 = 1.340(3); O1-B1-N1 =106.7(1), O1-B1-N2 = 109.4(2), N1-B1-N2 = 97.8(1), O1-C45-O2 = 123.8(2).

Compound 4 (CCDC 1940506)

Crystal data

$C_{56}H_{76}Al_2N_6O_4$

M_r = 951.19

D_x = 1.168 Mg m^{-3}

Monoclinic, $P2_1/n$

Hall symbol: -P 2yn

Mo *Kα* radiation, λ = 0.71073 Å

a = 12.6201 (6) Å

Cell parameters from 9456 reflections

b = 14.2219 (7) Å

θ = 2.4–25.7°

c = 15.4638 (8) Å

μ = 0.10 mm^{-1}

β = 102.884 (2)°

T = 249 K

V = 2705.6 (2) Å^3

Fragment, clear colourless

Z = 2

0.36 × 0.36 × 0.34 mm

F(000) = 1024

Data collection

Bruker Photon CMOS diffractometer

4766 independent reflections

Radiation source: IMS microsource

4327 reflections with $I > 2\sigma(I)$

Helios optic monochromator

R_{int} = 0.040

Detector resolution: 16 pixels mm^{-1}

θ_{max} = 25.0°, θ_{min} = 2.2°

phi– and ω–rotation scans

h = −15 15

Absorption correction: multi-scan *SADABS* 2014/5, Bruker

k = −16 16

T_{min} = 0.654, T_{max} = 0.745

l = −18 18

109825 measured reflections

Refinement

Refinement on F^2

Least-squares matrix: full

$R[F^2 > 2\sigma(F^2)]$ = 0.034

$wR(F^2)$ = 0.088

S = 1.05

4766 reflections

319 parameters

0 restraints

0 constraints

Primary atom site location: iterative

Secondary atom site location: difference Fourier map

Hydrogen site location: mixed

H atoms treated by a mixture of independent and constrained refinement

$W = 1/[\Sigma^2(FO^2) + (0.0422P)^2 + 1.4842P]$ WHERE $P = (FO^2 + 2FC^2)/3$

$(\Delta/\sigma)_{max}$ = 0.001

$\Delta\rho_{max}$ = 0.30 e Å^{-3}

$\Delta\rho_{min}$ = −0.30 e Å^{-3}

Extinction correction: none

Extinction coefficient: -

Compound 6 (CCDC 1940508)

Crystal data

$C_{46}H_{52}Al_2N_6O_8$

M_r = 870.90	D_x = 0.983 Mg m^{-3}
Monoclinic, $P2_1/c$	Melting point: ? K
Hall symbol: -P 2ybc	Cu *K*α radiation, λ = 1.54184 Å
a = 14.3408 (1) Å	Cell parameters from 22211 reflections
b = 14.2174 (1) Å	θ = 3.1–73.8°
c = 29.6859 (3) Å	μ = 0.82 mm^{-1}
β = 103.437 (1)°	*T* = 150 K
V = 5886.94 (9) Å^3	Block, colourless
Z = 4	0.28 × 0.26 × 0.15 mm
F(000) = 1840	

Data collection

SuperNova, Single source at offset/far, Atlas diffractometer	11730 independent reflections
Radiation source: micro-focus sealed X-ray tube, SuperNova (Cu) X-ray Source	10575 reflections with $I > 2\sigma(I)$
Mirror monochromator	R_{int} = 0.034
Detector resolution: 10.5435 pixels mm^{-1}	θ_{max} = 73.9°, θ_{min} = 3.1°
ω scans	*h* = −17 16

Absorption correction: multi-scan	
CrysAlis PRO 1.171.39.46 (Rigaku Oxford Diffraction)	$k = -17$ 17
T_{min} = 0.602, T_{max} = 1.000	$l = -36$ 35
40846 measured reflections	

Refinement

Refinement on F^2	Secondary atom site location: difference Fourier map
Least-squares matrix: full	Hydrogen site location: inferred from neighbouring sites
$R[F^2 > 2\sigma(F^2)]$ = 0.050	H-atom parameters constrained
$wR(F^2)$ = 0.133	W = $1/[\Sigma^2(FO^2) + (0.0539P)^2 + 4.2647P]$ WHERE $P = (FO^2 + 2FC^2)/3$
S = 1.07	$(\Delta/\sigma)_{max}$ = 0.001
11730 reflections	$\Delta\rho_{max}$ = 0.31 e Å^{-3}
571 parameters	$\Delta\rho_{min}$ = −0.39 e Å^{-3}
0 restraints	Extinction correction: none
0 constraints	Extinction coefficient: -
Primary atom site location: iterative	

Compound 7$^+$[Al(OC(CF$_3$)$_3$)$_4$]$^-$ (CCDC 1940505)

Crystal data

$C_{55.20}H_{75}Al_2N_6O_{2.39}·C_{16}AlF_{36}O_4$

M_r = 1882.22	D_x = 1.506 Mg m^{-3}
Orthorhombic, $P2_12_12_1$	
Hall symbol: P 2ac 2ab	Mo *K*α radiation, λ = 0.71073 Å
a = 19.220 (4) Å	Cell parameters from 9510 reflections
b = 19.471 (4) Å	θ = 2.4–25.4°
c = 44.366 (9) Å	μ = 0.18 mm^{-1}
V = 16603 (6) Å^3	*T* = 100 K
Z = 8	Fragment, colourless
F(000) = 7667.6	0.54 × 0.30 × 0.18 mm

Data collection

Bruker Photon CMOS diffractometer	31578 independent reflections
Radiation source: IMS microsource	28039 reflections with $I > 2\sigma(I)$
Helios optic monochromator	R_{int} = 0.048
Detector resolution: 16 pixels mm^{-1}	θ_{max} = 25.7°, θ_{min} = 2.1°
phi– and ω–rotation scans	*h* = −23 23
Absorption correction: multi-scan *SADABS* 2016/2, Bruker	*k* = −23 23
T_{min} = 0.677, T_{max} = 0.745	*l* = −54 54
155696 measured reflections	

Refinement

Refinement on F^2

Least-squares matrix: full

$R[F^2 > 2\sigma(F^2)] = 0.042$

$wR(F^2) = 0.101$

$S = 1.04$

31578 reflections

2594 parameters

1264 restraints

0 constraints

Primary atom site location: iterative

Secondary atom site location: difference Fourier map

Hydrogen site location: mixed

H atoms treated by a mixture of independent and constrained refinement

$W = 1/[\Sigma^2(FO^2) + (0.0427P)^2 + 11.0669P]$ WHERE $P = (FO^2 + 2FC^2)/3$

$(\Delta/\sigma)_{max} = 0.001$

$\Delta\rho_{max} = 0.46$ e Å^{-3}

$\Delta\rho_{min} = -0.29$ e Å^{-3}

Extinction correction: none

Extinction coefficient: ?-

Absolute structure: Flack

Absolute structure parameter: −0.04 (4)

Compound $9^{+}[OTs]^{-}$ (CCDC 1940507)

Crystal data

$C_{56}H_{78}AlN_{6}\cdot C_{7}H_{7}O_{3}S$	
M_r = 1033.41	
Triclinic, *P*	D_x = 1.053 Mg m^{-3}
Hall symbol: -P 1	
a = 12.469 (2) Å	Mo *K*α radiation, λ = 0.71073 Å
b = 21.606 (4) Å	Cell parameters from 9720 reflections
c = 25.270 (5) Å	θ = 2.2–25.8°
α = 74.692 (5)°	μ = 0.11 mm^{-1}
β = 83.204 (5)°	*T* = 100 K
γ = 89.748 (5)°	Fragment, colourless
V = 6518 (2) Å^3	0.24 × 0.16 × 0.08 mm
Z = 4	*F*(000) = 2232

Data collection

Bruker Photon CMOS diffractometer	25155 independent reflections
Radiation source: IMS microsource	19394 reflections with $I > 2\sigma(I)$
Helios optic monochromator	R_{int} = 0.048
Detector resolution: 16 pixels mm^{-1}	θ_{max} = 25.9°, θ_{min} = 2.2°
phi– and ω–rotation scans	*h* = −15 15
Absorption correction: multi-scan *SADABS* 2014/5, Bruker	*k* = −26 26
T_{min} = 0.697, T_{max} = 0.745	*l* = −30 31

194418 measured reflections

Refinement

Refinement on F^2

Least-squares matrix: full

$R[F^2 > 2\sigma(F^2)] = 0.046$

$wR(F^2) = 0.116$

$S = 1.02$

25155 reflections

1496 parameters

450 restraints

0 constraints

Primary atom site location: iterative

Secondary atom site location: difference Fourier map

Hydrogen site location: mixed

H atoms treated by a mixture of independent and constrained refinement

$W = 1/[\Sigma^2(FO^2) + (0.0483P)^2 + 4.2684P]$ WHERE $P = (FO^2 + 2FC^2)/3$

$(\Delta/\sigma)_{max} = 0.001$

$\Delta\rho_{max} = 0.29$ e Å^{-3}

$\Delta\rho_{min} = -0.49$ e Å^{-3}

Extinction correction: none

Extinction coefficient: -

Compound 11$^+$[OTs]$^-$ (CCDC 1940504)

Crystal data

$C_{45}H_{54}BN_6O_2{\cdot}C_7H_7O_3S$

M_r = 892.94	D_x = 1.145 Mg m^{-3}
Monoclinic, $P2_1/n$	Melting point: ? K
Hall symbol: -P 2yn	Cu *Kα* radiation, λ = 1.54184 Å
a = 17.2954 (4) Å	Cell parameters from 12642 reflections
b = 18.8394 (3) Å	θ = 3.0–73.4°
c = 17.6791 (4) Å	μ = 0.95 mm^{-1}
β = 115.981 (3)°	*T* = 150 K
V = 5178.3 (2) Å^3	Plate, colourless
Z = 4	0.27 × 0.08 × 0.08 mm
F(000) = 1904	

Data collection

SuperNova, Single source at offset, Atlas diffractometer	10313 independent reflections
Radiation source: micro-focus sealed X-ray tube, SuperNova (Cu) X-ray Source	8045 reflections with $I > 2\sigma(I)$
Mirror monochromator	R_{int} = 0.042
Detector resolution: 10.5435 pixels mm^{-1}	θ_{max} = 73.7°, θ_{min} = 3.0°
ω scans	*h* = −21 17

Absorption correction: multi-scan

CrysAlis PRO 1.171.38.43c (Rigaku Oxford Diffraction)

$k = -23$ 22

T_{min} = 0.371, T_{max} = 1.000

$l = -21$ 21

37442 measured reflections

Refinement

Refinement on F^2

Secondary atom site location: difference Fourier map

Least-squares matrix: full

Hydrogen site location: mixed

$R[F^2 > 2\sigma(F^2)] = 0.046$

H atoms treated by a mixture of independent and constrained refinement

$wR(F^2) = 0.130$

W = $1/[\Sigma^2(FO^2) + (0.0676P)^2 + 1.1643P]$ WHERE $P = (FO^2 + 2FC^2)/3$

$S = 1.03$

$(\Delta/\sigma)_{max} = 0.001$

10313 reflections

$\Delta\rho_{max}$ = 0.30 e Å^{-3}

603 parameters

$\Delta\rho_{min}$ = −0.36 e Å^{-3}

0 restraints

Extinction correction: none

0 constraints

Extinction coefficient: -

Primary atom site location: iterative

4.) Supplementary References

[S1] D. Franz, S. Inoue, *Chem. Eur. J.* **2014**, *20*, 10645–10649.

[S2] D. Franz, E. Irran, S. Inoue, *Dalton Trans.* **2014**, *43*, 4451–4461.

[S3] M. Tamm, D. Petrovic, S. Randoll, S. Beer, T. Bannenberg, P. G. Jones, J. Grunenberg, *Org. Biomol. Chem.* **2007**, *5*, 523–530.

[S4] D. Franz, E. Irran, S. Inoue, *Angew. Chem. Int. Ed.* **2014**, *53*, 14264–14268.

[S5] *APEX suite of crystallographic software*, APEX 3, Versions 2015-5.2 and 2016.9-0, Bruker AXS Inc., Madison, Wisconsin, USA, 2015/2016.

[S6] *CrysAlisPro*, Versions 1.171.38.43c and 1.171.39.46, Rigaku Oxford Diffraction, Abingdon, Oxfordshire, England, 2015/2018.

[S7] *SAINT*, Versions 8.34A and 8.37A, and *SADABS*, Versions 2014/5 and 2016/2, Bruker AXS Inc., Madison, Wisconsin, USA, 2014/2016.

[S8] G. M. Sheldrick, *Acta Crystallogr. Sect. A* **2015**, *71*, 3–8.

[S9] G. M. Sheldrick, *Acta Crystallogr. Sect. C* **2015**, *71*, 3–8.

[S10] C. B. Hübschle, G. M. Sheldrick, B. Dittrich, *J. Appl. Cryst.* **2011**, *44*, 1281–1284

[S11] *International Tables for Crystallography, Vol. C* (Ed.: A. J. Wilson), Kluwer Academic Publishers, Dordrecht, The Netherlands, **1992**, Tables 6.1.1.4 (pp. 500–502), 4.2.6.8 (pp. 219–222), and 4.2.4.2 (pp. 193–199).

[S12] A. L. Spek, *Acta Crystallogr. Sect. C* **2015**, *71*, 9–18.

[S13] C. F. Macrae, I. J. Bruno, J. A. Chisholm, P. R. Edgington, P. McCabe, E. Pidcock, L. Rodriguez-Monge, R. Taylor, J. van de Streek, P. A. Wood, *J. Appl. Cryst.* **2008**, *41*, 466–470.

[S14] A. L. Spek, *Acta Crystallogr. Sect. D* **2009**, *65*, 148–155.
